# A Combined Spectroscopic and Computational Study on the Mechanism of Iron-Catalyzed Aminofunctionalization of Olefins Using Hydroxylamine Derived N–O Reagent as the “Amino” Source and “Oxidant”

**DOI:** 10.1021/jacs.1c11083

**Published:** 2022-02-04

**Authors:** Sayanti Chatterjee, Ingolf Harden, Giovanni Bistoni, Rebeca G. Castillo, Sonia Chabbra, Maurice van Gastel, Alexander Schnegg, Eckhard Bill, James A. Birrell, Bill Morandi, Frank Neese, Serena DeBeer

**Affiliations:** † Max Planck Institute for Chemical Energy Conversion, Stiftstrasse 34-36, 45470 Mülheim an der Ruhr, Germany; ‡ Max-Planck-Institut für Kohlenforschung, Kaiser-Wilhelm-Platz 1, 45470 Mülheim an der Ruhr, Germany; § ETH Zürich, Vladimir-Prelog-Weg 3, HCI, 8093 Zürich, Switzerland; ∥ Max-Planck-Institut für Kohlenforschung, Kaiser-Wilhelm-Platz 1, 45470 Mülheim an der Ruhr, Germany

## Abstract

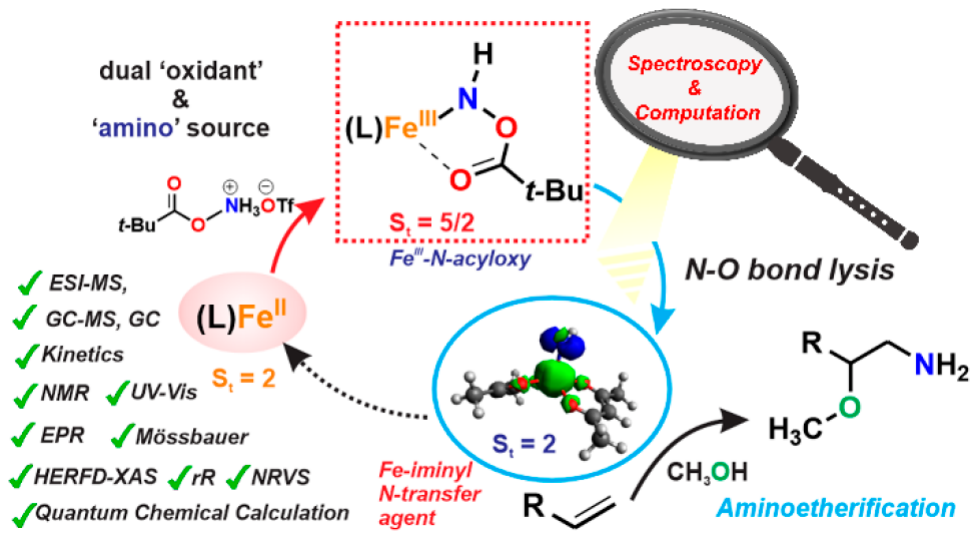

Herein, we study the mechanism of iron-catalyzed direct synthesis of unprotected aminoethers from olefins by a hydroxyl amine derived reagent using a wide range of analytical and spectroscopic techniques (Mössbauer, Electron Paramagnetic Resonance, Ultra-Violet Visible Spectroscopy, X-ray Absorption, Nuclear Resonance Vibrational Spectroscopy, and resonance Raman) along with high-level quantum chemical calculations. The hydroxyl amine derived triflic acid salt acts as the “oxidant” as well as “amino” group donor. It activates the high-spin Fe(II) (*S*_t_ = 2) catalyst [Fe(acac)_2_(H_2_O)_2_] (**1**) to generate a high-spin (*S*_t_ = 5/2) intermediate (**Int I**), which decays to a second intermediate (**Int II**) with *S*_t_ = 2. The analysis of spectroscopic and computational data leads to the formulation of **Int I** as [Fe(III)(acac)_2_-*N*-acyloxy] (an alkyl-peroxo-Fe(III) analogue). Furthermore, **Int II** is formed by N–O bond homolysis. However, it does *not* generate a high-valent Fe(IV)(NH) species (a Fe(IV)(O) analogue), but instead a high-spin Fe(III) center which is strongly antiferromagnetically coupled (*J* = −524 cm^–1^) to an iminyl radical, [Fe(III)(acac)_2_-NH·], giving *S*_t_ = 2. Though Fe(NH) complexes as isoelectronic surrogates to Fe(O) functionalities are known, detection of a high-spin Fe(III)-*N*-acyloxy intermediate (**Int I**), which undergoes N–O bond cleavage to generate the active iron–nitrogen intermediate (**Int II**), is unprecedented. Relative to Fe(IV)(O) centers, **Int II** features a weak elongated Fe–N bond which, together with the unpaired electron density along the Fe–N bond vector, helps to rationalize its propensity for *N*-transfer reactions onto styrenyl olefins, resulting in the overall formation of aminoethers. This study thus demonstrates the potential of utilizing the iron-coordinated nitrogen-centered radicals as powerful reactive intermediates in catalysis.

## 1 Introduction and Background

Amines are found ubiquitously throughout the natural world as key functional groups in amino acids and nucleotide bases, and are fundamental components of pharmaceuticals, agrochemicals, dyes and polymers.^1−6^ Installation of “amino-functionality” remains one of the major challenges in organic synthesis. An attractive approach to address this challenge is the direct catalytic amination of organic molecules and has been the subject of intense research efforts.^7−10^ Nevertheless, most synthetic procedures involve toxic, explosive and/or expensive chemicals and intermediates and, therefore, are not in line with the principles of green chemistry.^11^ Inspired by the widely studied iron-based enzymes, biomimetic complexes have opened new avenues for the oxidation and amination of organic substrates.^12−18^ However, most of the methods developed thus far lead to the installation of a protected form of the amino group, requiring additional and often challenging protecting group manipulations.^19^ Moreover, synthesis of unprotected amino functionality poses another serious challenge of product coordination to the metal catalysts, thereby, leading to catalyst deactivation.

**Scheme 1:**
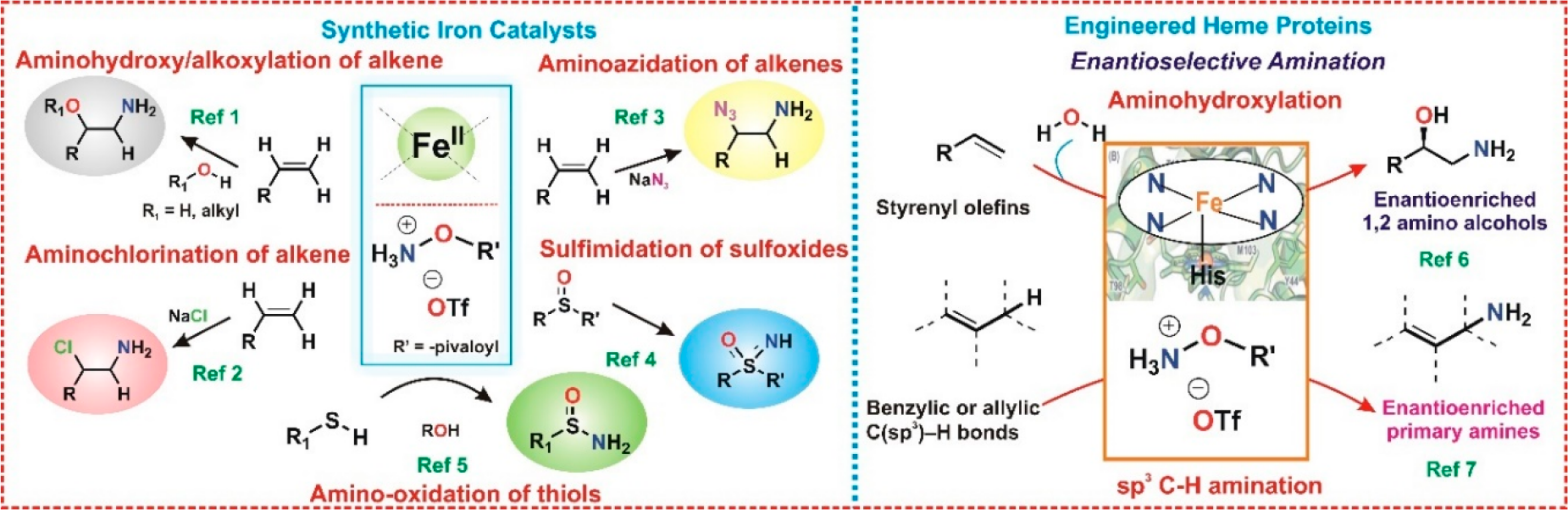
Fe Catalyzed Aminofunctionalization Reactions by Hydroxylamine Derived Reagents (Ref 1,^21^ Ref 2,^22^ Ref 3,^23^ Ref 4,^26^ Ref 5,^25^ Ref 6,^27^ Ref 7^28^)

In order to address these issues, and inspired by the seminal work from Minisci,^20^ Morandi and co-workers have developed a research program focused on iron-catalyzed direct synthesis of unprotected amines (aminofunctionalization of alkenes) using hydroxylamine derived reagents (Scheme 1, left panel).^21−24^ The versatile reactivity of the iron-catalyzed aminofunctionalization was also successfully exploited for heteroatom amination (Scheme 1, left panel).^25,26^ Subsequently, Arnold and co-workers broadened the utility of these hydroxylamine-derived reagents to mimic the non-natural nitrene transfer reaction for enantioselective amination of styrenyl olefins, as well as −C–H bonds of alkanes, catalyzed by engineered hemoproteins (Scheme 1, right panel).^27,28^ However, despite the successful utilization of the iron-catalyzed amination reaction on various organic substrates, the mechanistic pathway and nature of the active species responsible for the aminofunctionalization reaction, whether a free aminium organic radical (NH_3_^+•^)^29−33^ or any iron-based aminating species is involved^16,17,34−36^ remains unknown. To date there has been no spectroscopic or theoretical report on the mechanistic details of the iron-catalyzed reaction by these novel hydroxyl-amine derived reagents. However, control studies have strongly implicated the key role of iron in the amination reaction.^21−25^

In the biological and synthetic realm of oxidation chemistry, high-valent iron-oxo as well as -superoxo, -peroxo and -hydroperoxo species have attracted great interest as key intermediates involved in challenging oxidative transformations.^37^ Unlike oxygenation reactions, metal mediated *N*-transfer reactions to form amines or aziridines are less explored,^38−61^ despite both of these group transfer reactivities having comparable synthetic relevance. The species responsible for such *N*-transfer reactions have been postulated to be metal-nitrenoid-type species, open-shell metal iminyl, or closed-shell terminally bound or bridging imido complexes.^62^ However, many of the reported metal–nitrogen intermediates lack *N*-group transfer reactivity due to strong metal–nitrogen multiple bonds, thereby decreasing the propensity for *N*-group transfer reactivity. There has been significant progress in the isolation of iron–nitrogen intermediates, spanning a large range of oxidation states and electronic structures; Fe(II) *S*_t_ = 0;^55,63^ Fe(III) *S*_t_ = 1/2, *S*_t_ = 3/2;^64−70^*S*_t_ = 5/2;^71^ Fe(III)(^•^NR) *S*_t_ = 2;^72^*S*_t_ = 1;^51^ Fe(IV) *S*_t_ = 0, *S*_t_ = 1;^73−83^ Fe(V) *S*_t_ = 1/2;^84−88^ and even Fe(VI) *S*_t_ = 0 (*S*_t_ = total spin).^88,89^ However, only a handful of the iron–nitrogen intermediates reported above are competent for efficient nitrogen group transfer activity. In fact, studies have revealed that rather subtle changes in the electronic structure and coordination environment can have dramatic effects on *N*-transfer reactivity compared to oxo-transfer.^80,86,90^ Thus, many aspects of nitrogen transfer reactions need to be explored, which likely will unveil a rich and distinctive chemistry compared to the oxygenation/hydroxylation chemistry.^91,92^ Inspired by the rich utility of amination chemistry across various fields of chemical synthesis and catalysis, as well as the intriguing spectroscopic and electronic structure that can be expected for the proposed iron–nitrogen intermediates, we sought to explore the role of iron and the mechanistic details in the catalytic amination reaction using a wide range of spectroscopic methods, as well as computational studies.

**Scheme 2:**
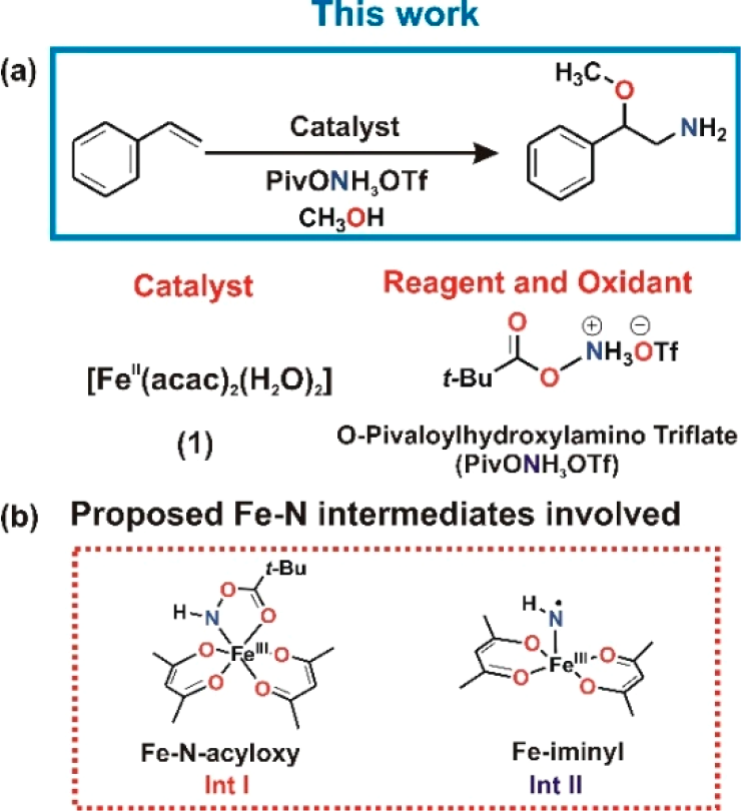
(a) Reaction Studied and Reagent Used in This Study; (b) Proposed Fe−N Intermediates Involved in the Aminofunctionalization Reaction

The present manuscript focuses on elucidating the mechanistic factors that contribute to the success of the iron-catalyzed aminofunctionalization of styrenyl olefins,^21^ using a bench stable hydroxylamine derived triflic acid salt (PivONH_3_OTf, Piv = pivalate, OTf = triflate) as the amine source (Scheme 2a). In general, the process for generation of iron–nitrogen intermediates mostly involve harsh reagents like *N*-tosyl-iodinane or iminoiodinane derivatives or organic azides,^93^ the handling of which often requires special precautions. As such, new developments aimed at more sustainable generation of the aminating intermediates are required. The choice of the bench stable hydroxyl amine derived triflic acid salt (PivONH_3_OTf)^94^ as the “amine source” offers the opportunity to generate iron–nitrogen intermediates under mild conditions. Additionally, apart from being a “free amine source”, the hydroxylamine derived N–O reagents are known to act as internal oxidants thereby opening the scope of versatile iron mediated *N*-transfer reactions to organic molecules.^95^ In this work, kinetic measurements, together with a wide range of analytic and spectroscopic techniques (including ultraviolet–visible absorption spectroscopy (UV–vis ABS), electrospray ionization mass spectrometry (ESI-MS), gas chromatography (GC), gas chromatography and mass spectrometry (GC-MS), nuclear magnetic resonance spectroscopy (NMR), electron paramagnetic resonance spectroscopy (EPR), Mössbauer spectroscopy, Fe high energy resolution fluorescence-detected X-ray absorption spectroscopy (Fe HERFD-XAS), resonance Raman spectroscopy (rR) and nuclear resonance vibrational spectroscopy (NRVS), are used to understand the electronic and geometric features of the reaction intermediates that are generated during the iron-catalyzed aminofunctionalization reaction of olefins. This study provides clear evidence for the involvement of two novel iron–nitrogen intermediates having interesting electronic and bonding properties, which play a pivotal role in controlling the catalytic *N*-transfer activity (Scheme 2b). These intermediates are formed in a stepwise manner upon reaction of an Fe(II) catalyst with the hydroxylamine derived N–O reagent. The experimental results have been correlated to quantum chemical calculations to obtain a deeper insight into the electronic and geometric structure of the putative iron–nitrogen intermediates. This has enabled us to propose a mechanistic pathway for the iron-catalyzed aminomethoxylation of styrenyl type alkenes. The results shed light into the mechanism of N–O bond cleavage to generate active iron–nitrogen intermediates and, as such, have broad implications for the field of synthetic *N*-transfer reactions that are important in countless areas of chemistry.

## 2 Experimental and Spectroscopic Results

### 2.1 Synthesis of the Fe(II) Catalyst (**1**) and Hydroxylamine Derived Reagent (PivONH_3_OTf)

The Fe(II) catalyst used in this study [Fe^II^(acac)_2_(H_2_O)_2_] (**1**) was synthesized in a reliable way with high purity and good yield following slight modification of the previously reported literature procedure.^96^ (see SI for detailed synthesis). The hydroxyl amine derived reagent PivONH_3_OTf (Piv = pivalate) was synthesized following the reported standard procedure in high yield and purity.^94^

#### 2.2.1 UV–vis Absorption Spectroscopy (ABS)

**Figure 1:**
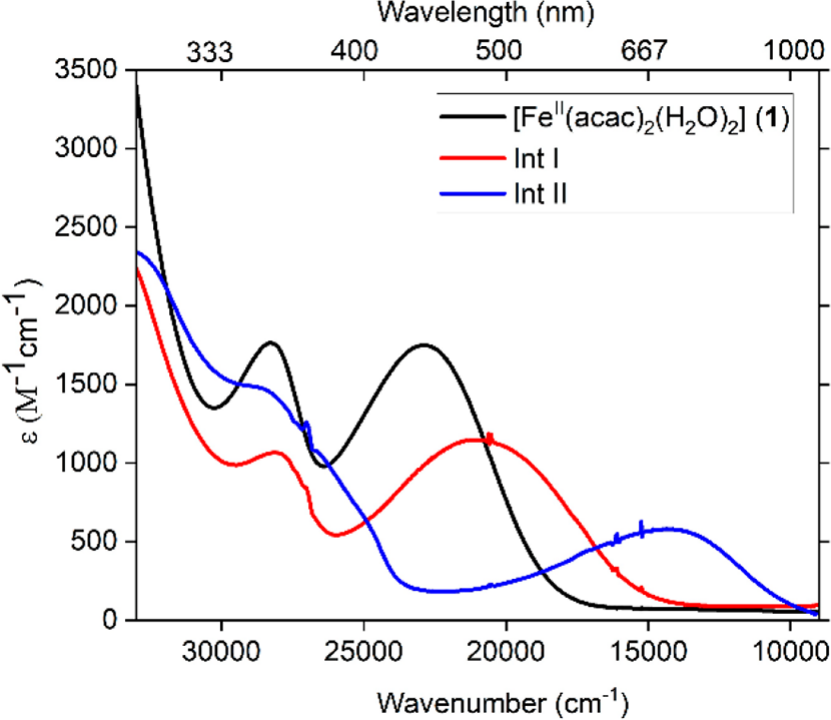
UV–vis absorption spectra of 0.25 mM solution of **1** (black); after addition of 2.5 equiv of PivONH_3_OTf within 15 min (**Int I**, red) and 90 min (**Int II**, blue) to **1** in CH_2_Cl_2_ at 293 K.

Reaction of PivONH_3_OTf (2.5 equiv) with a solution of [Fe^II^(acac)_2_(H_2_O)_2_] (**1**) (Figure 1, black spectrum) in dichloromethane (yellow solution) (λ_max_ = 353 nm, ε_353_ = 1776 M^–1^cm^–1^ and λ_max_ = 437 nm, ε_437_ = 1751 M^–1^ cm^–1^, black spectrum) at room temperature (293 K) generated a wine-red species (**Int I**) with absorption choromophores at λ_max_ = 358 nm (ε_358_ = 1056 M^–1^ cm^–1^) and λ_max_ = 480 nm (ε_480_ = 1156 M^–1^ cm^–1^) (Figure 1, red spectrum). Compared to the precursor Fe^II^(acac)_2_(H_2_O)_2_ (**1**), the resulting chromophores for **Int I** exhibited a bathochromic shift (Figure 1). This wine red **Int I**, when kept under Ar (or even in the presence of air), is then converted to a purple species (**Int II**) within 90 min with a weaker and broader absorption chromophore at λ_max_ = 700 nm (ε_700_ = 568 M^–1^ cm^–1^) (Figure 1, blue spectrum). The UV–vis absorption spectra were deconvoluted with Gaussian bands for analysis, and a detailed assignment has been made on the basis of our quantum chemical calculations (see SI, Figure S2, Table S1 and computational section).

#### 2.2.2 Kinetic Analysis from Absorption Spectroscopy

**Figure 2:**
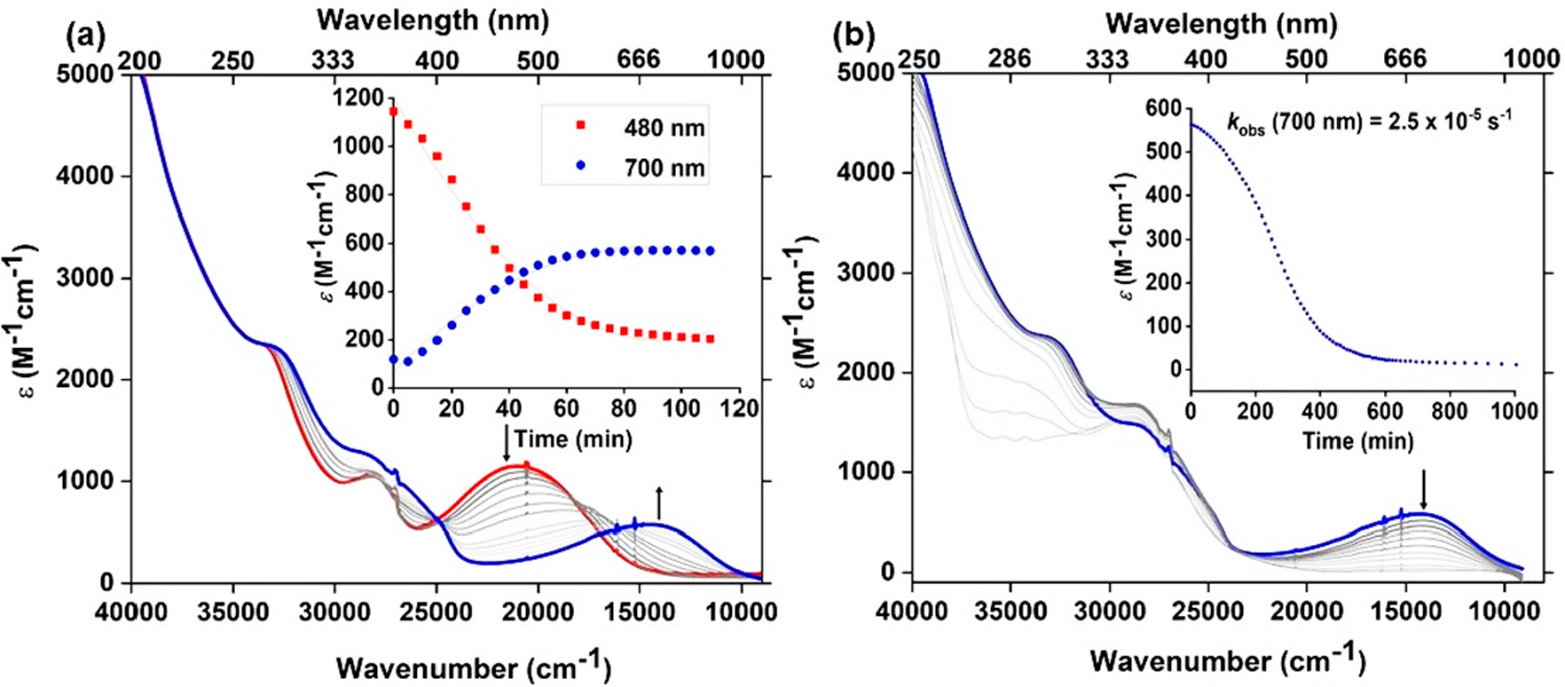
(a) Time dependent optical spectral changes in the reaction of Fe^II^(acac)_2_(H_2_O)_2_ (**1**) (0.25 mM) with PivONH_3_OTf (2.5 equiv) in CH_2_Cl_2_ at 293 K. The inset shows the time course monitored by the absorbance change at 480 nm for the decay of **Int I** and at 700 nm for the formation of **Int II** (Step 1). (b) Optical spectral changes corresponding to the self-decay of **Int II** at 293 K. Inset shows the time for the decay of the band at 700 nm of **Int II** (Step 2).

The rate of the reaction of [Fe^II^(acac)_2_(H_2_O)_2_] **1** with PivONH_3_OTf was then monitored more closely using a diode array spectrophotometer. The formation of **Int I** from the precursor Fe(II) complex (**1**) upon addition of the aminating agent (PivONH_3_OTf) was instantaneous at room temperature. However, the peak formed at 480 nm for **Int I** decayed with a first-order rate constant of 2.2 × 10^–4^ s^–1^ and *t*_1/2_ of 35 min (Figure 2a, red trace). Simultaneously, another peak at 700 nm appeared with a first-order rate constant of 2.9 × 10^–4^ s^–1^, which corresponded to the formation of **Int II** within 90 min (Figure 2a, blue trace). In the second step (Figure 2b) after 90 min, the peak at 700 nm for **Int II** decayed, with a first-order *k*_obs_ value of 2.5 × 10^–5^ s^–1^ and a *t*_1/2_ of 260 min (Figure 2b). The final solution was pale yellow with no significant chromophore in the visible range. Similar optical patterns were also observed in toluene as the solvent.

##### Kinetics in the Presence of Substrate

Styrenyl olefins undergo catalytic aminomethoxylation in the presence of iron catalysts and PivONH_3_OTf to regioselectively form 2-methoxy-2-phenylethan-1-amine,^21^ and in this work using **1** as the catalyst, we obtained an isolated yield of 60% of the aminomethoxylated product from styrene (Scheme 1a and Figure S1, SI). We followed the rate for the amino-methoxylation of styrene by **1** in the presence of PivONH_3_OTf in CH_2_Cl_2_/CH_3_OH (3:1) solvent mixture by a diode array spectrophotometer. In the presence of styrene as a substrate, the decay profile for **Int I** remained unchanged; however, the decay of the band at 700 nm assigned to **Int II** was almost two times faster (4.8 × 10^–5^ s^–1^), compared to its self-decay rate under similar reaction conditions (Table S2, SI), suggesting involvement of **Int II** in the amino transfer step to styrene. The reaction followed pseudo-first-order behavior in the presence of excess styrene as a substrate (Figure S3 and Table S2, SI). The observed rate constant for decay of **Int II** was found to depend linearly on the substrate concentration enabling extraction of the second-order rate constant (*k*_2_) (Figure S3). The amino-methoxylation of styrenyl olefins catalyzed by **1** and PivONH_3_OTf was also investigated with various *para*-X-substituted styrenes (X = OMe, Me, H, Cl, and Br), which exhibited a higher rate for electron-donating styrenes (see SI; for complete analyses and discussion, Figures S3 and S4 and Tables S2–S6, and for Hammett analyses Figures S5 and S6 and Tables S7 and S8).

### 2.3 ESI-Mass Spectroscopy

**Scheme 3:**
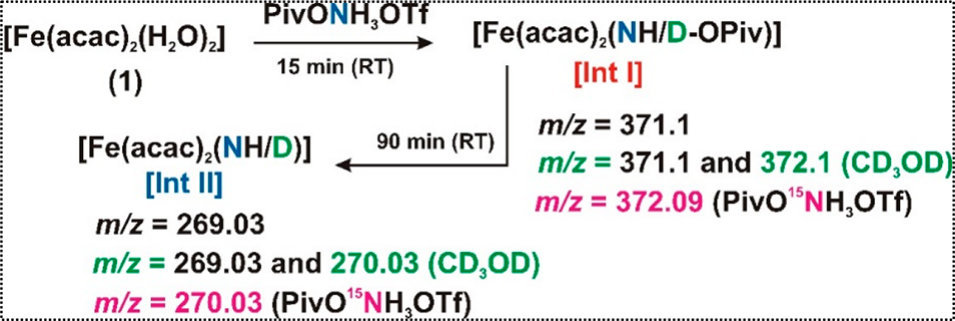
ESI-MS of the Reaction Solution of **1** and PivONH_3_OTf in CH_2_Cl_2_/CH_3_OH Solvent Mixture

Analyses of the reaction solution of **Int I** by electrospray ionization mass spectrometry (ESI-MS) reveals an ion peak at *m*/*z* = 371.1, attributable to [Fe(acac)_2_(PivONH) + H^+^] [C_15_H_25_FeNO_6_ + H^+^] (Scheme 3 and Figure S8, SI). For **Int II**, the ESI-mass spectrum of the reaction solution shows ion peaks at *m*/*z* = 269.03 and *m*/*z* = 270.04 with the isotope distribution patterns attributable to [Fe(acac)_2_(NH)]^+^ and [Fe(acac)_2_(NH) + H^+^] (Scheme 3 and Figure S10), implicating the loss of the O-pivaloyl group (−OPiv) from **Int I**. Performing the same reaction in a deuterated solvent reveals an exchangeable proton in the NH species coordinated to Fe(acac)_2_ for both **Int I** (Scheme 3, Figure S9) and **Int II** (Scheme 3, Figure S11). Further ^15^N labeling experiments confirmed that the hydroxyl amine derived reagent (PivO^15^NH_3_OTf) is the source of nitrogen in the putative iron–nitrogen intermediates (**Int I** and **Int II**) (Scheme 3, Figures S12 and S13).

To gain more insights into the electronic structure and nature of the iron species involved in the aminofunctionalization reaction of styrene, we utilized a range of spectroscopic techniques together with quantum chemical calculations in order to obtain a detailed understanding of the generated intermediates and the reaction mechanism.

### 2.4 EPR Spectroscopy

**Figure 3:**
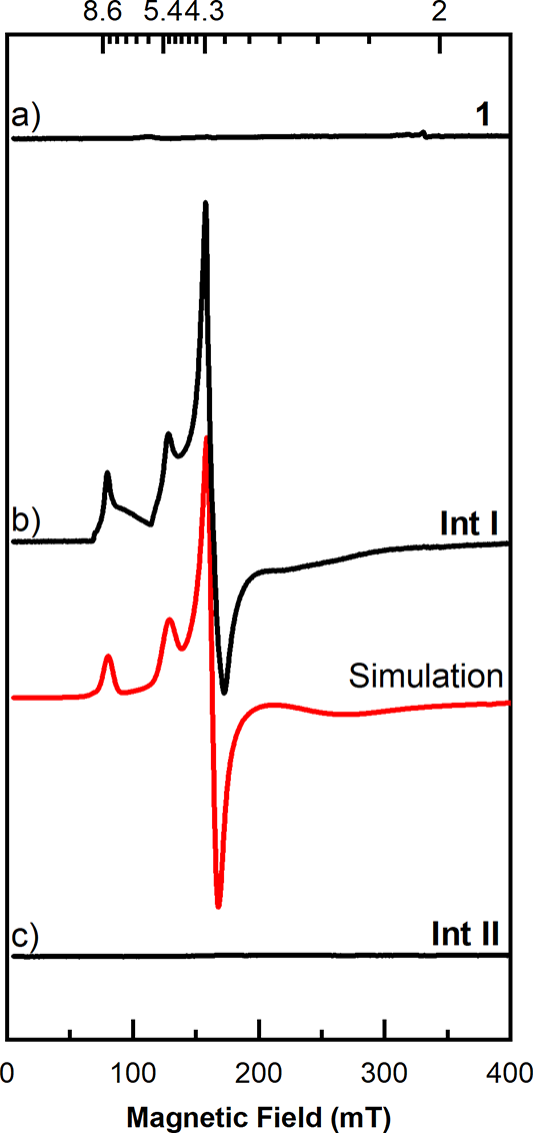
Experimental (black) and simulated (red) X-band CW EPR spectra (*T* = 10 K) of (a) Fe^II^(acac)_2_(H_2_O)_2_ (**1**) precursor (1 mM) dissolved in a mixture of dichloromethane and toluene, (b) the same sample mixed with aminating agent (PivONH_3_OTf, ∼3 equiv) after 15 min (**Int I**) and (c) after 90 min (**Int II**). All spectra have been measured with identical EPR detection parameters given in the SI. The simulation of the **Int I** (red line) is a superposition of two different *S*_*t*_ = 5/2 subspectra with the following spin Hamiltonian parameters: Component A (with approximately 60% abundance): *g* = 2, *D* = 0.4 cm^–1^, *E*/*D* = 0.145, *D* and *E* strain of Δ*D* = 0.05 cm^–1^ and Δ*E* = 0.025 cm^–1^, respectively and a Lorentzian line width broadening of 1 mT and Component B (with approximately 40% abundance): *g* = 2, *D* = 0.4 cm^–1^, *E*/*D* = 0.33, Δ*D* = 0.3 cm^–1^ and Δ*E* = 0.13 cm^–1^ and a Lorentzian line broadening of 5 mT.

The precursor complex Fe^II^(acac)_2_(H_2_O)_2_ (**1**) dissolved in dichloromethane/toluene (1:1 ratio by volume) is EPR-silent at X-band frequencies (9.6 GHz, Figure 3a), which is in accordance with the integer spin state of Fe(II). In contrast, a mixture of **1** with aminating agent (PivONH_3_OTf) in a 1:3 ratio, frozen after 15 min, showed a broad EPR spectrum with resolved peaks at effective *g*-values, *g*_eff_ = 9.3, 8.6, 5.4, 4.3 and a broad unresolved spectral region ranging from *g*_eff_ ≈ 4 to 2 (Figure 3b, black line and Figure S17, SI). Simulations of the EPR spectrum obtained after mixing with the aminating agent indicated the formation of a variety of similar *S*_t_ = 5/2 species. All components of this spectrum could be simulated reasonably well (Figure 3b, red line, details given below and Figure S18, SI) by a superposition of two major high spin (*S*_t_ = 5/2) components with different rhombic zero field splitting (ZFS). Differences in the spectral shapes of the two main components could be identified with slightly different rhombic ZFS, presumably arising from site-to-site variation in the iron coordination environment. Site-to-site disorder is also reflected in the broad distribution of the ZFS values (*D* and *E* strain), which significantly reduce the resolution in the *g*_eff_ ≈ 4 to 2 spectral region (see Figures S18−S19 and detailed discussion in the SI). EPR spin quantification of the *S*_t_ = 5/2 spectrum yielded ∼65–75% abundance with respect to the total iron content of the starting precursor **1** (see SI). An identical spectrum, however, with lower intensity was observed for samples immediately frozen after mixing of the precursor with the aminating agent (see Figure S17). Due to its time evolution, the *S*_t_ = 5/2 spectrum is associated with the first intermediate **Int I** (also identified by the UV–vis measurements). It is our hypothesis that both of these components belong to the same intermediate and reflect a microheterogeneity in the sample. There is literature precendence for such a situation in which a single Fe(III) S = 5/2 species gives rise to two different subspectra due to site-to-site disorder.^97,98^ Based on the Mössbauer spectra shown in the next section, the *S*_t_ = 5/2 spectra of **Int I** can be assigned to high spin Fe(III) with closed-shell ligands, in agreement with the literature.^99−104^ Finally, a sample frozen after 90 min incubation was EPR-silent again (Figure 3c) and assigned to **Int II** (also confirmed from the UV–vis measurements).

### 2.5 Mössbauer Spectroscopy

**Figure 4:**
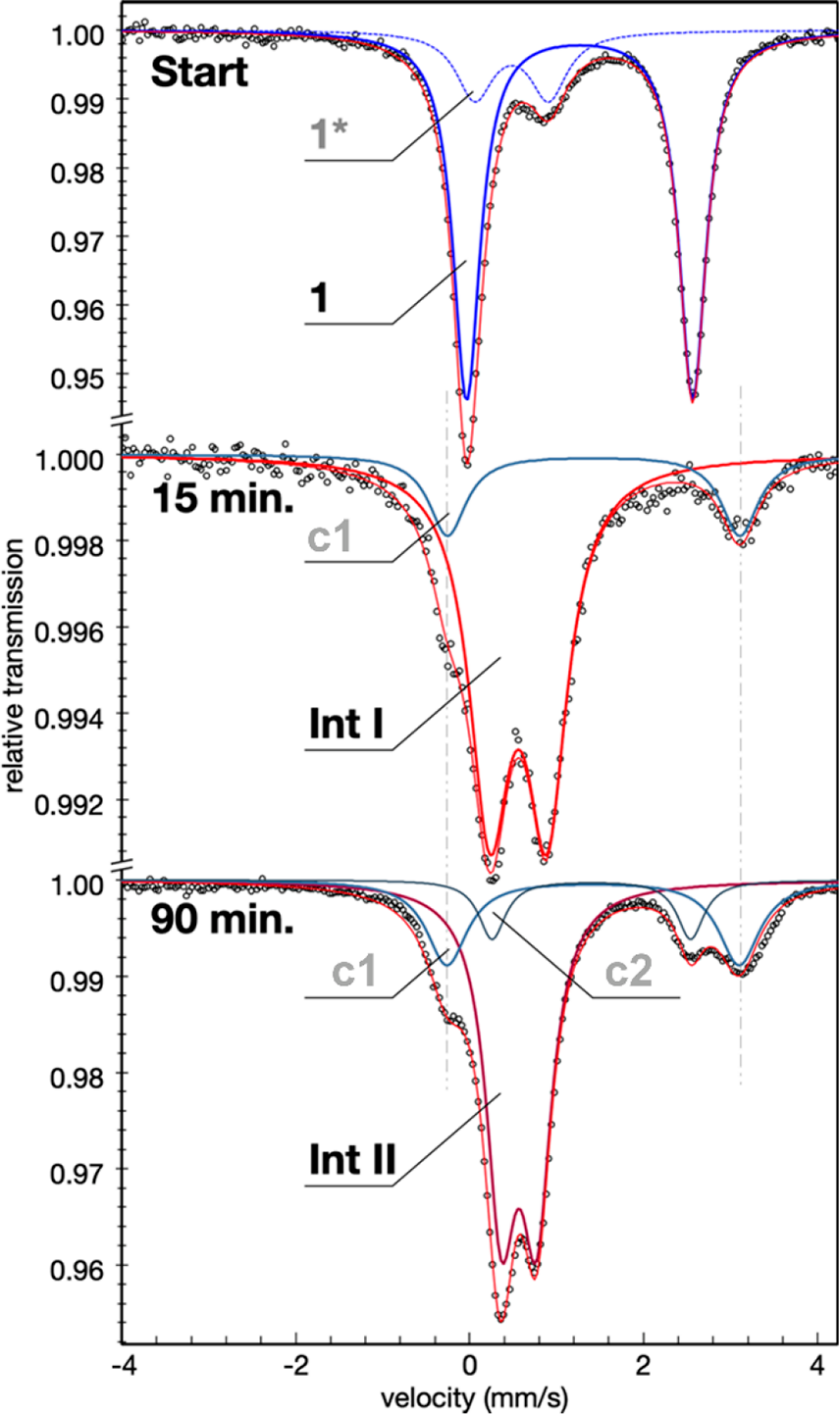
Zero field Mössbauer spectra recorded at 80 K with 2 mM ^57^Fe enriched [Fe(acac)_2_(H_2_O)_2_] (**1**) in frozen toluene solution (top) and of corresponding samples mixed with PivNH_3_OTf after 15 min (middle) and 90 min incubation time (bottom). The solid and dashed lines represent fits with Lorentzian doublets, and the thin red lines are the sums of the subspectra.

To gain more insight into the electronic structure of the iron–nitrogen intermediates involved in the amination reaction, Mössbauer experiments were performed using a ^57^Fe labeled version of **1**. The zero-field ^57^Fe Mössbauer spectrum of **1** in toluene at 80 K displays a major quadrupole doublet with ca. 80% relative intensity (Figure 4 top, Table 1). The high isomer shift (δ) and large quadrupole splitting (Δ*E*_Q_) of the subspectrum confirm unambiguously the high-spin Fe(II) oxidation state of [Fe^II^(acac)_2_(H_2_O)_2_] (**1**). Moreover, six-coordination can be inferred from the isomer shift. A minor (20%) doublet found in the spectrum is assigned to a contaminant (1*) from anhydrous [Fe^II^(acac)_2_] in dimeric form^105^ (see Figure S23).

**Table 1:** Mössbauer Parameters Obtained from the Fits (in mm/s)

Preparation time	Component	δ	Δ*E*_*Q*_	Abundance
Start	**1**	1.26	2.60	80%
	1*	0.48	0.85	20%
15 min	**Int I**	0.55	0.66	83%
	**c1**	1.42	3.36	17%
90 min	**Int II**	0.57	0.40	66%
	**c1**	1.42	3.36	23%
	**c2**	1.40	2.28	11%

Addition of PivONH_3_OTf to precursor **1** within 15 min preparation leads to a completely new Mössbauer spectrum with two subspectra (Figure 4 middle, Table 1 and Figure S21). The major (83%) component has low isomer shift and quadrupole splitting, revealing formation of a high-spin Fe(III) species. In conjunction with the corresponding EPR measurements assigned to *S*_t_ = 5/2 (Figure 3b), we can conclude that the 83% subspectrum represents **Int I**, which hence is a mononuclear ferric high-spin complex with closed-shell ligands and the spin (*S*_t_ = 5/2) centered on iron. Apparently, in the first step of the reaction a part of the aminating agent (PivONH_3_OTf) oxidizes ferrous **1** to generate ferric **Int I**, presumably in a sacrificial process (*vide infra*). The (micro) heterogeneity of **Int I** seen in the EPR spectra is not resolved in the Mössbauer spectrum (Figure 4 middle), but the lines of the ferric subspectrum in **Int I** are remarkably broad (0.57 mm/s vs 0.24 mm/s resolution). This broadening may be assigned to the same site-to-site disorder in the coordination environment, which leads the strain of the *D* and *E* parameters observed in the **Int I** EPR spectra. Moreover, a new residual component **c1** (17%) is observed for the 15 min preparation, which due to the high isomer shift is assigned to another ferrous high-spin species, but distinctly different from the starting compound **1** or its anhydrous [Fe^II^(acac)_2_] contaminant 1*. We can exclude that the new ferrous component could correspond to one of the *S*_t_ = 5/2 EPR subspectra of **Int I**, hypothetically possible only if an oxidized ligand radical would be present. However, in that case also antiferromagnetic spin coupling would be expected, yielding total spin *S*_t_ = 3/2 which was not observed in the EPR spectra. Such a spin coupled species would also exhibit larger zero-field splitting than ferric **Int I**, due to a larger single-ion contribution from Fe(II). Instead, as explanation for **c1**, we suggest erratic formation of another high spin ferrous species, during the sacrificial catalyst activation pathway, and the presence of several possible coordinating species in the reaction solution like H_2_O, OTf^–^, MeOH, *t*BuCOO^–^ make it challenging to predict its unambiguous composition. This side product persists in the reaction mixture and does not take part in the subsequent reaction course as is shown in the next step.

The Mössbauer spectrum of a sample frozen 90 min after adding PivONH_3_OTf to **1** (Figure 4 bottom, Table 1 and Figure S22, SI) shows complete conversion of the ferric intermediate **Int I**, whereas the ferrous side product **c1** from above remained (slight increased to 23%). Also a second ferrous residual **c2** with a similar high isomer shift but lower quadrupole splitting was formed in a small amount (11%), but the Mössbauer spectrum is dominated by a new quadrupole doublet with a low isomer shift and quadrupole splitting (67%, Table 1). The Mössbauer parameters differ from those of **Int I**, but are also in accord with high-spin Fe(III), presumably with a slightly different coordination sphere. As the 90 min sample, according to UV–vis and EPR spectra, should contain primarily **Int II**, we assign the new ferric subspectrum to **Int II**. Hence, the EPR-silent ground state of **Int II** can arise only from spin coupling of the ferric central ion with a ligand radical. Based on ESI-mass spectrometry (Scheme 3), **Int II** has a (C_10_H_15_FeNO_4_) [Fe(acac)_2_(NH)]^+^ composition, which could be ascribed to either an Fe(IV)=NH or an Fe(III)–NH^*•*^ radical species, with either formulation giving rise to an overall integer spin. However, as the δ value of 0.57 mm/s is distinctly beyond the range of isomer shifts known for (high-spin) Fe(IV) oxo^106−108^ or imido species^82^ (−0.19 to 0.35 mm/s), the iron center of **Int II** is more likely an Fe(III) rather than an Fe(IV) species. Thus, **Int II** may be best assigned as a high-spin Fe(III) species (*S* = 5/2) coupled to the −NH^*•*^ radical (*S* = 1/2) to form an EPR silent complex integer total spin *S*_t_ = 2 ground state. Interestingly, the Mössbauer parameters agree well with the literature values reported of a comparable Fe(III) species (*S* = 5/2) coupled to a superoxo radical (*S* = 1/2) with δ = 0.50 mm/s, *ΔE*_Q_ = 0.33 mm/s.^109^

### 2.6 X-ray Absorption Spectroscopy (XAS)

**Figure 5:**
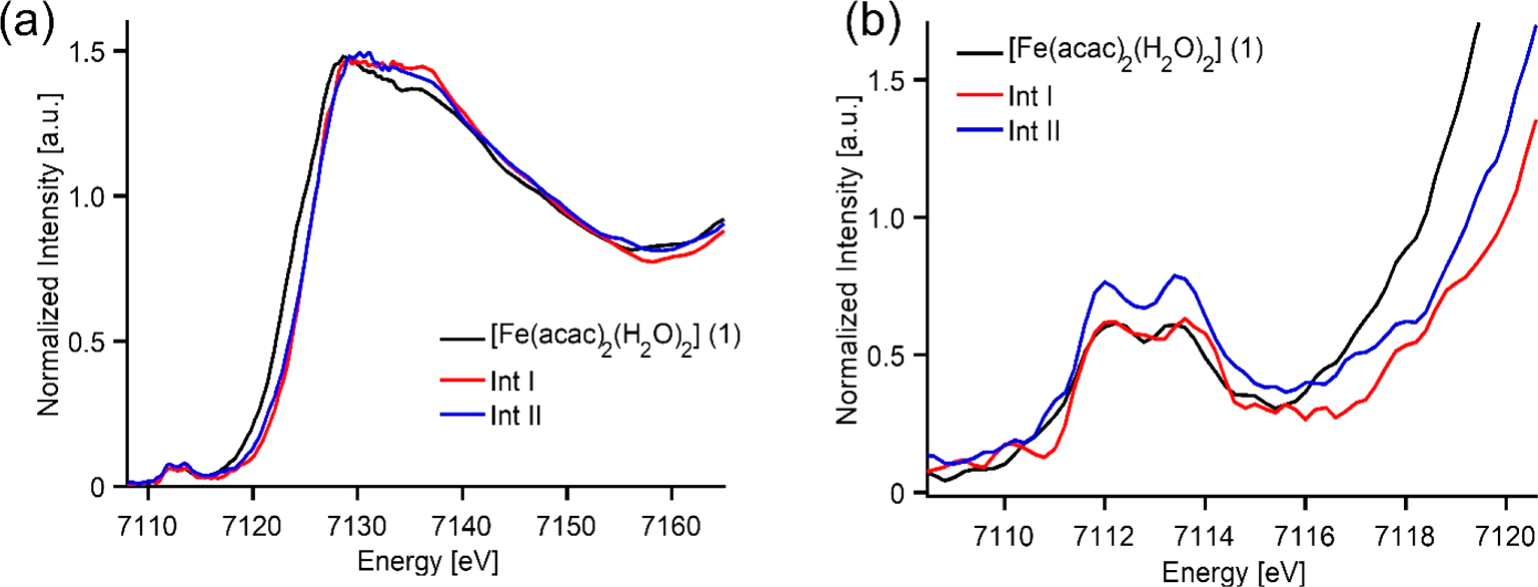
(a) Fe Kβ_1,3_ HERFD-XAS spectra of precursor **1**, **Int I** and **Int II** (left) and (b) expanded view of the pre-edge region (right).

X-ray absorption spectroscopy (XAS) of the precursor **1**, **Int I** and **Int II** were measured to further assess the metal oxidation state and the electronic structure of this series. Figure 5 shows the Fe Kβ_1,3_ HERFD-XAS (High Energy Resolution Fluorescence Detected - X-ray Absorption Spectroscopy)^110,111^ of all the compounds. Kβ_1,3_ HERFD-XAS of transition metals offers a higher resolution than conventional XAS measurements by utilizing a detection mode which minimizes the 1s core-hole broadening.^112,113^

In Figure 5a there is an ∼1.3 eV energy shift in the rising edge position on going from precursor (**1**) to intermediates (**Int I** and **Int II**), while both **Int I** and **Int II** spectra overlap in energy. The edge position of an XAS is dominated by the dipole allowed 1s → 4p transition and its position is generally considered to track changes in the oxidation state. The [(Fe(acac)_2_(H_2_O)_2_] (**1**) precursor has an Fe-edge at lower energies, corresponding to an Fe(II) oxidation state, while both **Int I** and **Int II** are shifted toward higher energies, consistent with an Fe(III) oxidation state in both cases. These observations further support the results obtained from EPR and Mössbauer experiments, where we observed precursor **1** to be a high spin Fe(II) (*S*_t_ = 2) EPR silent species, whereas **Int I** was assigned to a high spin Fe(III) (*S*_t_ = 5/2) species. For **Int II**, an EPR silent species, the possibility of it being an Fe(IV)=NH or an Fe(III)–NH^•^ radical (both having overall integer spin) was questionable. However, the isomer shift value obtained from the Mössbauer experiment along with the XAS rising edge data strongly supports the Fe(III) oxidation state and favors an *Fe(III)-NH*^•^*radical species for****Int II****rather than an Fe(IV)=NH.*

The information contained in the Fe Kβ_1,3_ HERFD-XAS spectra can be further investigated by focusing on the so-called pre-edge region, which is formally a quadrupole allowed and dipole forbidden transition, having less intense features compared to the edge region. As the pre-edge absorption corresponds to 1s → 3d transitions, this spectral region intrinsically contains information on the lowest unoccupied or singly occupied molecular orbitals (LUMO and SOMO, respectively) and, therefore, directly reflects the metal center’s electronic and geometric structure. The pre-edge features can be rationalized in the one-electron picture with inclusion of ligand field effects. In an O_h_ coordination environment, the low energy feature is due to 1s → 3d-t_2g_, while the high energy feature is due to 1s → 3d-e_g_ transitions,^114,115^ and therefore the energy difference between these two peaks reflects the change in ligand field splitting. All three components (**1**, **Int I** and **Int II**) exhibit pre-edge regions between 7110 and 7115 eV, as shown in Figure 5b at almost similar energy (Table 2), which might be attributed to the fact that the difference in coordination, ligand field and ligand identity can counteract the changes in the spectra due to oxidation, as previously reported.^116^ However, the energy splitting of the pre-edge can be dissected into two different features split by ∼1.2–1.5 eV, which increases by 0.2 eV on going from precursor **1** to **Int I** and decreases by 0.3 eV on going from **Int I** to **Int II** (Table 2). In the pre-edge spectra of the series (**1**, **Int I** and **Int II**, Figure 5b), we observed that both precursor **1** and **Int I** share a similar pre-edge intensity, while the pre-edge intensity increases in **Int II**. The intensity of the pre-edge can be modulated by a decrease in the metal symmetry, which consequently increases the dipole contributions due to the presence of 3d–4p mixing.^117^ The relative intensities between the two pre-edge features are found to be similar across the series on going from precursor **1** to **Int I** suggesting that a pseudo-O_h_ environment may be conserved for both. On the other hand, there is a decrease of pre-edge intensity for **Int I** (∼36%) compared to **Int II**, which could be attributed to an increased centrosymmetry of **Int I** compared to **Int II**, resulting from an increased coordination number (*vide infra*).^112^ The increase in relative intensities between the two pre-edge features for **Int II** suggests a higher level of 3d–4p mixing, which could be a consequence of a shorter Fe–N bond (*vide infra*).^89^ Critically, the strength of the ligand interactions could influence the pre-edge intensity, with strongly interacting ligands imparting more significant distortions from centrosymmetry—and thus more intense pre-edges.^118^ Overall, the Fe HERFD-XAS data have shown that while precursor **1** contains Fe(II), **Int I** and **Int II** contain Fe(III) and a pseudo O_h_ environment is maintained across the series with probably a higher distortion for **Int II**, given its higher pre-edge intensity. To obtain further insight into the origins of the pre-edge energy and intensity changes, a systematic time-dependent density functional theory (TD-DFT) study was undertaken to explore the full range of possible binding modes of **1**, **Int I** and **Int II** and their influence on the HERFD-XAS spectra (*vide infra*).

**Table 2:** Fe Kβ_1,3_ HERFD-XAS Experimental Parameters for **1**, **Int I** and **Int II**^a^

Entry	Reaction Component	IWAE (eV)	Experimental pre-edge fitted area	Δ*E* (eV)
1	**1**	7112.95	25	1.3
2	**Int I**	7112.88	22	1.5
3	**Int II**	7112.79	32	1.2

a Δ*E* describes the difference between the two peak maxima in eV.

### 2.7 Vibrational Spectroscopy

#### 2.7.1 resonance Raman Spectroscopy (rR)

resonance Raman (rR) experiments were performed with frozen dichloromethane solutions of **1**, **Int I** and **Int II** at 100 K. In a rR measurement, chromophore vibrations are selectively enhanced, depending on the nature of electronic transition. As such, it is a beautiful way to obtain insight into the nature of the electronic transitions, as well as the structure of the underlying chromophore. Laser excitations for the resonance Raman experiments were selected based on the corresponding absorption spectra (Figure 1).

For **1**, at an excitation wavelength of 491 nm, an intense vibration is observed at 450 cm^–1^ that undergoes a red shift to 460 cm^–1^ for **Int I**. For **Int II** (Figure S26, SI), laser excitation at 660 nm gave resonance-enhanced bands at 433 cm^–1^ along with an intense band at 462 cm^–1^ and comparatively weaker bands at 528 and 612 cm^–1^. Both the rR peaks at 462 and 612 cm^–1^ of **Int II** were slightly sensitive to a ^15^N isotopically enriched reagent (PivO^15^NH_3_OTf) with a shift of around 4 cm^–1^. Assignment of each of the vibrational modes observed has been further explored and discussed in detail after correlating with the quantum chemical calculations (see the computational discussion section of the manuscript and Supporting Information).

#### 2.7.2 Nuclear Resonance Vibrational Spectroscopy (NRVS)

^57^Fe nuclear resonance vibrational spectroscopy (NRVS) was utilized for ^57^Fe labeled precursor **1**, **Int I** and **Int II** (see SI Section, Figure S27 and Table S9). ^57^Fe nuclear resonance vibrational spectroscopy (NRVS) relies on the inelastic absorption of 14.4 keV synchrotron radiation by ^57^Fe nuclei.^119^ In conventional Mössbauer spectroscopy, the recoil-free absorption of photons is observed. However, in NRVS, the recoil fraction is analyzed, providing information on the ligand coordination of ^57^Fe nuclei.^120^ An important advantage of this technique is that only vibrational modes containing significant ^57^Fe motion are observed, and the extent of ^57^Fe motion is proportional to the signal intensity.^121−124^ This makes the technique complementary to infrared and/or resonance Raman spectroscopies since modes that are not observed in these techniques may be observed in NRVS. The NRVS experimental data measured for precursor **1**, **Int I** and **Int II** (Figure S27, and Table S9, SI) are in line with those obtained from rR (Figure S26, SI). There is a shift of the NRVS peak around at 440 cm^–1^ for precursor **1** to 458 cm^–1^ for **Int I** which is further shifted to 462 cm^–1^ for **Int II** suggesting these stretches to be associated with the ^57^Fe movement (Figure S27, and Table S9, SI). To understand the origin of the experimentally observed NRVS bands, computational calculations were undertaken, to correlate the experimentally observed vibrational stretches (see computational section of the manuscript and Supporting Information).

Thus, we have used a wide range of analytic and spectroscopic techniques to probe the electronic and geometric structures of precursor **1**, **Int I** and **Int II**, detected as reaction components for aminomethoxylation of styrene using PivONH_3_OTf as the aminating reagent. From the results so far, it is clear that in order to shed more light on the detailed electronic and geometric structure of the series [**1** (*S*_t_ = 2), **Int I** (*S*_t_ = 5/2) and **Int II** (*S*_t_ = 2)], it is necessary to develop the full information content of spectra obtained from the different spectroscopic techniques. Hence quantum mechanical calculations and *ab initio* ligand field theory have been utilized to obtain a vivid picture of the electronic structure of the intermediates and to connect this to the mechanism.

## 3 Computational Calculations: Correlation to Spectroscopy

All calculations were carried out with the ORCA program package^125^ version 4.2.1. Density functional theory was used with the B3LYP functional^126−129^ together with Grimme’s D3 dispersion correction^130^ with Becke-Johnson damping.^131^ The Ahlrichs def2-TZVP basis set^132^ was used. To speed up the calculations, the resolution of identity^133^ was invoked in the Split-RI-J variant.^134^ In addition, the RIJCOSX approximation^135^ to the exchange integrals was used together with the corresponding auxiliary basis set.^136^ Equilibrium geometries were proven to be real minima on the Potential Energy Surface (PES) by the absence of imaginary frequencies, while transition states were proven to be first-order saddle points on the PES by the presence of one imaginary frequency. For geometry optimizations implicit solvation was included via the C-PCM model^137^ (Toluene) together with the Gaussian charge scheme.^138,139^ For details of the computational methodology, see Supporting Information.

### 3.1 Results and Discussion

In general, insight into a reaction mechanism can be gained through a careful analysis of the computed geometric and electronic structures of the intermediates. The calculation of observables such as the spectroscopic and kinetic properties can serve as important guides in validating the results of calculations and identifying the nature of the observed intermediates.^140^ Thus, correlation of the experimental results to the theoretical calculations are pursued in the subsequent section for electronic and structural elucidation of the reaction components (see Table S33, SI for a comparative overview). We first apply this approach to the precursor complex **1** (see SI, computational section for details), to establish a benchmark for the correlations before extending this analysis to the geometric and electronic structures of the reaction intermediates (**Int I** and **Int II**), which are discussed in the next part of the manuscript.

#### 3.1.1 Preferred Model for the Precursor [Fe(acac)_2_(H_2_O)_2_] (**1**)

From the correlation of the experimental observables (like UV–vis ABS, Mössbauer Spectroscopy, resonance Raman, NRVS and Kβ_1,3_ HERFD XAS) with calculated parameters for **1** (see computational section of SI and Figure S34, for an overview), it becomes evident that the precursor **1** is an *S*_t_ = 2, high spin Fe(II) species, with a distorted O_h_ geometry, coordinated by two monoanionic acac ligands in a bidentate fashion, and two coordinated water molecules either in *cis* disposition (**1-***cis* model) or *trans* disposition (**1-***trans* model) and an equilibrium between the **1-***cis* and **1-***trans* isomer exist in solution.

### 3.2 The First Intermediate: **IntI**

#### 3.2.1 Spin State Energetics and Geometric Models of **IntI**

**Figure 6:**
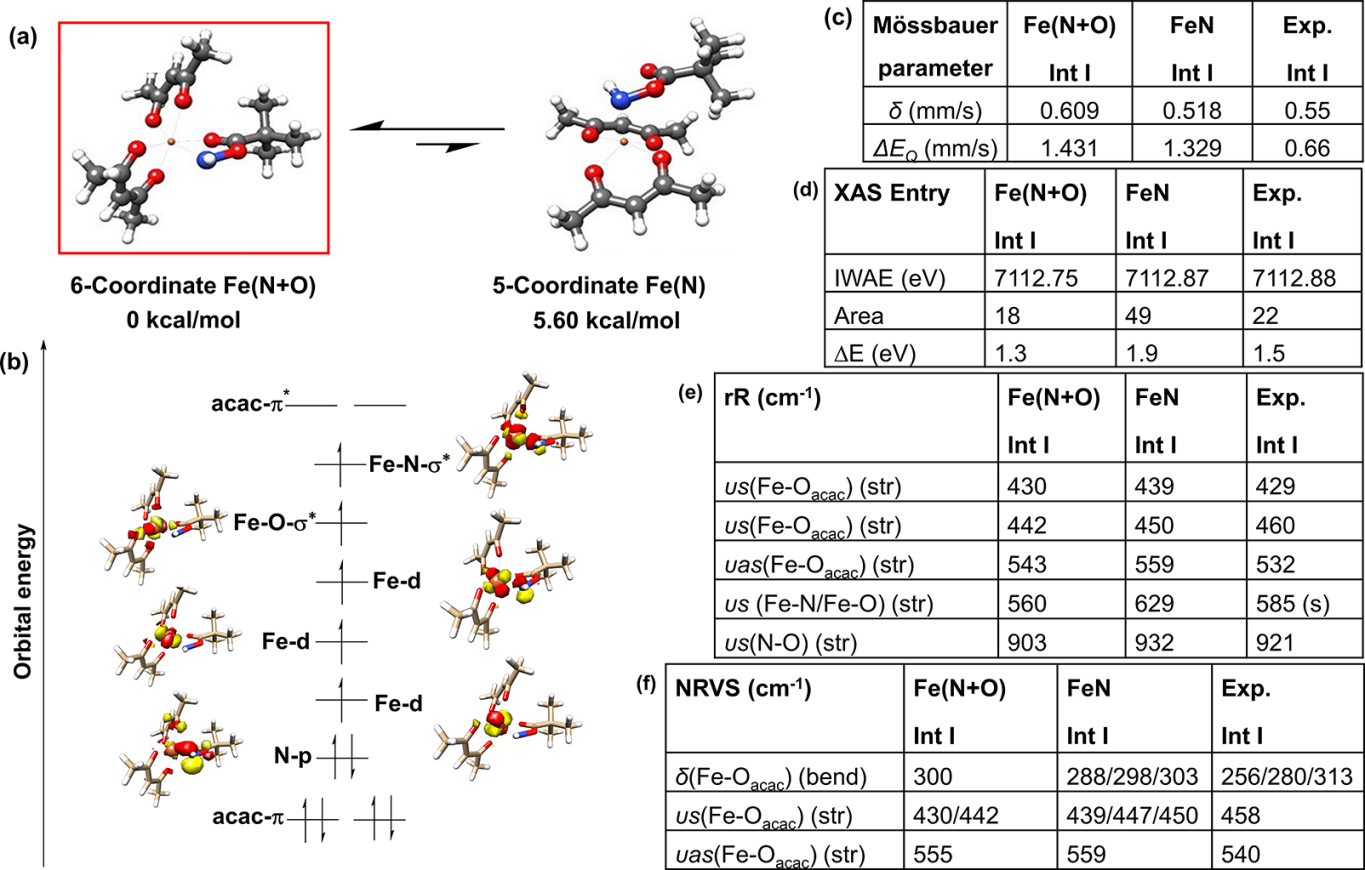
(a) Possible binding modes for **Int I**.: Fe(N+O) coordination and only FeN coordination. (b) Electronic structure for **Int I** (6-coordinate model) based on QROs. Calculations were carried out at the B3LYP-D3/def2-TZVP level. Correlation of experimental and calculated spectroscopic parameters of **Int I** for (c) Mössbauer, (d) HERFD-XAS, (e) rR (f) NRVS.

As discussed in the experimental section, addition of the aminating agent (PivONH_3_OTf) to a solution of the Fe(II) precursor **1** results in the formation of a wine-red species, **Int I**. For **Int I**, the experimental Mössbauer spectrum shows an isomer shift (δ) of 0.55 mm/s and a quadrupole splitting (*ΔE*_Q_) of 0.66 mm/s (Figure 4 middle). In addition, Fe K-edge HERFD-XAS shows a rising edge that is shifted to higher energy relative to the precursor **1** (Figure 5a). These observations are most consistent with **Int I** containing a high spin (*S*_t_ = 5/2) Fe(III) center. CW-EPR (Figure 3 and Figures S17–S19, SI) is also consistent with this assignment. From the calculated spin state energetics, for **Int I**, a noninteger spin system, the sextet ground state (*S*_t_ = 5/2; 0 kcal/mol) is favored in line with the experimental observations over the duplet (*S*_t_ = 1/2; 12.8 kcal/mol) or quartet spin state (*S*_t_ = 3/2; 10.4 kcal/mol) (Table S16, SI). Considering the geometric features of **Int I**, the calculations revealed two possibilities for the binding mode of the aminating reagent (PivONH_3_OTf) to the iron-acac scaffold as shown in Figure 6.

In the 6-coordinate octahedral model of **Int I** (Figure 6a), the aminating agent coordinates to the iron center both through the nitrogen and keto oxygen Fe(N+O), whereas in the alternative model higher energy conformer (5.6 kcal/mol) only iron–nitrogen binding Fe(N) occurs resulting in a 5-coordinate geometry (Figure 6a). For the 6-coordinate isomer of **Int I**, the calculated Fe–N bond distance is 2.00 Å, while the Fe–O_keto_ bond distance is 2.18 Å (Table S31, SI), indicating a highly distorted octahedron around the high spin Fe(III) systems.^141,142^

#### 3.2.2 Electronic Structure of **IntI**

**Int I** being a high-spin Fe(III) system has a half-filled d shell (d^5^ configuration), with the singly occupied metal d-based MOs having predominantly metal character. The electronic structure of the 6-coordinate Fe(N+O) model has been discussed here as a reference (Figure 6). From the electronic structure, it is evident that unlike precursor **1**, which shows evidence for MLCT transitions (Figure S33), **Int I** has filled ligand-based molecular orbitals (MOs) (acac π orbital and σ orbital of the aminating agent) that give rise to LMCT transitions from both the acac and aminating agent’s filled orbitals to half-filled metal d-based MOs (Figure 6b). Though the electron density of the ligand-based MOs is highly delocalized, the LMCT transition can be attributed mostly to the coligand aminating agent (PivONH_3_OTf).

#### 3.2.3 Correlation of Experimental and Computed Spectroscopic Parameters of **IntI**

##### Preferred Model for **IntI**

For **Int I**, it becomes evident that, the 6-coordinate distorted octahedral Fe(N+O) model is not only favored thermodynamically compared to the 5-coordinate Fe(N) model (5.6 kcal/mol higher), all experimental spectroscopy (UV–vis ABS, Mössbauer Spectroscopy, resonance Raman, NRVS) when correlated with the computational calculations, supports the 6-coordinate Fe(N+O) binding mode for **Int I** (Figure 6 and computational section of SI). The most convincing support of the 6-coordinate distorted O_h_ geometry for **Int I** is evident from the pre-edge HERFD-XAS results (Figure 5, and Figure S38, Table S20, SI) where both the pre-edge intensity and energy splitting Δ*E*, for the 6-coordinate Fe(N+O) model (pre-edge intensity = 18 and Δ*E* = 1.3) of **Int I**, matches well with the experimental result (experimental pre-edge intensity = 22 and Δ*E* = 1.5) further ruling out the FeN only binding mode of **Int I** (Figure 6; also see computational section of SI). The precursor **1** loses both water molecules, for a simultaneous coordination via nitrogen and keto-oxygen of the aminating agent (PivONH_3_OTf) to form **Int I** (*vide infra*). Thus, **Int I** is best described as a 6-coordinate distorted octahedral high spin Fe(III) (*S*_t_ = 5/2) species, with a nitrogen and keto-oxygen Fe(N+O) binding mode of the aminating agent and may be termed as an Fe(III)-*N*-acyloxy (**Int I**) intermediate species.

### 3.3 The Second Intermediate: **IntII**

#### 3.3.1 Spin State Energetics and Geometric Models of **IntII**

As emerges from the discussion above, **Int I**, a high spin Fe(III)-*N*-acyloxy species, is formed during the reaction of the Fe(II) precursor **1** with the aminating agent (PivONH_3_OTf). **Int I** converts to another new species designated as **Int II** (by loss of the *O*-pivaloyl group) having the composition [(acac)_2_FeNH] (Scheme 3, ESI-MS) with distinct experimental Mössbauer parameters compared to **Int I**, having an isomer shift (δ) of 0.57 mm/s and a quadrupole splitting (*ΔE*_Q_) of 0.40 mm/s (Figure 4 bottom). Fe HERFD-XAS for **Int II** exhibited a higher energy rising edge feature compared to precursor **1** and overlays with the rising edge of **Int I**, thus suggesting **Int II** could also be assigned to a high-spin Fe(III) species similar to **Int I** (Figure 5a). However, unlike **Int I**, CW-EPR in perpendicular mode revealed **Int II** to be an EPR silent integer spin species (Figure 3). The combination of these results leads to the intriguing question how the electronic structure of **Int II** may best be formulated? Given that the stoichiometry, mass and overall total spin-state of **Int II** are known, the possible formulations involve either a spin-coupled Fe(III)–NH^•^ metal-radical system or a high-valent Fe(IV)=NH species.

**Figure 7:**
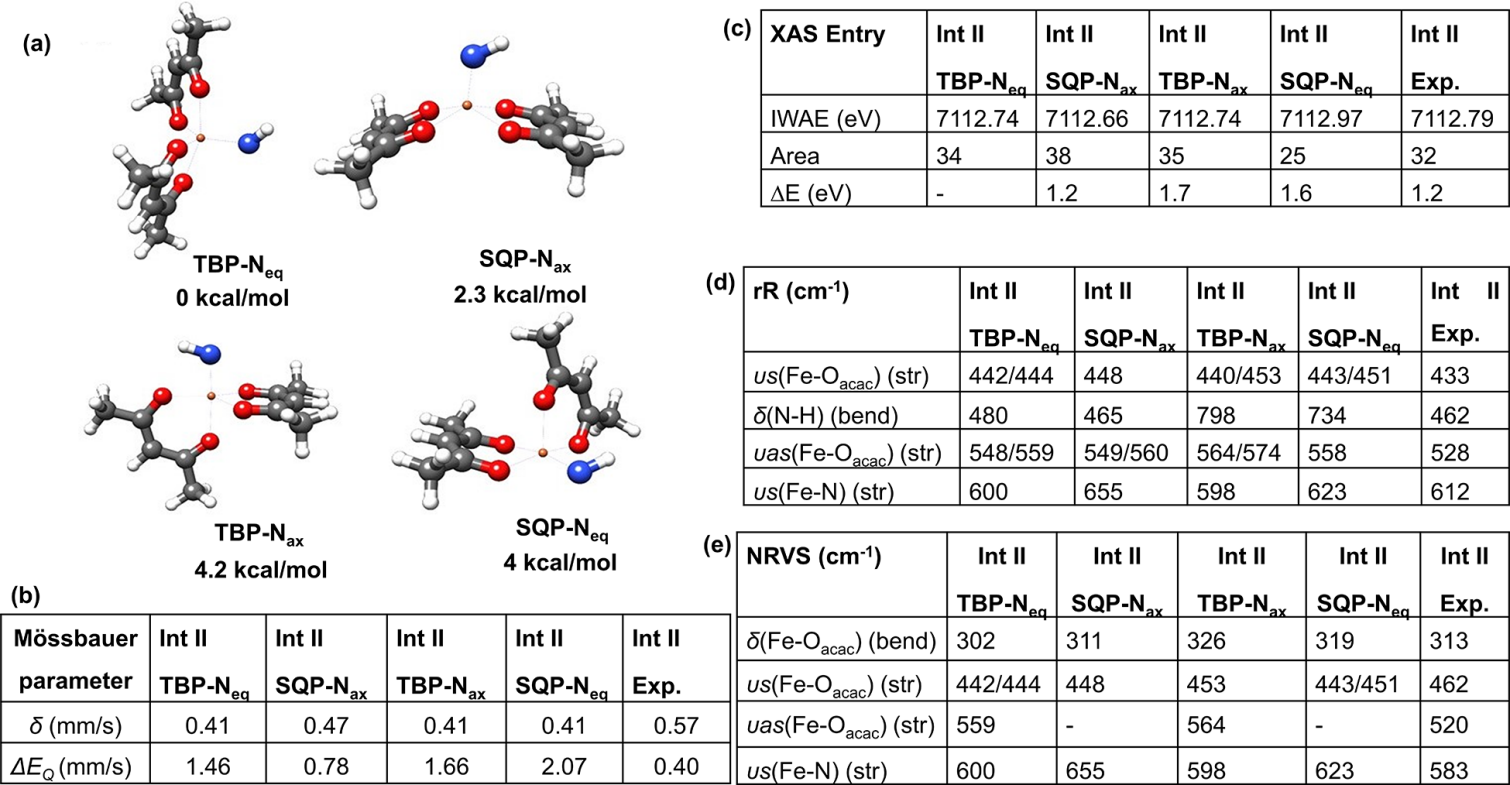
(a) Plausible geometric isomers for **Int II**.: Distorted trigonal-bipyramidal geometry with equatorial nitrogen (TBP-N_eq_), Square-pyramidal geometry with axial nitrogen (SQP-N_ax_), Trigonal-bipyramidal geometry with axial nitrogen (TBP-N_ax_) and Square-pyramidal geometry with equatorial nitrogen (SQP-N_eq_). Calculations were carried out at the B3LYP-D3/def2-TZVP level. Correlation of experimental and calculated spectroscopic parameters of **Int II** for (b) Mössbauer, (c) HERFD-XAS, (d) rR (e) NRVS.

In terms of energetics, in agreement with the experimental findings, the DFT energy calculations of various spin states clearly favor a quintet state (*S*_t_ = 2) as the electronic structure over the alternatives (*S*_t_ = 0, 1, 3; relative free energy 13.9 kcal/mol, 8.1 and 4.1 kcal/mol respectively) (Table S21, SI). Considering the geometric features of **Int II**, calculations revealed four possible geometric conformers as shown in Figure 7a. From the free energy calculations, the distorted trigonal bipyramid conformer with an equatorial disposition of the nitrogen (TBP-N_eq_) is favored over the other possible conformers (TBP-N_ax_ = 4.2 kcal/mol, SQP-N_eq_ = 4 kcal/mol and SQP-N_ax_ = 2.3 kcal/mol) (Figure 7a). From the calculated bond distance parameters for the different conformers of **Int II** (*d*(Fe–N) = 1.75–1.85 Å), it is found that a significant decrease of the Fe–N bond length is found compared to **Int I** (*d*(Fe–N) = 2.00 Å) (Tables S31 and S32, SI). This reflects the intermediate character of the Fe–N binding mode in **Int II**, which is discussed in detail in the electronic structure discussion part of **Int II**. However, before delineating the electronic structure of **Int II**, we shed light on correlating the experimental observables (spectroscopic parameters) of **Int II** to computational calculations for a better overview of the spectroscopic properties, which in turn would help to portray the electronic structure and bonding more explicitly.

#### 3.3.2 Spectroscopic Correlation to Quantum Chemical Calculations for **Int II**

From the calculated Mössbauer spectrum of different conformers of **Int II**, the square-pyramidal isomer with the axial nitrogen SQP-N_ax_ (δ = 0.47 mm/s, *ΔE*_Q_ = 0.78 mm/s) gives a value relatively close to the experimental result (δ = 0.57 mm/s, *ΔE*_Q_ = 0.40 mm/s) compared to the other models in terms of the isomer shift^140,143−146^ (Figure 7b). A point to note is the quadrupole splitting is not considered for correlation as previous studies have shown that the predicted isomer shifts tend to be more reliable than the calculated quadrupole splittings.^140,144,146−148^ In all the model conformers of **Int II** there is a decrease in calculated isomer shift value compared to **Int I**. The computed resonance Raman spectra for the most stable conformers of **Int II** (see computational section of SI) agree well with the experimentally observed υs(Fe–O_acac_) and υas(Fe–O_acac_) stretches as well as the δ(H–N–Fe) bend with a small calculated ^15^N isotope shift in agreement with the experimental result (Figure S40, Table S24 and Table S25, SI). However, the distinguishing peak among the model conformers is the Fe–N stretch which occurs at around 600 cm^–1^ for TBP-N_eq_ and TBP-N_ax_ isomers, and shifts to higher energy at 623 cm^–1^ for the SQP-N_eq_ model and at 655 cm^–1^ for the SQP-N_ax_ conformer (Figure S40, Table S24, SI). In the experimental rR spectrum of **Int II** (Figure S26 and Figure 7d, Table S24 and Table S25, SI), the Fe–N stretch is observed at 612 cm^–1^ with small ^15^N isotope sensitivity, which agrees well with the trigonal bipyramidal geometry. Analogous to rR, the computed Fe–O_acac_ stretch in the NRVS of **Int II** is shifted to higher energy compared to models for **Int I** and precursor **1** and this trend matches well with the experimental NRVS (461 cm^–1^ for **Int II**, 458 cm^–1^ for **Int I** and 440 cm^–1^ for **1**, Figure S27, Table S9), with a small feature around 583 cm^–1^ for **Int II** in the experimental spectrum corresponding to the Fe-NH stretch (Figure S41, Table S26, SI). Though the rising edge energy of Kβ_1,3_ HERFD-XAS data is consistent with the assignment of a high spin Fe(III) oxidation state for **Int II** similar to **Int I**, in the experimental pre-edge region, we observed an ∼36% increase in intensity of pre-edge peaks for **Int II** compared to **Int I** and precursor **1**, both of which have distorted octahedral geometry (Figure 5b and Table 2). This is consistent with the calculated 5-coordinate iron center in **Int II** with a decrease in metal symmetry, thereby increasing the dipole contributions due to the 3d–4p mixing (see Figure 7 and computational section of SI). The increase of the pre-edge intensity for **Int II** compared to **Int I** is also consistent with a shortening of the Fe–N bond length for **Int II** (Table S31 and Table S32, SI). The experimentally observed energy splitting in the pre-edge region, Δ*E* for **Int II** (Δ*E* = 1.2 eV), matches exactly with the calculated energy split for the SQP-N_ax_ isomer (Figure S42, Table S27).

##### Preferred Model for **IntII**

From the correlation of the experimental spectroscopic parameters of **Int II**, with the calculated values of the different geometric conformers, it is difficult to select a preferred conformer as the only favorable model. Though Mössbauer and HERFD-XAS suggest a square-pyramidal geometric conformation with the axial disposition of the nitrogen (SQP-N_ax_), free energy calculation and vibrational spectroscopies favor the trigonal bipyramid geometry with an equatorial nitrogen atom (TBP-N_eq_) for **Int II**. Overall calculations agree with the experimental results, confirming that **Int II** has a 5-coordinate high spin Fe(III) center, with a shorter Fe–N bond compared to **Int I**.

#### 3.3.3 Electronic Structure of **IntII**

The interesting spectroscopic features for **Int II** prompted us to explore the electronic structure, which would also help to shed light on the unique Fe–N bonding interactions. Here, we considered TBP-N_eq_ and SQP-N_ax_ to be the most favorable conformers based on energy calculation as well as spectroscopic and computational data analyses. **Int II** was shown from EPR to be an integer spin system (Figure 3) with the quintet state (*S*_t_ = 2) calculated to be the most stable spin state. The Mössbauer (Figure 4 bottom) and HERFD-XAS rising edge feature (Figure 5a) predicts a locally high spin Fe(III) configuration at the iron atom. Interestingly, the NBO analysis for the energetically favorable conformer of **Int II** shows that a significant portion of the spin density resides at the nitrogen atom (entries 2 and 3, Table S28, SI). Taken together, the electronic structure of **Int II** (TBP-N_eq_ and SQP-N_ax_) can be described in terms of broken-symmetry DFT. The nitrogen radical (*S* = 1/2) couples antiferromagnetically with the high spin Fe(III) center (*S* = 5/2) to form an overall spin state of *S*_t_ = 2 for **Int II**. The coupling constant *J* is defined via^149,150^
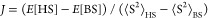
and comes to −524.41 cm^–1^ for the TBP-N_eq_ isomer and to −893.41 cm^–1^ for the SQP-N_ax_ isomer at the BS-DFT level. From the NBO analyses comparison for **Int I** and **Int II** (Table S28, SI), it can be seen that while the charge and spin density of the iron center is unaffected for **Int II** compared to **Int I**, the spin population on the nitrogen significantly decreases with a negative sign, which reflects the antiferromagnetic coupling between iron and nitrogen in **Int II**. The bond between the high spin iron and the nitrogen radical (iron-iminyl bond) can be understood in terms of one σ/σ* and one π/π* orbital pair (see SI for NBO analyses). It is to be noted that the different conformers of **Int II** (TBP-N_eq_, SQP-N_ax_, TBP-N_ax_ and SQP-N_eq_) show very different electronic structures at the DFT-level (Table S28, SI). In order to gain a better understanding of the electronic structure, CAS-SCF calculations with 12 electrons in 11 orbitals were performed on the electronic ground state. The starting orbitals are, however, the Quasi-Restricted Orbitals (QROs) obtained from the DFT calculation. Interestingly, different from DFT, CAS-SCF predicts very similar electronic structures and, therefore, atomic charges and spin populations for all isomers of **Int II** with sufficient negative spin population on the nitrogen (Table S29 for Löwdin spin and charge analysis).

**Figure 8:**
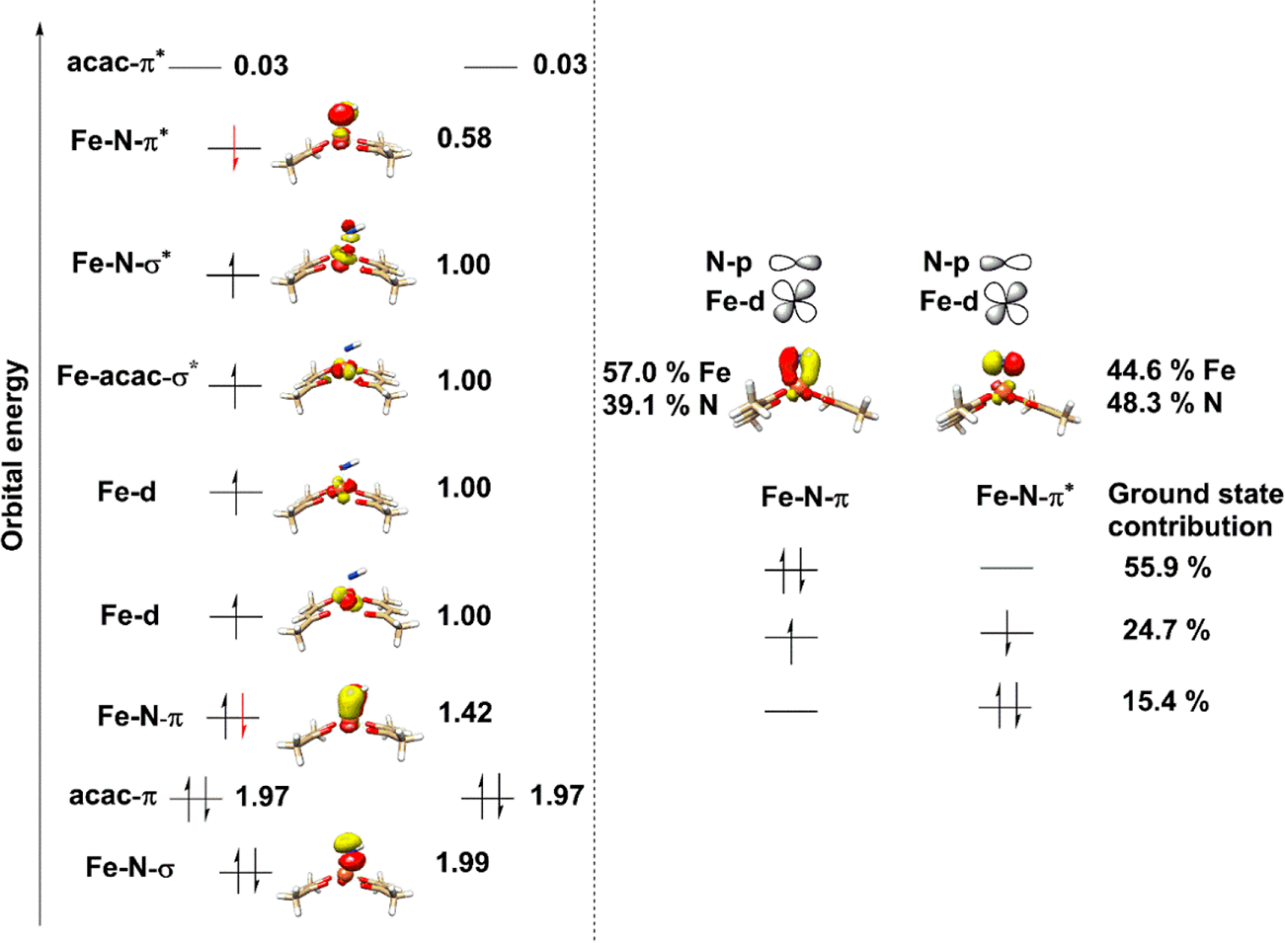
Electronic structure of **Int II** (SQP-N_ax_) (Left). Orbital representation with natural orbitals obtained from CAS(12,11). Natural occupation numbers are given in numbers of electrons. Right: States with largest contributions to the overall ground state.

The Fe–N binding situation for **Int II** can be described with one σ- and one π-bond. The σ-bonding orbital is doubly occupied and very low in energy, even below the ligand π-orbitals. The corresponding σ-antibonding orbital is the highest singly occupied orbital (Figure 8, left). As can be seen from Figure 8 (right), the ground state wave function has mainly contributions from three states reflecting the diradical coupling. The three states describe the distribution of the two electrons of the Fe–N π-bond to the corresponding orbitals. The π-orbital is more metal-based, while the π*-orbital is more nitrogen-based. The three states describing the antiferromagnetic diradical coupling are, therefore, responsible for the negative spin population predicted for the nitrogen atom.

The Mayer bond order^151^ at the CAS-SCF level is 0.97 for the distorted trigonal-bipyramidal isomer with equatorial nitrogen (TBP-N_eq_) and 0.99 for the quadratic-pyramidal isomer with an axial nitrogen atom (SQP-N_ax_), reflecting the net existence of one single Fe–N bond in **Int II**. From the calculated bond order, the binding mode of **Int II** can be best interpreted as an Fe(III) center coupling antiferromagnetically to an iminyl-radical. The local spin analysis^152^ at the CAS-SCF level supports this interpretation with an effective spin at the NH-subsystem of 0.57 (or 1.14 unpaired electrons) and 2.36 (or 4.72 unpaired electrons) at the residue for the SQP-N_ax_ conformer. For the TBP-N_eq_ the NH-subsystem has an effective spin of 0.58 (1.16 unpaired electrons) while the residue has a spin of 2.39 (4.78 unpaired electrons).

The bond order at the BS-DFT level is significantly larger (1.35 for the SQP-N_ax_ conformer), describing the partial double bond character of **Int II**. The partial double bond character at the BS-DFT level is also captured by the quite small spin population located at the nitrogen atom (Table S28, entry 3). In BS-DFT the strong coupling leads to a significant covalent bond character that blurs the oxidation state assignment between +3 and +4, with closer resemblance to the +3 oxidation state. This is also in agreement with the calculated Mössbauer isomer shifts of **Int II**, which are systematically smaller by ∼0.1 mm/s in comparison with **Int I**, with a decrease of the calculated Fe–N bond length of **Int II** (∼1.75–1.85 Å) with respect to **Int I** (∼2 Å) (see Table S31 and Table S32, SI for calculated bond distances). Thus, from electronic structure analyses, it seems that the very strong coupling between iron and the nitrogen radical is partially in the way of an unambiguous oxidation state assignment for **Int II**, but the favored way of looking at it is the high-spin Fe(III) center (*S* = 5/2) is antiferromagnetically coupled to NH^•^ radical species (*S* = 1/2) in **Int II** to form an overall *S*_t_ = 2 integer spin species.

A point to note here about Fe–N bonding for **Int II** is the unusually long Fe–N bond length (calculated at ∼1.75–1.85 Å) compared to previously reported terminal iron imido complexes (e.g., [PhBP_3_]Fe(N-tol) 1.658(2) Å, *S*_t_ = 1/2;^63^ (^Me^nacnac)Fe(NAd) 1.662(2) Å, *S*_t_ = 3/2;^67^ (^iPr^PDI)Fe(NAr) 1.705–1.717 Å, *S*_t_ = 1;^64^ [(N4Py)Fe(NTs)]^2+^ 1.73 Å, *S*_t_ = 1^79^). However, Betley and co-workers have structurally characterized iron imido/iminyl complexes supported by bulky ligands ([ArL]FeCl[N(*p*-^*t*^BuC_6_H_4_)] 1.768 (2) Å, *S*_t_ = 2)^62,72^ where a high-spin Fe(III) center antiferromagnetically coupled to an imido/iminyl-based radical (*J* = −673 cm^–1^) resembles the calculated Fe–N bond length found for **Int II** (Table S32, SI), supporting our assignment of the Fe–N bond character in **Int II**.

**Scheme 4:**
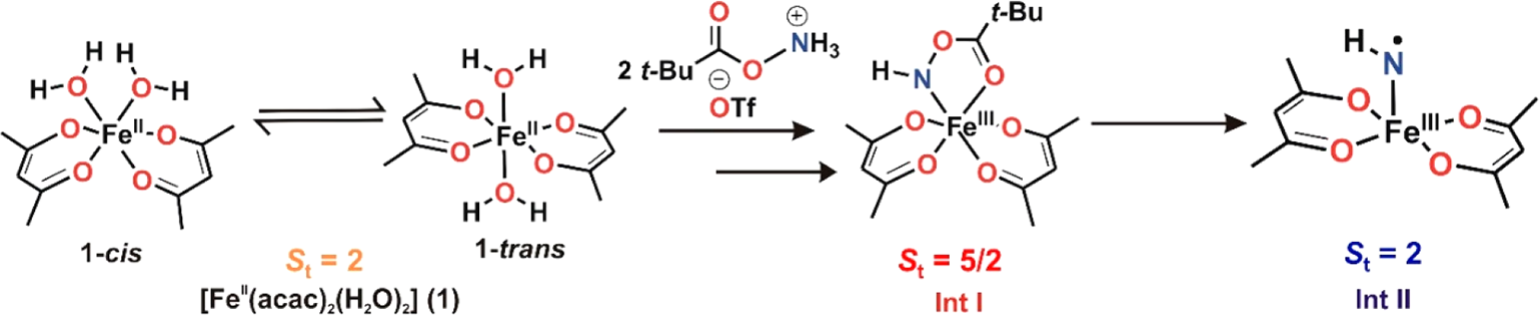
Proposed Iron–Nitrogen Intermediates Involved in the Reaction of **1** and PivONH_3_OTf (*vide infra*)

Thus, combined together, the spectroscopic study and computational calculations support that a mixture of the *cis*- and *trans*-isomers of Fe(acac)_2_(H_2_O)_2_ (**1**), a high spin (*S*_t_ = 2) Fe(II) complex, reacts with the hydroxyl amine derived N–O reagent (PivONH_3_OTf) to form a putative high spin (*S*_t_ = 5/2) Fe(III) species referred to as **Int I** (Scheme 4). The two water molecules of the precursor complex **1** are replaced by a bidentate [Fe(N+O)] coordination motif of the aminating agent (PivONH_3_OTf) to generate **Int I**, a high spin Fe^III^-*N*-acyloxy species. **Int I** eventually converts to **Int II**, a spin integer species (*S*_t_ = 2), with an HN· radical (*S* = 1/2) antiferromagnetically coupled to an Fe(III) (*S* = 5/2) center to form an Fe(III)-iminyl radical species as the catalytically active *N*-transfer agent (Scheme 4).

After elucidating the electronic and geometric features of **Int I** and **Int II** by spectroscopic and computational study, the next question that needs to be addressed is how do these intermediates play a role in the aminofunctionalization reaction? The following section delineates the proposed reaction pathway by the iron–nitrogen intermediates (**Int I** and **Int II**) generated from the reaction of **1** and hydroxyl amine derived N–O reagent, for aminofunctionalization of styrenyl olefins.

## 4 Implications for the Reaction Mechanism

### 4.1 Catalyst Activation Pathway by the Hydroxylamine Derived Reagent: N–O Bond Cleavage Mechanism

In the iron–oxygen paradigm, iron-alkyl/acylperoxo intermediates and high valent iron-oxo intermediates have been rigorously studied by spectroscopic techniques and computational calculations, in both the enzymatic and synthetic model complexes, for oxygen atom transfer and HAT reactions.^37,153^ However, as discussed in an earlier section of the manuscript, analogous iron–nitrogen intermediates involved in *N*-transfer reactions remain somewhat underdeveloped, particularly the mechanistic details and the interplay in effecting chemo- and regioselectivity remains poorly understood for such systems.

**Scheme 5:**
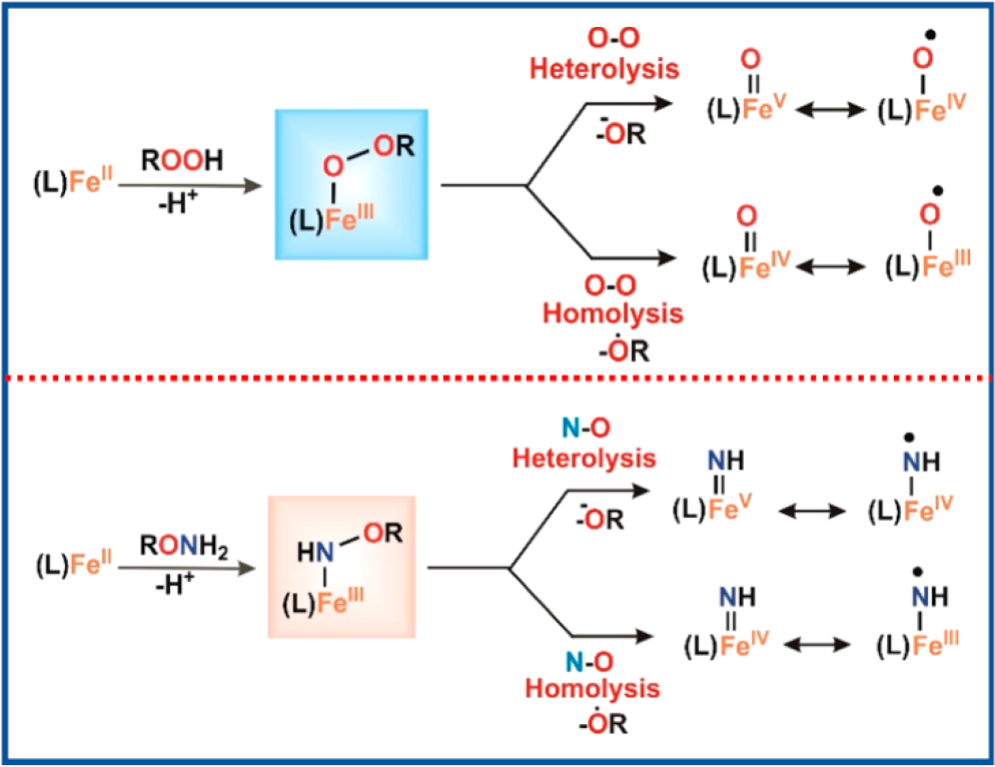
Proposed Pathway of O–O Bond Cleavage (Top) and Analogous N–O Bond Cleavage Presented in This Work (Bottom)

As is well established in the literature, reaction of alkyl/acylperoxide (ROOH) with Fe(II) complexes result in the formation of Fe^III^-alkyl/acylperoxo species, where the initial step involves conversion of the Fe(II) complex to an Fe(III) complex, presumably Fe^III^–OH, via oxidation with 0.5 equiv of ROOH. The next step is then followed by displacement of hydroxide by ROOH to give Fe^III^–OOR.^98,154,155^ Depending on the supporting ligand and spin state of Fe(III) (high spin/low spin), the Fe^III^–OOR species may undergo a heterolytic or homolytic O–O bond cleavage to generate high valent iron oxo intermediates for substrate oxidation (Scheme 5).^104,156,157^

In analogy with the alkyl/acyl peroxide (ROOH) chemistry, the hydroxylamine derived reagent PivO-NH_2_·HOTf (RONH_2_·HOTf, where R = pivaloyl group) acts as a dual oxidant and amino group donor for the iron catalyzed aminofunctionalization reaction. In the first step, the precursor Fe(II) complex (**1**) is oxidized by the aminating agent (PivONH_3_OTf) to form an Fe(III) complex, and in the second step, a second equivalent of the hydroxylamine derived reagent acts as a coligand to form a high spin Fe(III)-NHOR (**Int I**) type species, as evident from the combination of several analytical and spectroscopic techniques (UV–vis absorption spectroscopy, kinetic analyses, ESI-MS, GC, EPR, Mössbauer, HERFD-XAS, rR, NRVS) and computational studies. **Int I** resembles Fe^III^–OOR and may be classified as an Fe^III^-*N*-acyloxy species [Fe(III)-NH-OR] similar to the alkyl/acylperoxo analogue. The UV–vis chromophore of **Int I** at 480 nm (ε = 1156 M^–1^ cm^–1^) (Figure 1) can be assigned to an *N*-acyloxy to Fe(III) charge transfer transition akin to alkyl/acyl peroxo to Fe(III) LMCT.^104,158^ The dual role of PivONH_3_OTf as oxidant and amino source and the above-discussed activation pathway is also consistent with the observation that always greater than 1 equiv (∼2 equiv) of PivONH_3_OTf reagent is required for formation of **Int I** from the precursor **1** complex.

**Int I**, an iron-*N*-peroxo analogue, likely undergoes N–O bond lysis to generate **Int II**, the active species responsible for *N*-transfer reactivity. Betley and co-workers have reported high-spin Fe(II)-nitroxido species which are stable in the absence of weak C–H bonds, but decay via N–O bond homolysis undergoing C–H activation.^159^ So far from our experimental results, spectroscopic analyses and theoretical calculations, it is evident that **Int I** converts to **Int II**, an Fe(III)-NH^•^ radical species [high-spin Fe(III)-iminyl radical species]. **Int II** subsequently participates in the *N*-transfer reaction; the detailed mechanism of aminomethoxylation of styrenyl olefin has been delineated in the subsequent section via DFT calculations (*vide infra*). Similar to metal–alkyl/acyl peroxide chemistry,^101,160−162^ product analyses from PivONH_3_OTf may be used as an indirect mechanistic probe, to differentiate between the proposed homolytic and heterolytic N–O bond cleavage pathways. In fact, GC-MS analysis of the headspace of the reaction after decay of **Int II** reveals formation of CO_2_ and isobutene (Scheme S1, Figure S15 and S16, SI). This further supports the homolytic cleavage pathway of the N–O bond of **Int I** (Scheme 5). The nitrogen and keto oxygen coordination mode of **Int I** is likely to be a factor that favors the N–O bond homolysis.

### 4.2 Proposed Reaction Profile

Kinetic measurements of complex **1** and aminating agent (PivONH_3_OTf) by stopped flow UV–vis measurements enabled us to extract the rate constants and order of the reaction. From the kinetic profile, it is evident that interaction of aminating agent PivONH_3_OTf with complex **1** has a first-order dependence on the concentration of aminating agent and a half-order dependence on the concentration of iron (Figure S7, SI). As such, the overall fractional order of the reaction suggests a complex multistep reaction. Herein, we postulate the activation pathway of the Fe(II) catalyst **1** by the hydroxyl amine derived N–O reagent (PivONH_3_OTf). The following two-step process was proposed for the formation of **Int I**, experimentally characterized as [Fe^III^(NHCOO*t*Bu)], where the hydroxyl amine derived aminating agent (*t*BuCOONH_3_OTf, *t*BuCO = Piv) acted as an oxidant in the first step and as a coordinating ligand in the second step to generate **Int I**:

**Step 1** Oxidation of the precursor **1**:
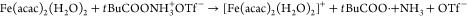
**Step 2***t*BuCOONH_3_^+^ coordination:
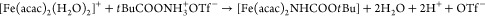
**Step 1 + Step 2**
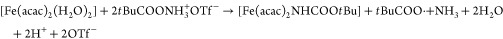
Thus, in the proposed activation pathway, the theoretical ratio of precursor **1** to the aminating reagent (PivONH_3_OTf) for the formation of **Int I** is 1:2; i.e., each equivalent of precursor **1** needs 2 equiv of aminating agent for formation of **Int I**, consistent with the experimental findings (0.55:0.9) (Figure S7, SI). The overall fractional order further supports the multistep pathway of catalyst activation to generate **Int I**.

**Figure 9:**
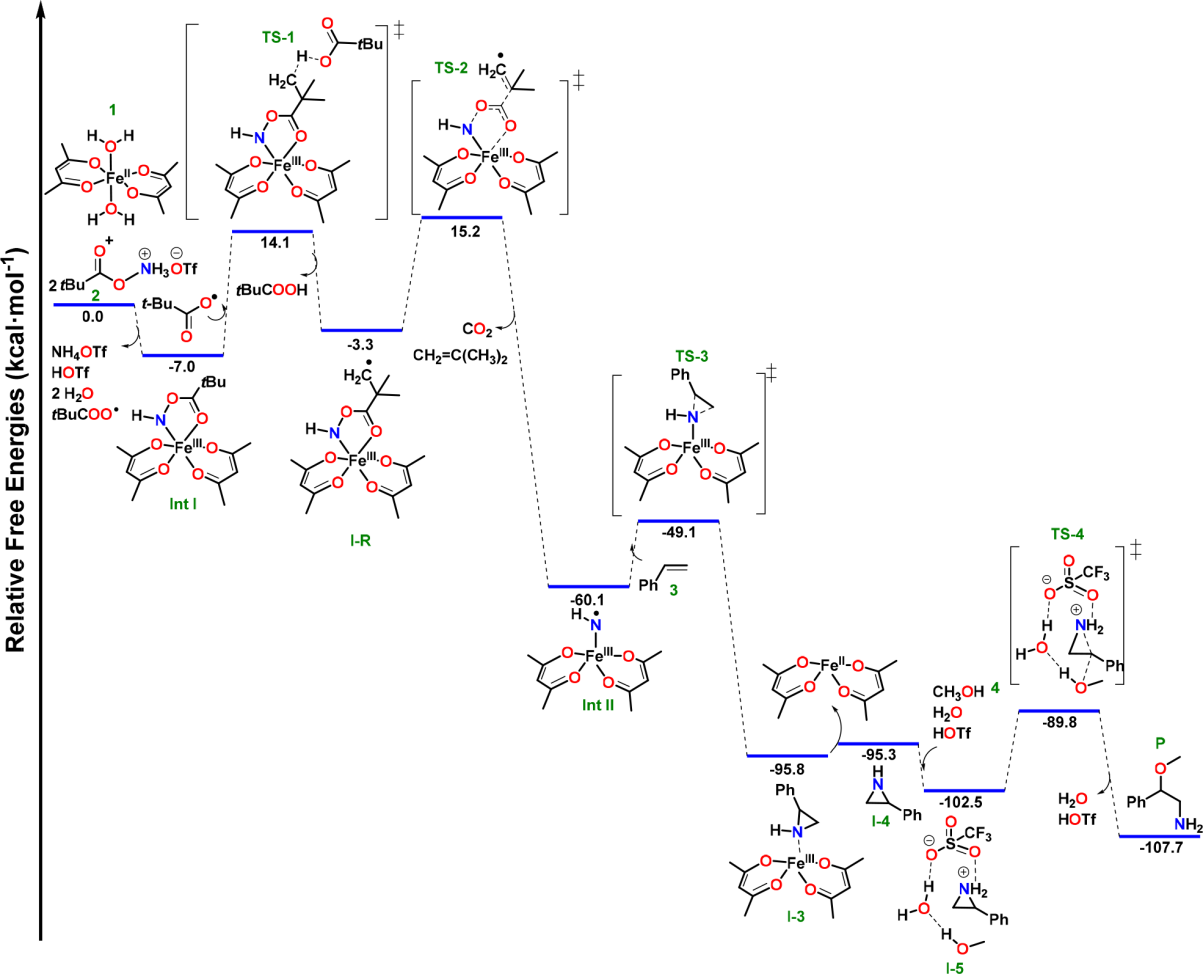
Calculated reaction pathway for the iron catalyzed regioselective aminomethoxylation of styrene by complex **1** and PivONH_3_OTf at the B3LYP-D3/def2-TZVP level.

The final reaction equation leading to the formation of **Int I** can, thus, be summarized as
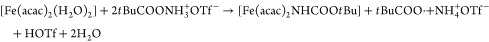
The corresponding free reaction energy is −7.0 kcal/mol (Figure 9). Thus, the proposed activation reactions agree with the experimental findings and suggest that the activation process should be exergonic due to the formation of stable ion–ion interactions and stable molecules like water. The calculated reaction pathway for the iron catalyzed regioselective aminomethoxylation of styrene by complex **1** and PivONH_3_OTf is presented in Figure 9, as one of the most plausible pathway based on the experimental results.

The proposed reaction pathway consists of multiple reaction intermediates (Figure 9). **Int I**, a high spin Fe(III)-*N*-acyloxy (*S*_t_ = 5/2) species, and **Int II**, a high-spin Fe(III)-iminyl radical (Fe^III^–NH^•^) (*S*_t_ = 2), were detected and characterized using spectroscopic methods aided by computational study. The decomposition of **Int I** follows a two-step pathway. The *t*BuCOO^•^ radical, which is generated during the activation of Fe(II) catalyst **1** by the hydroxyl amine derived N–O reagent (PivONH_3_OTf) to form **Int I** (Figure 9), abstracts a hydrogen atom from **Int I** leading to the formation of a transient radical intermediate **I-R**. This hydrogen abstraction process lowers the barrier for the homolytic cleavage of the N–O bond of **Int I** substantially (for the alternate higher energy pathways; see Table S34, SI). **TS-2** describes the decarboxylation process of **I-R** yielding CO_2_ and isobutene (detected experimentally) and subsequently forming **Int II**. Decarboxylation occurs via a concerted mechanism, in which three covalent bonds are simultaneously broken: (i) the Fe–O bond involving the keto group of the PivONH ligand, (ii) the N–O bond and (iii) the C–C bond. The competing stepwise pathway, in which the Fe–O bond is initially cleaved, is disfavored by ca. 10 kcal/mol as detailed in the SI. Importantly, the formation of **Int II** is highly exergonic, due to the concomitant formation of CO_2_ and isobutene. **Int II** eventually reacts with styrene to undergo an *N*-transfer reaction to form the putative Fe-aziridine adduct (**I-3**) via **TS-3**. The dissociation energy of **I-3** is very small, thereby, regenerating the catalyst for the next cycle. The aziridine is protonated under the acidic reaction condition (triflic acid from the reagent PivONH_2_·HOTf) to form **I-5**. The subsequent attack of the methanol molecule happens easily, reflected by the low reaction barrier (**TS-4**), to regioselectively form 2-methoxy-2-phenylethan-1-amine. A point to note here, considering the protonated form of the reagent (PivONH_3_OTf), formation of a protonated version of **Int I**, *i.e*. [**Int I-H**]^**+**^ [Fe(acac)_2_NH_2_COO*t*Bu]^+^, could be possible. Computational modeling suggests that **Int I** and [**Int I-H**]^**+**^ feature very similar spectroscopic properties, as detailed in the SI. However, the reaction pathway considering [**Int I-H**]^**+**^ as a first putative intermediate shows much higher energy barriers, and hence it is only reported in the SI (Figures S53–S56 and Tables S35–S39).

**Figure 10:**
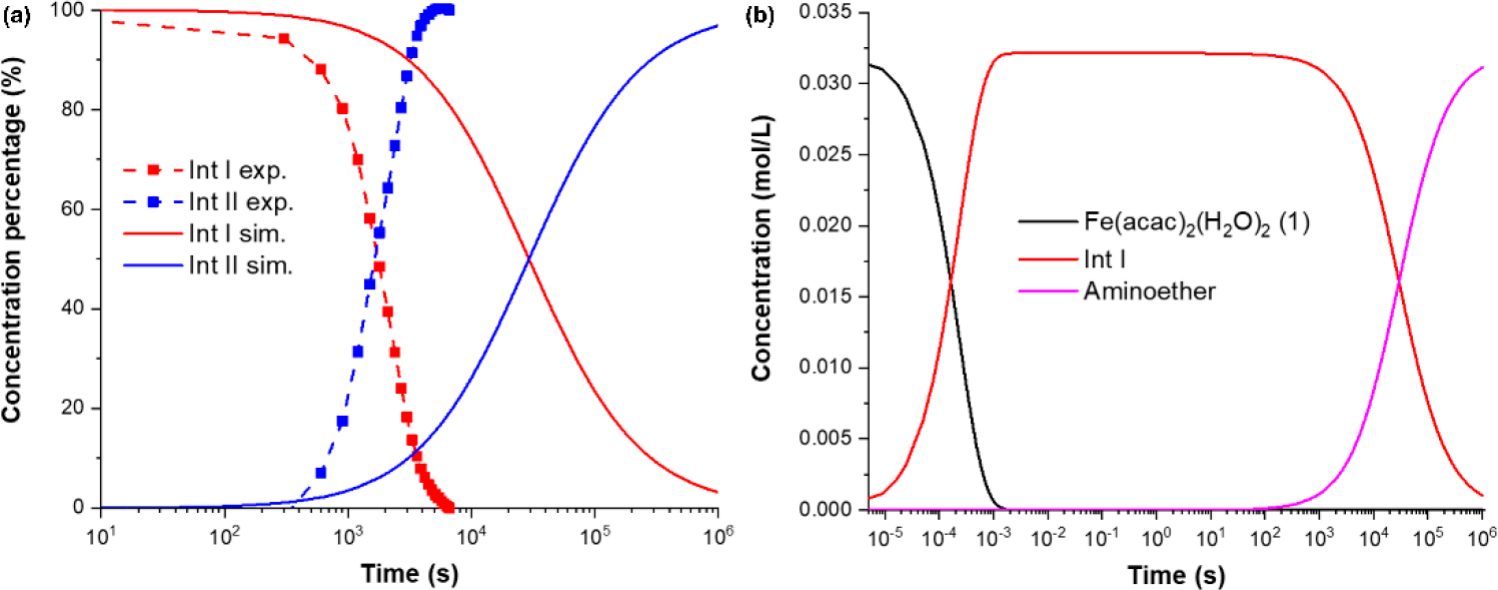
(a) Time-dependence of concentration percentages of **Int I** and **Int II**, obtained from kinetic simulations in the absence of styrene. Dashed lines indicate the experimental concentration profiles, with squares indicating the experimental data points (Left). Experimental percentages were obtained from the corresponding absorption spectra. (b) Concentration profiles of the precursor, reaction intermediates and product obtained from kinetic simulations in the presence of styrene (Right). **Int I** is the only intermediate that accumulates noticeably in the presence of styrene.

To verify the plausibility of the computed mechanism, kinetic simulations on the basis of the calculated reaction rates were carried out to estimate the time-dependence of the concentration of the key reaction intermediates. The comparison between the computed and experimental concentration profiles obtained in the absence of styrene is shown in Figure 10 (see the computational section of the SI for additional information, Figures S43–S49). Importantly, no noticeable accumulation of **Int II** was obtained from the kinetic simulations in the presence of styrene, which is consistent with the experimental findings (see also Figures S47 and S49, SI for details).

Figure 10 shows a qualitative agreement between the computed and the experimental concentration profiles. The simulations are consistent with the buildup of **Int I** and **Int II** and also reveal that the calculated time course of their buildup and decay is at least reasonable when compared to the experiment. Note that small changes in the reaction barriers cause large changes in the rate constants (e.g., a change in a barrier of 2 kcal/mol changes the rate constant by a factor of more than 30). Thus, the relatively small deviation between theory and experiment is consistent with the expected error associated with our computational methodology (e.g., originating from the approximate exchange-correlation functional employed).

One interesting point to note in this study is the facile *N*-group transfer reactivity of **Int II** to the styrenyl olefins via the transition state (**TS-3**) to form the putative Fe-aziridine adduct (**I-3**) (Figure 9). As discussed in the earlier part of the manuscript, many of the reported metal–nitrogen intermediates were found incompetent for *N*-group transfer reactivity due to the strong metal–nitrogen multiple bonds. In our study, the active *N*-transfer reagent, **Int II** has an interesting electronic structure and Fe–N bonding interaction. Our comprehensive spectroscopic and computational study has established that **Int II** is a high-spin Fe^III^-iminyl radical complex, where the nitrogen radical (*S* = 1/2) couples antiferromagnetically with the high-spin Fe(III) (*S* = 5/2) center to form an integer spin (*S*_t_ = 2) species (*J* = −524.41 cm^–1^), with an unusually long Fe–N bond (1.75–1.85 Å), and insignificant Fe–N multiple bond character. The high spin character of the Fe(III) center (*S* = 5/2) and the radical character on nitrogen (*S* = 1/2) probably weakens the Fe–N bond, which facilitates the *N*-group transfer reactivity, thereby, exhibiting enhanced *N*-transfer catalytic reactivity by **Int II**. Similar *N*-transfer reactions by structurally characterized high spin iron-imido/iminyl complexes supported by bulky ligands have also been reported in literature and in fact, the corresponding iminyl species exhibited enhanced rates compared to the imido congener toward C–H amination.^62,72,163^ Furthermore, it has been reported that the ability of the imido or iminyl components to delocalize spin density through the substituent on nitrogen (aryl vs alkyl) results in a greater barrier toward functional group transfer.^163^ In contrast, **Int II**, an Fe(III)-iminyl radical species [(acac)_2_Fe(III)-NH^•^] reported in this work as the active *N*-transfer agent, lacks any *N*-substitution to stabilize the electron spin density on the metal–nitrogen vector, thus, being ideally poised for transferring the *N*-functionality. Hence, the unique electronic structure of the Fe(III)-iminyl radical species (**Int II**) correlates to the *N*-transfer catalytic reactivity. It is of interest to note that the reactive species reported herein is distinct from the typical high-valent Fe(IV) or Fe(V) assignment invoked in Fe-mediated group transfer catalysis.^37,79,80,85,86,164^ This work highlights the potential of utilizing metal coordinated nitrogen-centered radicals^50,54^ as a standard strategy in chemical synthesis and catalysis. Future research in our laboratories will focus on new reaction designs for structurally isolating the “atypical” reactive intermediates (Fe(III)-*N*-acyloxy and Fe(III)-iminyl radical) explored in this study and harnessing their reactivity toward various substrates.

## 5 Conclusion

**Scheme 6:**
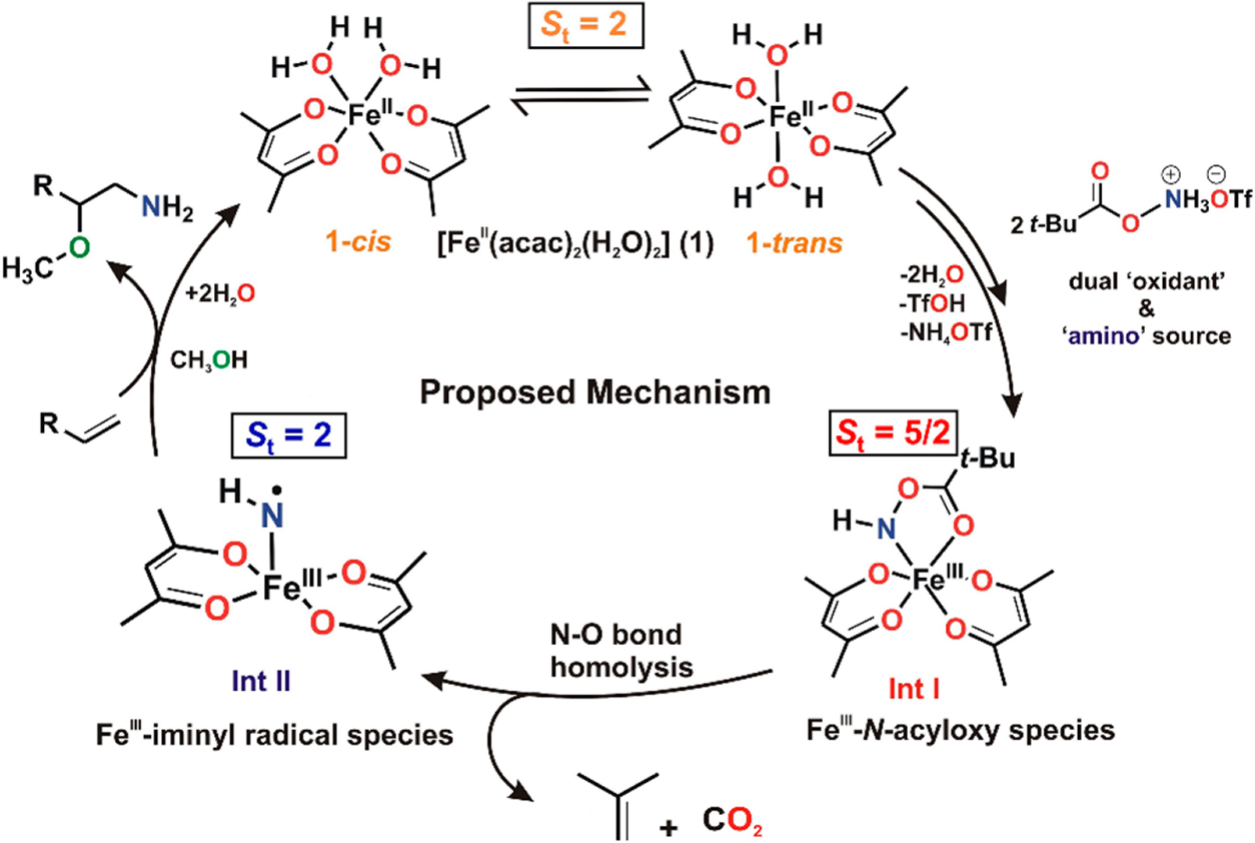
Proposed Mechanism for Reaction of **1** with PivONH_3_OTf To Generate Iron–Nitrogen Intermediates Involved in Regioselective Aminomethoxylation of Alkenes

A wide range of analytical and spectroscopic techniques (UV–vis absorption spectroscopy, kinetic analysis, ESI-MS, GC-MS, ^1^H NMR, EPR, Mössbauer spectroscopy, HERFD-XAS, resonance Raman spectroscopy, nuclear resonance vibrational spectroscopy) were used in this work to understand the geometric and electronic structure of the reaction components and the mechanism of a synthetically relevant iron catalyzed aminofunctionalization reaction of olefins–specifically, the aminomethoxylation of styrene.^21^ The results obtained from the experimental techniques were correlated with computational protocols to enable a clear understanding of the catalytic reaction mechanism and the contribution of the reactive intermediates to *N*-group transfer activity. From the spectroscopic and computational study, it was shown (Scheme 6) that a high spin Fe(II) (*S*_t_ = 2) catalyst [Fe(acac)_2_(H_2_O)_2_] (**1**) reacted with a hydroxyl amine derived triflic acid salt (PivONH_3_OTf), which acted as a dual oxidant and a nitrogen source, to generate a wine red species referred to as **Int I**. ESI-MS, EPR, Mössbauer, HERFD-XAS, rR and NRVS experimental techniques, when correlated with computational calculations, revealed **Int I** to be a high spin Fe^III^(acac)_2_-*N*-acyloxy (*S*_t_ = 5/2) species with a distorted O_h_ geometry and a bidentate coordination motif of the aminating agent (PivONH_3_OTf) via nitrogen and keto oxygen to the iron-acac scaffold (Scheme 6). This reactive **Int I** [Fe^III^(acac)_2_-NH-OPiv] underwent N–O bond homolysis to generate an EPR silent, integer-spin species referred to as **Int II** [Fe(acac)_2_NH]. However, Mössbauer and HERFD-XAS measurements suggested **Int II** to be a high-spin Fe(III) (*S* = 5/2) species. Interestingly, the NBO analysis and CAS-SCF calculations on **Int II** showed that a significant portion of the spin density resides on the nitrogen atom. When spectroscopic results and calculations were combined, the electronic structure of **Int II** can be best described as a high-spin Fe(III) iminyl radical species [(acac)_2_Fe-NH^•^]. The nitrogen radical (*S* = 1/2) couples antiferromagnetically (*J* = −524.41 cm^–1^) with the high-spin Fe(III) center (*S* = 5/2) to form an integer spin state (*S*_t_ = 2) for **Int II**. The unusual electronic structure of **Int II**, with an elongated Fe–N bond, makes it highly efficient for participating in the *N*-transfer reaction to styrenyl olefins (Scheme 6, Figure 9), and in the presence of nucleophilic solvent, regioselectively forms aminoethers, which are versatile intermediates for the synthesis of bioactive compounds. Besides unravelling the mechanism for the aminomethoxylation reaction, the mechanistic cycle proposed above, and the electronic structure of the two new iron–nitrogen intermediates reported in this study, should also provide key reference points for understanding the mechanism of other iron catalyzed aminofunctionalization reactions of organic molecules by the hydroxyl amine derived reagent (PivONH_3_OTf) reported in literature.^22−28^**Int I**, a high spin Fe(III)-*N*-acyloxy species, has a resemblance to high spin Fe(III)-alkyl/acyl peroxo intermediates known in the literature, formed during the reaction of an Fe(II) catalyst and alkyl/aryl peroxides. Though Fe(NH) complexes as isoelectronic surrogates to Fe(O) functionalities are reported in literature,^79,80^ to the best of our knowledge this is the first report of a high-spin Fe(III)-*N*-acyloxy intermediate as a synthetic analogue of the Fe(III)-alkyl/acyl peroxo intermediate. Similar to an O–O bond cleavage mechanism to form active iron–oxygen intermediates in the realm of oxygenation/hydroxylation chemistry,^104,156,157^ this N–O bond cleavage mechanism is expected to open new avenues in the field of *N*-transfer reactions to organic molecules. The interesting electronic structure of the iron-iminyl radical intermediate (**Int II**) [(acac)_2_Fe-NH^•^] (*S*_t_ = 2), with a high spin Fe(III) (*S* = 5/2) center coupled to a nitrogen centered radical (*S* = 1/2) having an elongated Fe–N bond, facilitates efficient *N*-group transfer activity, circumventing the need to generate high-valent iron intermediates for group transfer reactivity.^37^ The insights obtained in this work, regarding the electronic structures and reaction mechanism from a combination of experiment and theoretical studies, are expected to help in the design of new, improved catalysts and reagents for amination reaction, as well as for broader aspects of group transfer chemistry. We hope the present study will have wider implications in correlating the field of catalysis and reaction design to spectroscopy and theory.

## Supporting Information Available

Experimental details, analytical and spectroscopic data, and computational coordinates (PDF)
