# Supplementary material for: A Combined Spectroscopic and Computational Study on the Mechanism of Iron-Catalyzed Aminofunctionalization of Olefins Using Hydroxylamine Derived N–O Reagent as the “Amino” Source and “Oxidant”
Source: J Am Chem Soc. 2022 Feb 4;144(6):2637–56. doi: 10.1021/jacs.1c11083 (PMC8855425; doi:10.1021/jacs.1c11083)
Supplement: Supplementary file 1 — ja1c11083_si_001.pdf [file ja1c11083_si_001.pdf]

Supporting Information

A Combined Spectroscopic and Computational Study on the Mechanism of Iron-catalyzed Aminofunctionalization of Olefins Using Hydroxylamine Derived N-O Reagent as the "Amino" Source and "Oxidant"

Sayanti Chatterjee,[a][d]* Ingolf Harden, [b] Giovanni Bistoni, [b] Rebeca G. Castillo,[a] Sonia Chabbra,[a] Maurice van Gastel, [b] Alexander Schnegg, [a] Eckhard Bill, [a] James A. Birrell,[a] Bill Morandi, [c][d] Frank Neese,[b]* and Serena DeBeer[a]*

[a] Max Planck Institute for Chemical Energy Conversion, Stiftstrasse 34–36, 45470 Mülheim an der Ruhr, Germany

[b] Max-Planck-Institut für Kohlenforschung, Kaiser-Wilhelm-Platz 1, 45470 Mülheim an der Ruhr, Germany

[c] ETH Zürich,Vladimir-Prelog-Weg 3, HCI, 8093 Zürich, Switzerland

[d] Max-Planck-Institut für Kohlenforschung, Kaiser-Wilhelm-Platz 1, 45470 Mülheim an der Ruhr, Germany (Previous affiliation)

*E-mail: serena.debeer@cec.mpg.de
frank.neese@kofo.mpg.de
sayanti.chatterjee@cec.mpg.de

Table of contents:

1) Abbreviations…………………………………………………………………………03

2) Experimental Section: Materials and Methods…………………………………………04

3) Synthesis of iron catalyst……………………………………………………………..07

4) Catalytic Reaction……………………………………………………………………08

5) Control Experiment…………………………………………………………………..09

6) UV-Vis Deconvolution………………………………………………………………09

7) Kinetic studies……………………………………………………………………….…11

8) Hammett Analyses……………………………………………………………………17

9) ESI-MS and GC-MS…………………………………………………………………...20

10) Electron Paramagnetic Resonance Spectroscopy……………………………………..29

11) Mössbauer Spectroscopy……………………………………………………………..33

12) Resonance Raman Spectroscopy……………………………………………………40

13) Nuclear Resonance Vibrational Spectroscopy…………………………………………40

14) Computational Section………………………………………………………………42

15) Multivariate Curve Analyses…………………………………………………………92

16) References……………………………………………………………………………..93

17) Cartesian Coordinates………………………………………………………………....96

Abbreviations

Piv – Pivaloyl / trimethylacetyl

*t*Bu – *tert*-Butyl

acac – acetylacetonate

OTf – trifluoromethanesulfonyl

Boc – *tert*-Butyloxycarbonyl

equiv. – equivalent(s)

EPR – Electron Paramagnetic Resonance

CW- Continuous wave

rR – Resonance Raman

NRVS – Nuclear Resonance Vibrational Spectroscopy

HERFD-XAS – High Energy Resolution Fluorescence Detected-X-ray Absorption Spectroscopy

ESI-MS – Electrospray ionisation mass spectrometry

NMR – Nuclear magnetic resonance spectroscopy

GC – Gas Chromatography

calc. – calculated

exp. – experimental

mT – milliTesla

eV – electron volt

μs – microsecond

Int – Intermediate

pm – picometre

ppm – parts per million

TBP – trigonal bipyramid

SQP – square pyramid

eq – equatorial

ax – axial

mM – millimolar

SH – Spin Hamiltonian

ZFS – Zero field splitting

DFT – Density functional theory

MCR -Multivariate Curve Resolution

SVD-Singular Value Decomposition

Experimental Section

Materials and Methods

All chemicals and reagents were obtained from commercial sources and were used without further purification unless otherwise noted. Solvents were used as purchased from commercial sources unless otherwise noted. The reagent $PivONH_3OTf$ was synthesized following a literature procedure.[1] All air-sensitive reactions were run under an atmosphere of argon using standard Schlenk and glove box techniques with flame-/oven-dried glassware unless stated otherwise.

Mass Spectrometry: Electrospray ionization (ESI) mass spectrometry was conducted on a Bruker ESQ 3000 spectrometer. High resolution mass spectrometry (HR-MS) was performed on a Finnigan MAT 95 (EI) and *Bruker* APEX II FTMS (7T magnet, ESI). The ionization method and mode of detection employed are indicated for the corresponding experiment and all masses are reported in atomic units per elementary charge (*m/z*) with an intensity normalized to the most intense peak.

Gas chromatography (GC) and Gas chromatography-Mass Spectrometry (GC-MS): Gas chromatography measurements for gaseous compounds were run on an Agilent 7890B spectrometer with a polyethyleneglycol column (30m, HP-PLOT Q inner diameter = 320 µm, film thickness = 20.0 µm). The components analysed by GC were further analysed by Finningan SSQ 7000 Quadrupole MS. GC-MS System using electron impact (EI) and chemical inonization (CI) sources.

Nuclear Magnetic Resonance (NMR) spectroscopy: Nuclear Magnetic Resonance spectra were measured using a Bruker AV- or Bruker Neo-spectrometer. ^{1}H-NMR was reported as follows: chemical shift, multiplicity (s = singlet, d = doublet, t = triplet, q = quadruplet, m =multiplet and br = broad), coupling constant (*J* values) in Hz and integration. Chemical shifts (δ) were reported with respect to the corresponding solvent residual peak at 7.26 ppm for $CDCl_3$. Spectra were processed with MestReNova 11.0.2 and coupling constants are reported as observed.

UV-Vis Absorption Spectroscopy (UV-Vis ABS): Solution electronic spectra (single and time-dependent) were measured on an Agilent 8453 diode array spectrophotometer in the range of 200 to 1100 nm.

Stopped Flow UV-Vis Spectroscopy: Stopped flow measurements have been performed under anaerobic condition inside a COYBOX using an SFM 300 stopped flow unit (Biologic) and a TIDAS MMS diode array detector (J & M Analytik Ag)

Elemental Analysis: Analyses for C, H, N content of the compounds were analysed by Mikroanalytisches Labor Kolbe, Oberhausen, Germany.

Resonance Raman Spectroscopy (rR): Samples were measured as free-hanging frozen droplets on small loop of a glass pipette that was precooled to 100 K with a cold nitrogen gas stream provided by a Cryostream 600 unit. Build-up of ice on the sample was prevented by an isolating warm nitrogen gas stream around the cold stream, also providing anaerobic conditions. The samples were excited with Cobolt-Hübner DPSS lasers in an electronic absorption of the respective intermediates. The Raman light was collected under 180 degree backscattering using a protected-silver off-axis parabolic mirror (Thorlabs). The Raman light is collimated and dispersed using a Trivista 555 triple monochromator equipped with a deep-depleted back illuminated CCD detector (Princeton Instruments).

Mössbauer Spectroscopy: Mössbauer samples were prepared by synthesis of the precursor [$^{57}Fe(acac)_2(H_2O)_2$] (1) from $^{57}FeCl_2$. ^{57}Fe labelled precursor complex (2 mM) in toluene was incubated with the aminating ($PivONH_3OTf$) agent (3 equiv.) for 15 min (**Int I**) and 90 min (**Int II**) at room temperature. After incubation the samples were transferred to Mössbauer sample holders and frozen in liquid nitrogen. Mössbauer spectra were recorded on a conventional spectrometer with alternating constant acceleration cooled with an Oxford Instruments Variox cryostat, using a57Co/Rh (1.8 GBq) γ-source. Samples were measured at 80 K and 1.7 K with and without applied magnetic field. Isomer shifts are relative to iron metal at 300 K. Spectra were simulated and fitted using Lorentzian quadrupole doublets using in-house software mf developed by Dr. Eckhard Bill.

Electron Paramagnetic Resonance (EPR) instrumentation: X-band CW EPR spectra were obtained using an X-band Bruker Elexsys E500 EPR spectrometer equipped with a ER4116DM dual mode resonator and an ESR 900 He cryostat. The sample temperature was stabilised at 10 K using a He flow cryostat (Oxford Instruments). The EPR spectra were obtained using a microwave frequency of ~9.64 GHz, microwave power of 0.2 mW, 400 mT field sweep centred at 205 mT, a Lock-In modulation amplitude of 0.7 mT, a time constant of 40.96 ms, a sweep time of 84 s and a modulation frequency of 100 KHz.
W-band pulse EPR measurements were performed using a Bruker ELEXSYS E680 spectrometer equipped with a homebuilt W-band bridge and a cryogen free Cryogenic 6 T magnet with a variable temperature insert. Electron spin echo (ESE)-detected field-swept spectra were measured at 5 K using the pulse sequence: tp–τ –2t_p– τ –echo. The spectrum was recorded across a field range of 0.05 to 5.55 T with 25000 points, 20 ns long π/2 microwave pulse, τ = 360 ns, shot repetition time = 300 μs, number of shots = 1000, video gain = 33 dB and transmitter level of 27.3%.
All the simulations were obtained using Easyspin[2] 5.2.30 with the function "pepper".[2]

^{57}Fe Nuclear Resonance Vibrational Spectroscopy (NRVS) ^{57}Fe-labelled samples were prepared for nuclear resonance vibrational spectroscopy (NRVS) studies by incubating the ^{57}Fe labelled precursor complex **1** (20 mM) in toluene with the aminating ($PivONH_3OTf$) agent (60 mM) for 15 min and 90 min at room temperature. After incubation the samples were transferred to NRVS cells and frozen in liquid nitrogen. A sample of the ^{57}Fe enriched Fe precursor was also prepared in toluene. Measurements were performed at beam-line P01, PETRA-III, DESY, Hamburg using 14.4 keV radiation produced from a 10 m undulator (32 mm period, 2x157 periods). This was then monochromatized to a resolution of <1 meV using a high heat load monochromator followed by a high resolution monochromator. The spot size on the sample was 3 mm x 1 mm with an incidence angle close to zero. The average photon flux on the sample was ≈ 5 x 10^9 photons per second and inelastic scattering counts were ≈ 1400 per second at the energy of the elastic peak. Spectra are the average of between 21 and 26 scans, measured from – 25 meV (-200 cm^{-1}) to +85 meV (+700 cm^{-1}) with respect to the central position of the elastic peak. Spectra

were processed using routines written in Python by the beam-line scientists at P01, and are presented as the ^{57}Fe density of states.

High Energy Resolution Fluorescence Detected - X-ray absorption spectroscopy (HERFD-XAS):

All X-ray data presented were collected remotely at PIPOX beamline, at the Cornell High Energy Synchrotron Source (CHESS), NY, USA. CHESS synchrotron has a storage ring operating at 6 GeV with a current of 100 mA. A double crystal montochromator Si (311) was utilized upstream for for selecting the energy of the incident beam. Photon flux at the sample with a beam spot size of 200 *µ*m (v) x 400 *µ*m (h) was ~ 2.33 x 10^{11} photons/second, measured at 7150 eV. The monochromatic incident beam was calibrated to the first inflection point of an iron foil (7111.2 eV). The experimental setup for K*β* fluorescence measurements utilized the Dual Array Valence Emission Spectometer (DAVES) with five spherically bent Ge (620) crystals aligned in a Rowland geometry, and a Pilates detector aligned also in the Rowland circle. The energy of DAVES was calibrated using the emission lines of Fe_2O_3 with the maximum of the $K\beta_{1,3}$ set to 7060.6 eV. The spectrometer has an averaged measured resolution, of ~ 1.2 eV, including the resolution of the monochromator. The flight path of the fluorescence emission was filled with a helium-filled bag, located between detector, analyzer crystals and sample. All samples were kept at ~12 K during measurement using a displex cryostat. All spectra were normalized to the incident flux by utilizing a photodiode detector.
Kβ HERFD-XAS spectra were collected with an energy range of 7100 - 7400 eV using energy steps of 0.2 eV between 7100 - 7115, and 0.4 eV step size from 7115 - 7400. Radiation damage studies were performed for each compound by collecting consecutive XAS scans and samples were shown to be stable upto 500-600 seconds. Final HERFD-XAS averages were obtained using PYMCA. Post- and pre- edge background were subtracted and data were normalized such that the edge jump equals to 1. Pre-edge analysis included fitting the spectral shape of each compound and were done utilizing Pseudo-Voigt functions and the least-squared minimization based on the Levenberg-Marquardt algorithm.

Synthesis of $[Fe(acac)_2(H_2O)_2]$[3]: Iron(II) chloride (anhydrous) (0.50 millimole, 63.4 mg, 1 equiv.) was dissolved in degassed water (10 mL) in a 50 mL round bottomed flask containing a small amount of sodium dithionite to reduce any iron(III) impurities under the inert atmosphere of the glove box. To this was added an aqueous solution of piperidine (1.00 millimole, 99 µL, 2 equiv.) and acetylacetone (1.00 millimole, 102 µL, 2 equiv.), dropwise with stirring under the inert atmosphere of the glove box. The yellow solution thus formed was stirred for 15-20 min, to isolate a yellow precipitate. The precipitate was filtered under vacuum, washed with water, followed by 1 mL of ethanol and 2 mL of diethyl ether. The solid thus isolated was vacuum dried and isolated as a dark yellow powder. Yield: 0.10 g (71%). Elemental analysis calcd (%) for $C_{10}H_{18}FeO_6$ (290.09 $gmol^{-1}$): C 41.40, H 6.25; found: C 41.13, H 6.26. UV-Vis in dichloromethane: λ_{max} = 353 nm, ε_{353} = 1776 $M^{-1}cm^{-1}$ and λ_{max} = 437 nm, ε_{437} = 1751 $M^{-1}cm^{-1}$.

Catalytic reaction: A screw cap vial was charged with $[Fe(acac)_2(H_2O)_2]$ (**1**) (10 mol%, 0.05 mmol) and a stirring bar in the glove box. To it 1 mL of solvent [CH_2Cl_2:CH_3OH (1:3)] was added via a syringe followed by immediate addition of solid aminating agent $PivONH_3OTf$ (1.25 mmol) followed by styrene (0.5 mmol) with a syringe. To the resulting mixture, an additional 0.5 mL solvent mixture CH_2Cl_2:CH_3OH (1:3) was added and the reaction mixture was sealed and stirred in the glove box at room temperature for 20 h. This is referred to as the standard reaction condition. After 20 h the reaction mixture was diluted with Methyl tert-butyl ether (MTBE, 15 mL) and extracted with an aqueous solution of HCl 1.0 M (3 x 15 mL). The combined water phases were concentrated in vacuo to afford the crude product as the free amine salt. The crude product was directly used for Boc-protection prior to isolation following the previous literature report.[4] The *N*-Boc protected product was loaded on silica gel and purified via flash column chromatography (DCM-MeOH-TEA 100:15:1.5 as eluent) to afford *tert*-butyl (2-hydroxy-2-phenylethyl)carbamate (*N*-Boc protected 2-methoxy-2-phenylethan-1-amine) with 60% isolated yield. ^{1}H-NMR (500 MHz, $CDCl_3$): δ = 7.37 – 7.27 (m, 5H), 4.96 (br s, 1H), 4.82 (dd, J = 8.0, 3.2 Hz, 1H), 3.49 – 3.46 (br m, 1H), 3.24 (dd, J = 13.6, 8.0 Hz, 1H), 2.89 (br s, 1H), 1.44 (s, 9H).[4]

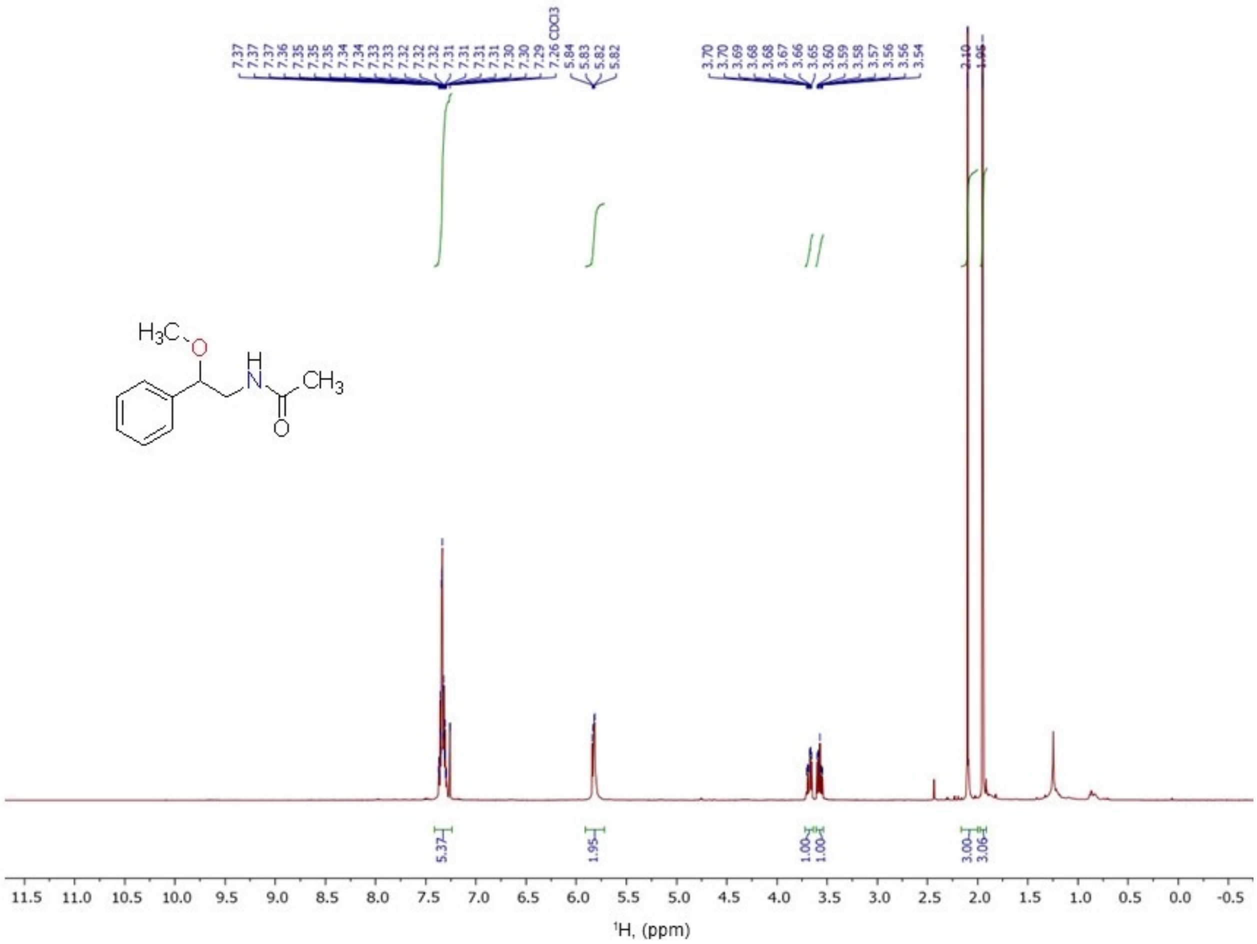

Figure S1. ^{1}H NMR of isolated *tert*-butyl (2-hydroxy-2-phenylethyl)carbamate in $CDCl_3$, 500 MHz after work up and purification of the reaction of **1** and $PivONH_3OTf$ with styrene.

Control Experiments: The same catalytic reaction procedure described above was followed in the absence of the iron catalyst **1**. No product formation was observed in the reaction of the aminating agent $PivONH_3OTf$ with styrene, in the absence of the iron catalyst. Styrene was recovered almost quantitatively.

UV-vis Deconvolution: The UV-vis absorption spectra were deconvoluted with Gaussian bands for analysis. For all the three species (**1**, **Int I** and **Int II**) we see four primary features (A, B, C, D) (Figure S2) that vary in their energies and intensities, as summarized in Table S1. The largest difference across the series is a clear red shift of the lowest-energy excitation (A). The high extinction coefficients and oscillator strength of the observed bands point to their possible assignment as metal-to-ligand charge transfer (MLCT) and

ligand-to-metal charge transfer transitions. The very intense high-energy excitations (C and D) may be attributed to ligand-ligand transitions, consistent with the fact that these peaks are only slightly shifted when going from precursor **1**→ **Int I** → **Int II**. More detailed assignments will be presented in a later section (computational section) on the basis of our quantum chemical calculations.

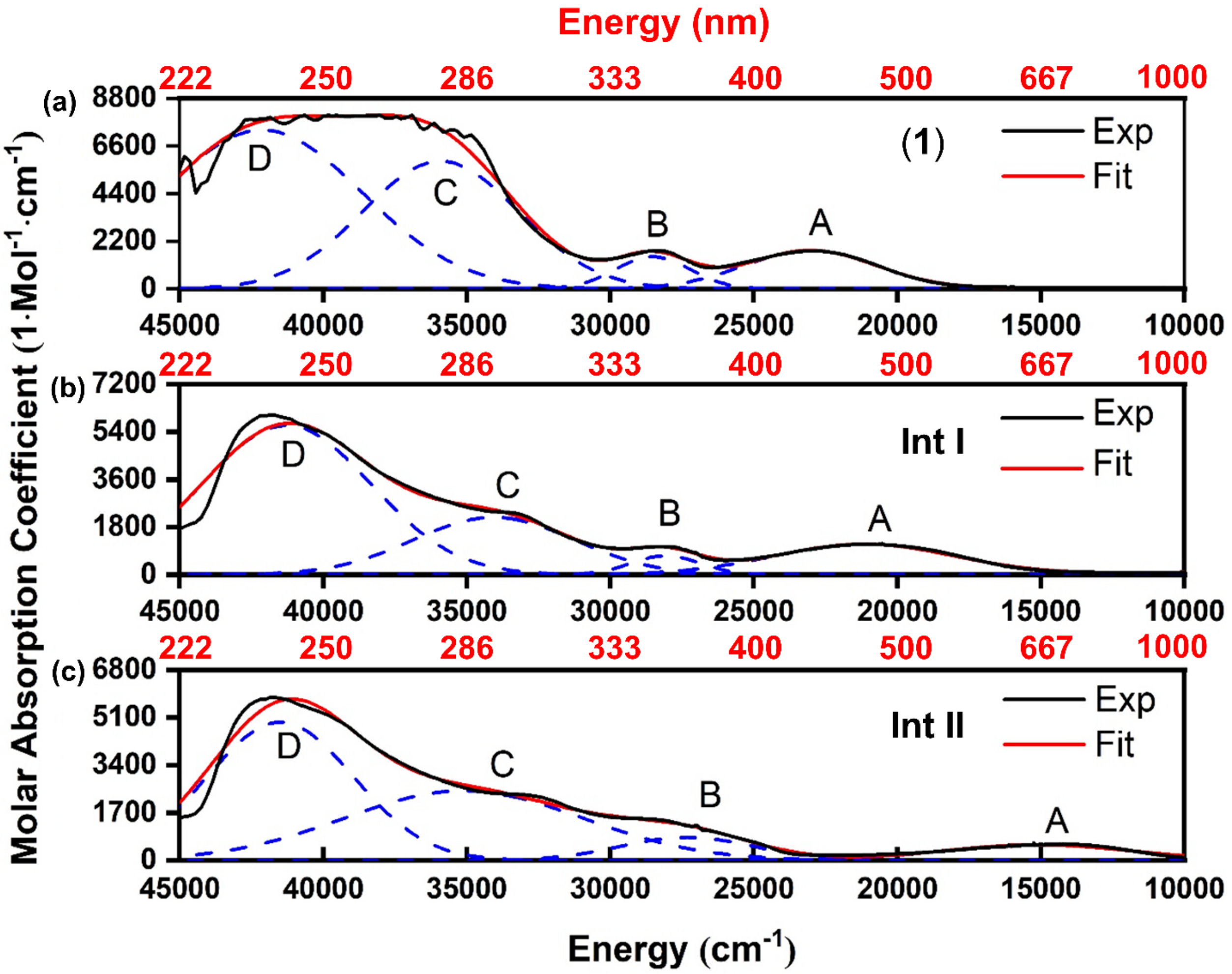

Figure S2. Experimental optical absorption spectra with gaussian fit. (a) Precursor [$Fe^{II}(acac)_2(H_2O)_2$] (**1**) (top). (b) **Int I** (middle). (c) **Int II** (bottom). In red are the X-axis scale in nm (top) and in black is the X-axis scale in cm^{-1} (bottom).

Table S1. Excitation energies and oscillator strengths obtained from gaussian deconvolution of the experimental absorption spectra.

	Excitation energy (cm^{-1})	Oscillator strength	Excitation energy (cm^{-1})	Oscillator strength	Excitation energy (cm^{-1})	Oscillator strength
	Precursor **1**		**Int I**		**Int II**	
A	22806	0.044	20656	0.040	14342	0.019
B	28459	0.021	28018	0.009	27128	0.021
C	35702	0.173	33819	0.066	34606	0.112
D	41837	0.274	41070	0.180	41339	0.136

Kinetic studies: Kinetic measurements were performed on an Agilent 8453 diode-array spectrophotometer. Reactions were run in a 1-cm UV cuvette and followed by monitoring UV-vis spectral changes of reaction solutions under Ar atmosphere. The kinetic experiments with various styrenyl olefins were performed under pseudo-first-order conditions by adding appropriate amounts of substrates (0.005-0.20 M) to the solution of **1** (0.25 mM) and excess PiVONH$_3$OTf (10 equiv.) in CH_2Cl_2:CH_3OH (3:1) solvent mixture, thereby generating **Int II**, under anaerobic conditions. The decay of the charge transfer band at 700 nm corresponding to **Int II** was monitored spectrophotometrically. Rate constants, (k_{obs}) were determined by pseudo-first-order fitting of the absorbance vs time plot. Second order rate constants (k'_2) were obtained from the slope of the linear fit of k_{obs} vs substrate concentration considering the correction for self-decay. All experiments were repeated in triplicate and the data reported represent the average of these reactions. Addition of styrene to **1** in the presence of excess PivONH$_3$OTf did not lead to accumulation of **Int II**, so all kinetic measurements with styrene were performed after generating **Int II**, and then monitoring the decay of the chromophore at 700 nm corresponding to **Int II**.

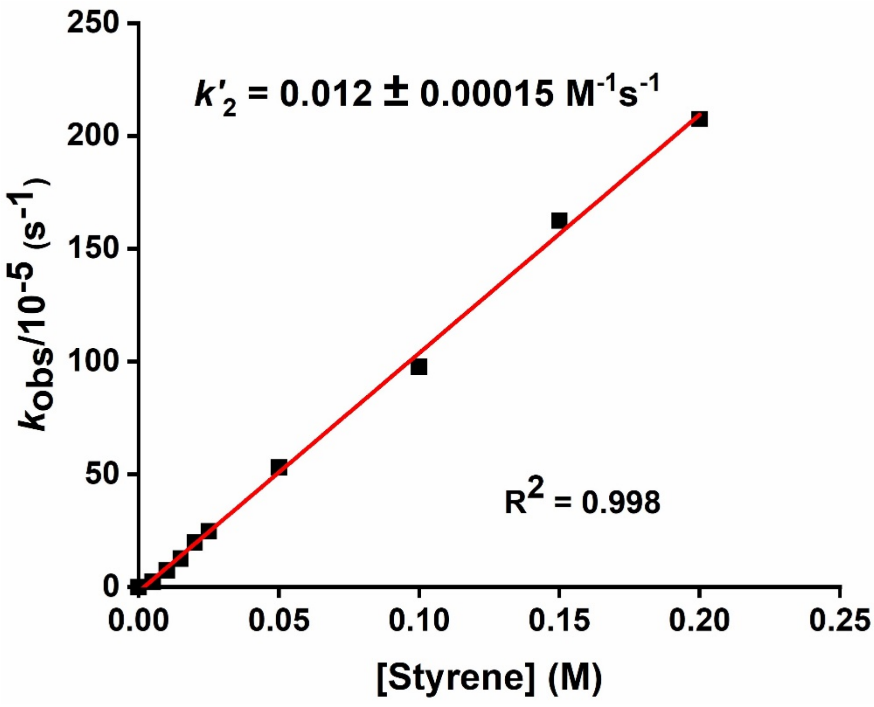

Figure S3. Plots of k_{obs} vs substrate concentration for the reaction of **Int II** (**1** and $PivONH_3OTf$ after 90 min) with styrene.

Table S2. Table showing the reaction rate of decay of the UV-vis chromophore at 700 nm at various styrene concentrations

Entry	Conc. of styrene (M)	Corrected Rate X 10^{-5} ($M^{-1}s^{-1}$) (k'_{obs})
1.	0	0
2.	0.005	2.3
3.	0.010	7.6
4.	0.015	12.7
5.	0.20	19.8
6.	0.025	24.8
7.	0.050	53.1
8.	0.100	97.7
9.	0.150	162.5
10.	0.200	207.5

Table S3. Table showing the reaction rate of decay of the UV-vis chromophore at 700 nm at various 4-methyl styrene concentrations

Entry	Conc. of 4-methyl styrene (M)	Corrected Rate X 10^{-5} ($M^{-1}s^{-1}$) (k'_{obs})
1.	0	0
2.	0.005	27.5
3.	0.010	30.5
4.	0.020	69.5
5.	0.025	83.0
6.	0.050	255.5
7.	0.100	333.5
8.	0.150	507.5
9.	0.200	651.5

Table S4. Table showing the reaction rate of decay of the UV-vis chromophore at 700 nm at various 4-OMe styrene concentrations

Entry	Conc. of 4-OMe styrene (M)	Corrected Rate X 10^{-5} ($M^{-1}s^{-1}$) (k'_{obs})
1.	0	0
2.	0.005	17.9
3.	0.010	56.3
4.	0.020	102.7
5.	0.025	117.9
6.	0.050	246.7
7.	0.100	479.5
8.	0.150	697.5
9.	0.200	897.5

Table S5. Table showing the reaction rate of decay of the UV-vis chromophore at 700 nm at various 4-Cl styrene concentration

Entry	Conc. of 4-Cl styrene (M)	Corrected Rate X 10^{-5} ($M^{-1}s^{-1}$) (k'_{obs})
1.	0	0
2.	0.005	1.3
3.	0.010	6.0
4.	0.020	15.7
5.	0.025	19.0
6.	0.050	48.8
7.	0.100	96.1
8.	0.150	147.0
9.	0.200	187.1

Table S6. Table showing the reaction rate of decay of the UV-vis chromophore at 700 nm at various 4-Br styrene concentrations

Entry	Conc. of 4-Br styrene (M)	Corrected Rate X 10^{-5} ($M^{-1}s^{-1}$) (k'_{obs})
1.	0	0
2.	0.005	0.6
3.	0.010	5.3
4.	0.015	7.7
5.	0.020	12.6
6.	0.025	16.7
7.	0.050	43.1
8.	0.100	83.1
9.	0.150	139.8
10.	0.200	169.8

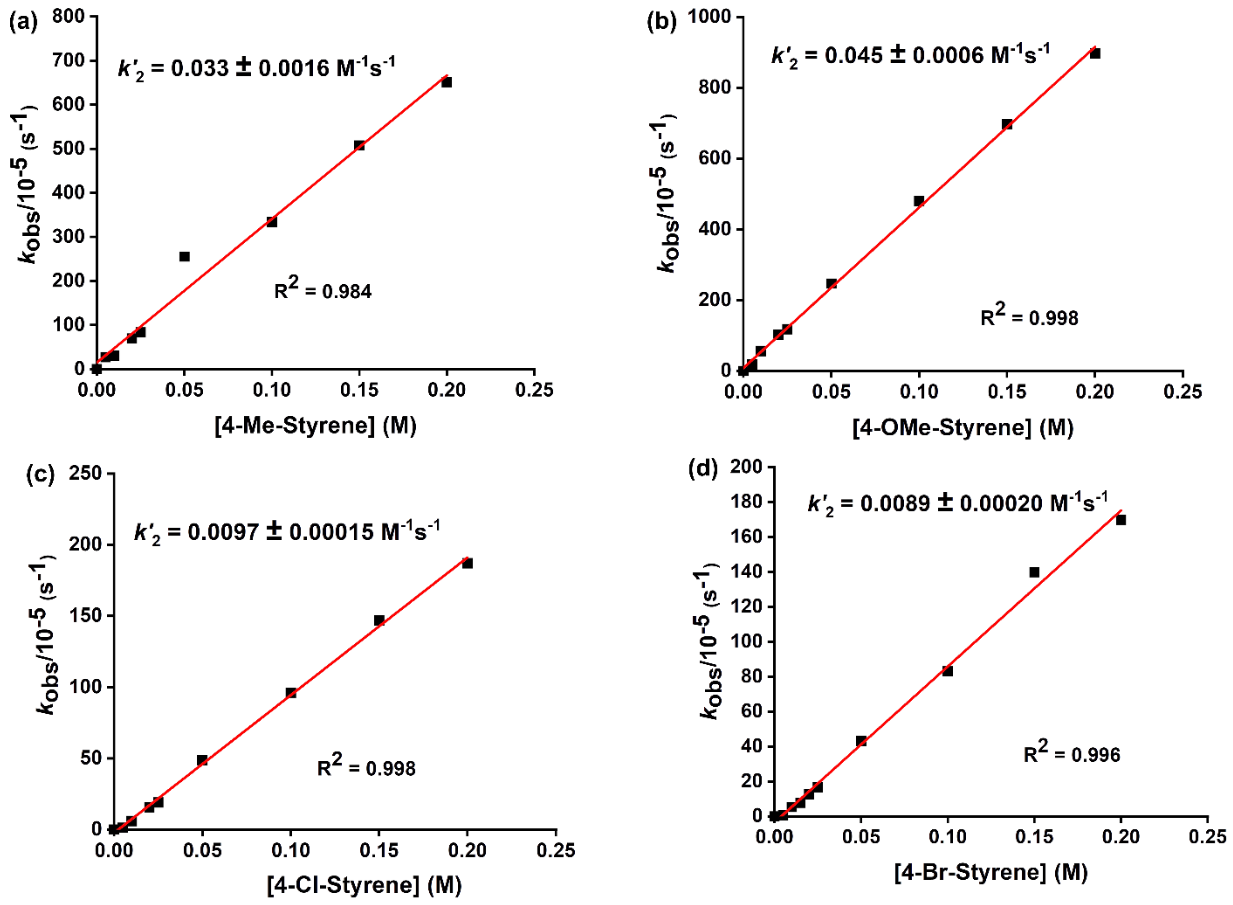

Figure S4. Plots of k_{obs} vs substrate concentration for the reaction of **Int II** (**1** and PivONH$_3$OTf after 90 min) with (a) 4-Me styrene (b) 4-OMe styrene (c) 4-Cl styrene and (d) 4-Br styrene at 293 K.

Hammett Analyses: The second order rate constants (k'_2) for different *p*-X-substituted styrenes were determined from the slope of the linear fit of k_{obs} vs substrate concentration from UV-vis diode array spectrophotometer (see previous section, Figure S4). For Hammett analyses considering only electronic effects, and not including radical stabilization effects, plots of the second order rates $\log(k_X/k_H)$ for various *p*-X-substituted styrenes and styrene, versus the electronic (σ+) parameter, linear correlations were found for the aminomethoxylation reaction of *p*-X-substituted styrene by **Int II**. (R^2 = 0.92, ρ^+ = -0.77, Figure S8, Table S7).

Plotting $\log(k_X/k_H)$ versus $\rho^{\bullet}\sigma^{\bullet} + \rho^+\sigma^+ + C$ where both the radical spin delocalisation ($\sigma^{\bullet}$) and electronic parameter (σ^+) have been considered and applying multiple linear regression, a linear correlation was obtained for the aminomethoxylation reaction of *p*-substituted styrenes by **Int II**. (R^2 = 0.92, $\rho^{\bullet}$ = - 0.08, ρ^+ = -0.78, C = 0.05, $\rho^+/\rho^{\bullet}$ = 9.75, slope ρ = 1, Figure S9, Table S8) which indicated that electronic effects dominate the reaction rate. In this formula, σ• and σ+ are reported in the literature and ρ• and ρ+ are found by the multiple component linear regression.[5-6] Such high $|\rho^+/\rho^{\bullet}|$ ratios, indicate the electronic effects are more important than radical stabilization effects.[6] Both the ρ^+ values as well as R^2 values were found to be similar for the two Hammett plots considering (i) only electronic effects and (ii) a combination of both spin and electronic effects, thus implicating inclusion of radical stabilization does not give a more accurate description of the transition states. This seems to contradict the proposed radical nature of **Int II**, a high spin Fe(III)-iminyl radical species, which interacts with styrene. However, a point to note here, is that we are estimating the Hammett parameters for aminomethoxylation of styrene by **Int II**, which involves a two-step reaction where the first step is the *N*-transfer reactivity of the radical **Int II** to styrene to form an aziridine product, followed by the second step, of the ring opening reaction of aziridine, in the presence of a protic acid (HOTf) and nucleophilic solvent (CH_3OH), involving highly charged transition states to form the final amino ether product (Figure 9, main manuscript). Hammett analyses thus overall predict an electrophilic pathway, with predominantly electronic effect for the aminomethoxylation of styrene by **Int II**.

Table S7. Hammett σ^+ parameters for *p*-X substituted styrenes and obtained ratios for k_X and k_H in the aminomethoxylation by **Int II** (formed by the reaction of **1** and $PivONH_3OTf$ in CH_2Cl_2:CH_3OH solvent mixture at 293 K by 90 min).

Entry	*p*-X substitutent on styrene	σ^+	Second order rate constant (*k*) $M^{-1}s^{-1}$	k_X/k_H	log (k_x/k_H)
1.	-OMe	-0.77	0.045	3.75	0.57
2.	-Me	-0.311	0.033	2.75	0.44
3.	-H	0	0.012	1	0
4.	-Cl	0.114	0.0097	0.81	-0.092
5.	-Br	0.23	0.0089	0.74	-0.13

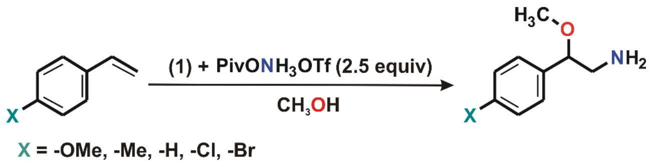

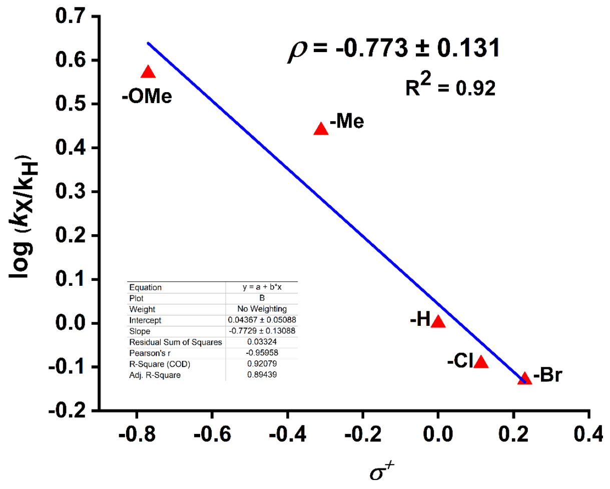

Figure S5. Hammett plot of the logarithm of the ratio of rate constants (log k_X/k_H) vs. σ^+ values of *p*-X-styrenes (X = OMe, Me, H, Cl and Br) for the aminomethoxylation reaction of *p*-X-styrene derivatives by **1** and $PivONH_3OTf$ in CH_2Cl_2:CH_3OH solvent mixture at 293 K.

Table S8. Hammett σ^+ and $\sigma^\bullet$ parameters for *p*-X substituted styrenes and obtained ratios for k_X and k_H in the aminomethoxylation reaction by **Int II** (formed by the reaction of **1** and PivONH$_3$OTf in CH_2Cl_2:CH_3OH solvent mixture at 293 K by 90 min).

Entry	p-X Substitutent	σ+	ρ^+	$\sigma\bullet$	$\rho\bullet$	X axis y= C + $\rho^+\sigma^+$ + $\rho\bullet\sigma\bullet$	Log (k_x/k_H)
1.	-OMe	-0.77	-0.78	0.23	-0.08	0.6369	0.57
2.	-Me	-0.311	-0.78	0.15	-0.08	0.28528	0.44
3.	-H	0	-0.78	0	-0.08	0.0547	0
4.	-Cl	0.114	-0.78	0.22	-0.08	-0.05182	-0.092
5.	-Br	0.23	-0.78	0.13	-0.08	-0.1355	-0.13

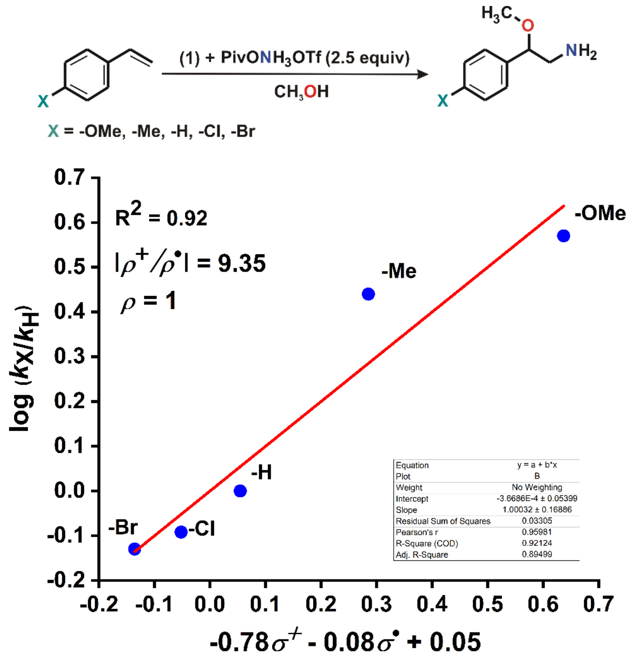

Figure S6. Hammett plot of the logarithm of the ratio of rate constants (log k_X/k_H) vs. C + $\rho^+\sigma^+$ + $\rho\bullet\sigma\bullet$ values of *p*-X-styrenes (X = OMe, Me, H, Cl and Br) for the aminomethoxylation reaction of *p*-X-styrene derivatives by **1** and PivONH$_3$OTf in CH_2Cl_2:CH_3OH solvent mixture at 293 K.

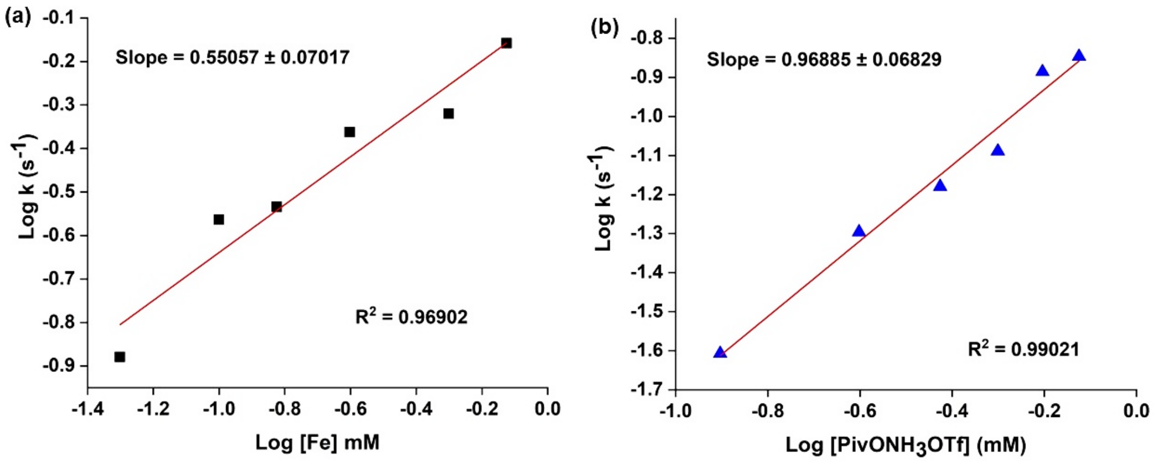

Figure S7. Log-Log plot of (a) rate constant vs concentration of [Fe(acac)$_2$(H$_2$O)$_2$] (**1**) and (b) rate constant vs concentration of aminating agent (PivONH$_3$OTf) in the reaction of **1** and PivONH$_3$OTf to generate **Int I** as obtained from stopped flow UV-vis measurement. The slope of 0.55 is consistent with a half order reaction w.r.t [Fe] and a slope of ~1 corresponds to a first order dependence on [PivONH$_3$OTf]. The overall fractional order coefficient of the reaction is suggestive that there are multiple steps involved in the reaction (see catalyst activation section for details).

ESI-Mass Analyses: Analyses of the reaction solution of **Int I** by electrospray ionization mass spectrometry (ESI-MS) in CH$_2$Cl$_2$:CH$_3$OH (3:1) displays an ion peak at *m/z* = 371.1 with the isotope distribution pattern attributable to [Fe(acac)$_2$(PivONH) + H$^+$] [C$_{15}$H$_{25}$FeNO$_6$ +H$^+$] (Scheme 3 and Figure S8, SI). Performing the same reaction in a deuterated solvent mixture of CD$_2$Cl$_2$:CD$_3$OD (3:1), and analyses by ESI-MS, reveals that along with the peak at 371.1, another peak at *m/z* = 372.1 with an isotope distribution pattern corresponding to [Fe(acac)$_2$(PivOND) + H$^+$] [C$_{15}$H$_{24}$D$_1$Fe$_1$N$_1$O$_6$ +H$^+$] appears, implicating an exchangeable proton in the NH species coordinated to Fe(acac)$_2$ (Scheme 3, Figure S9). For **Int II**, the ESI-mass spectrum of the reaction solution shows ion peaks at *m/z* = 269.03 and *m/z* = 270.04 with the isotope distribution patterns attributable to [Fe(acac)$_2$(NH)]$^+$ and [Fe(acac)$_2$(NH) + H$^+$] (Scheme 3 and Figure S10), implicating the loss of O-pivaloyl group (-OPiv) from **Int I**. Similar to **Int I**, ESI-mass analyses of **Int II** in deuterated solvent mixture of CD$_2$Cl$_2$:CD$_3$OD (3:1), confirms an exchangeable proton in the –NH group coordinated to the Fe(acac)$_2$ unit (Scheme 3, Figure S11). Performing the

same reactions with labelled ^{15}N reagent ($PivO^{15}NH_3OTf$) shifts the peak for **Int I** by one unit to *m/z* = 372.09 (Scheme 3, Figure S12) and **Int II** to *m/z* = 270.03 and 271.03 (Scheme 3, Figure S13), matching the isotope distribution pattern for ^{15}N incorporation and further confirming the source of nitrogen from the hydroxyl amine derived reagent ($PivO^{15}NH_3OTf$) in the putative iron-nitrogen intermediates (**Int I** and **Int II**).

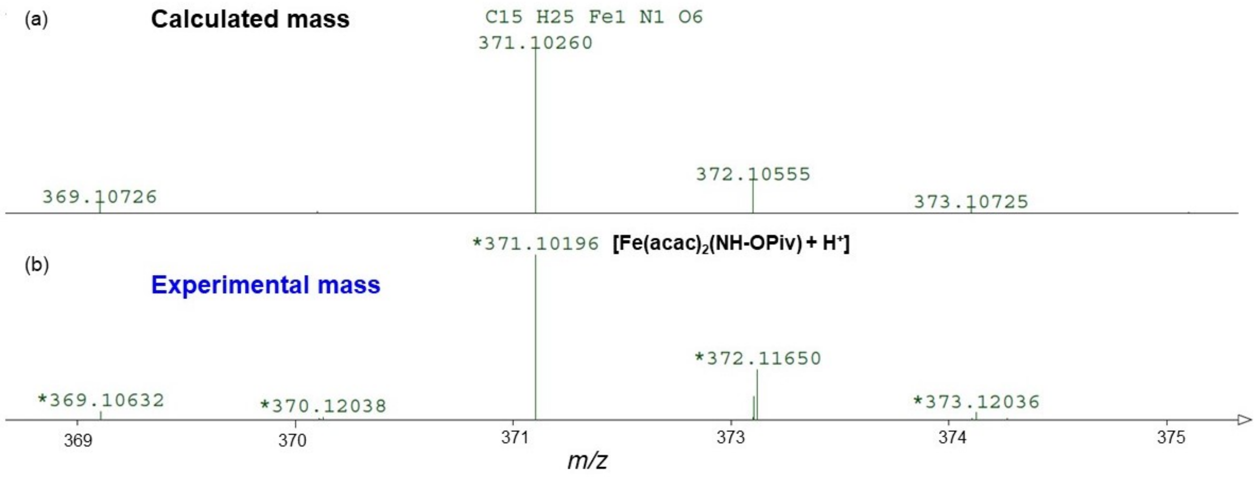

Figure S8. ESI-mass spectrum (positive ion mode in CH_3OH) of $[Fe(acac)_2(H_2O)_2]$ (**1**) + $PivONH_3OTf$ (3 equiv.) in CH_2Cl_2:CH_3OH (3:1) within 15 min of mixing. (a) The calculated isotope distribution pattern of the ion peak at *m/z* 371.1(b) The experimental spectra showing the isotope distribution pattern of 371.1 peak and matching with the sum formula corresponding to **Int I** ($[Fe(acac)_2(NH\text{-}OPiv) + H^+]$.

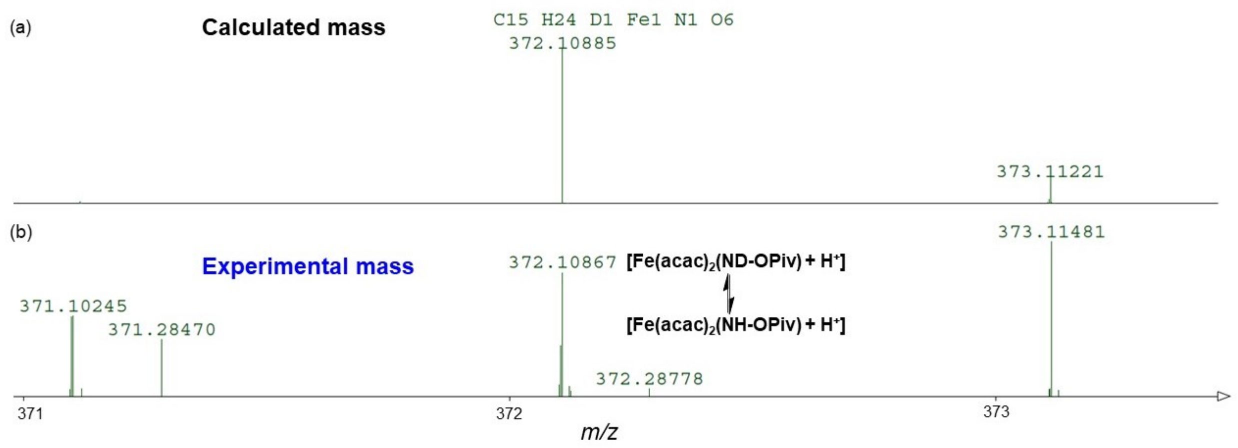

Figure S9. ESI-mass spectrum (positive ion mode in CH_3OH) of $[Fe(acac)_2(H_2O)_2]$ (**1**) + $PivONH_3OTf$ (3 equiv.) in CD_2Cl_2:CD_3OH (3:1) within 15 min of mixing. (a) The calculated isotope distribution pattern of the ion peak at *m/z* 372.1(b) The experimental spectra showing the isotope distribution pattern of *m/z* 372.1 peak and matching with the sum formula corresponding to D-exchanged **Int I** ($[Fe(acac)_2$(ND-OPiv) +$H^+]$.

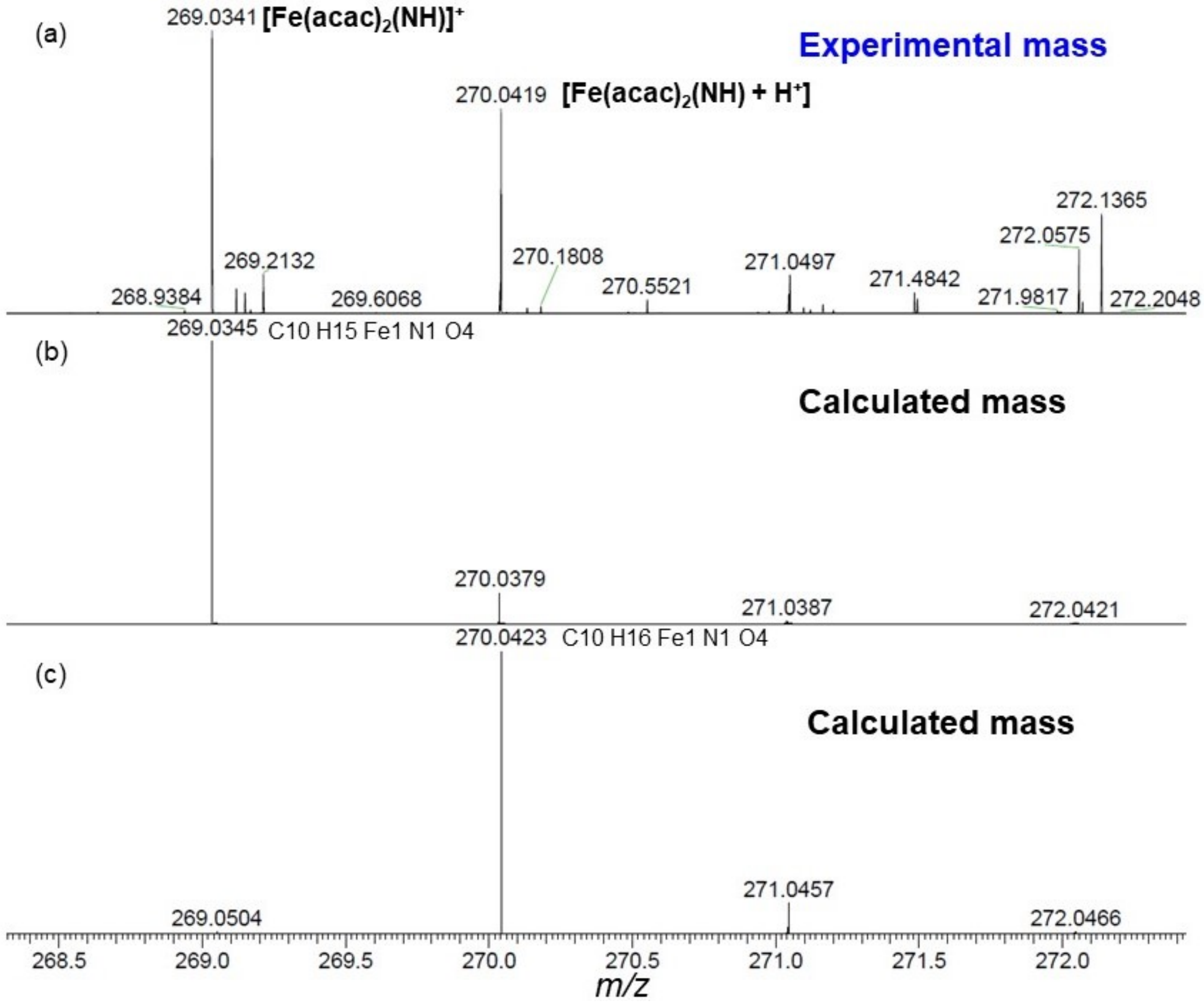

Figure S10. ESI-mass spectrum (positive ion mode in CH_3OH) of $[Fe(acac)_2(H_2O)_2]$ (**1**) + $PivONH_3OTf$ (3 equiv.) in CH_2Cl_2:CH_3OH (3:1) within 90 min of mixing. (a) The experimental spectra showing the isotope distribution pattern of *m/z* 269.03 and 270.04 peaks and matching with the sum formula corresponding to **Int II** ($[Fe(acac)_2(NH)]^+$ and $[Fe(acac)_2(NH)]$ $+H^+$).(b) The calculated isotope distribution pattern of the ion peak at *m/z* 269.03. (c) The calculated isotope distribution pattern of the ion peak at *m/z* 270.04.

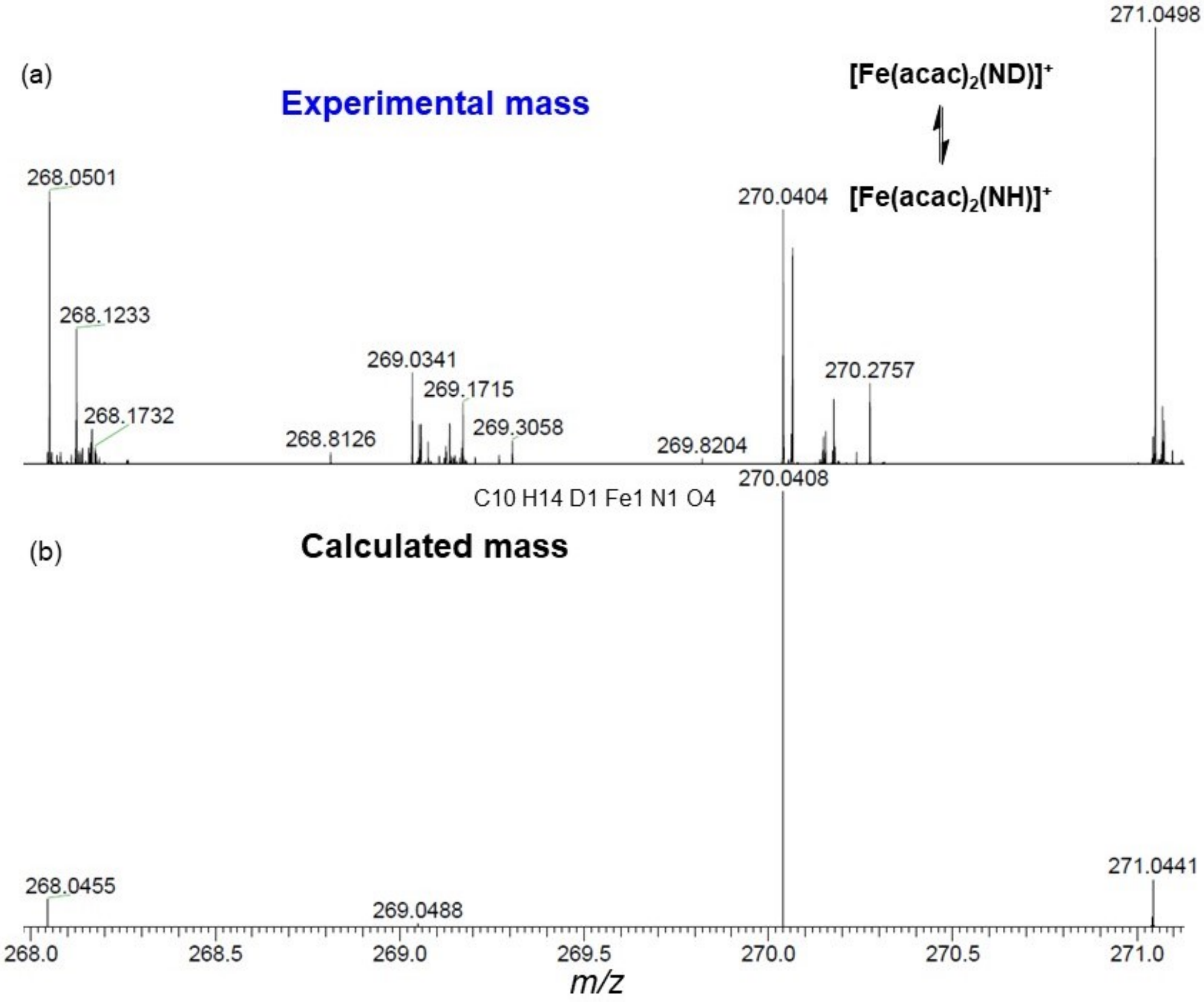

Figure S11. ESI-mass spectrum (positive ion mode in CH_3OH) of $[Fe(acac)_2(H_2O)_2]$ (**1**) + $PivONH_3OTf$ (3 equiv.) in CH_2Cl_2:CH_3OH (3:1) within 90 min of mixing. (a) The experimental spectra showing the isotope distribution pattern of *m/z* 270.04 peak and matching with the sum formula corresponding to D-exchanged **Int II** ($[Fe(acac)_2(ND)]^+$. (b) The calculated isotope distribution pattern of the ion peak at *m/z* 270.04.

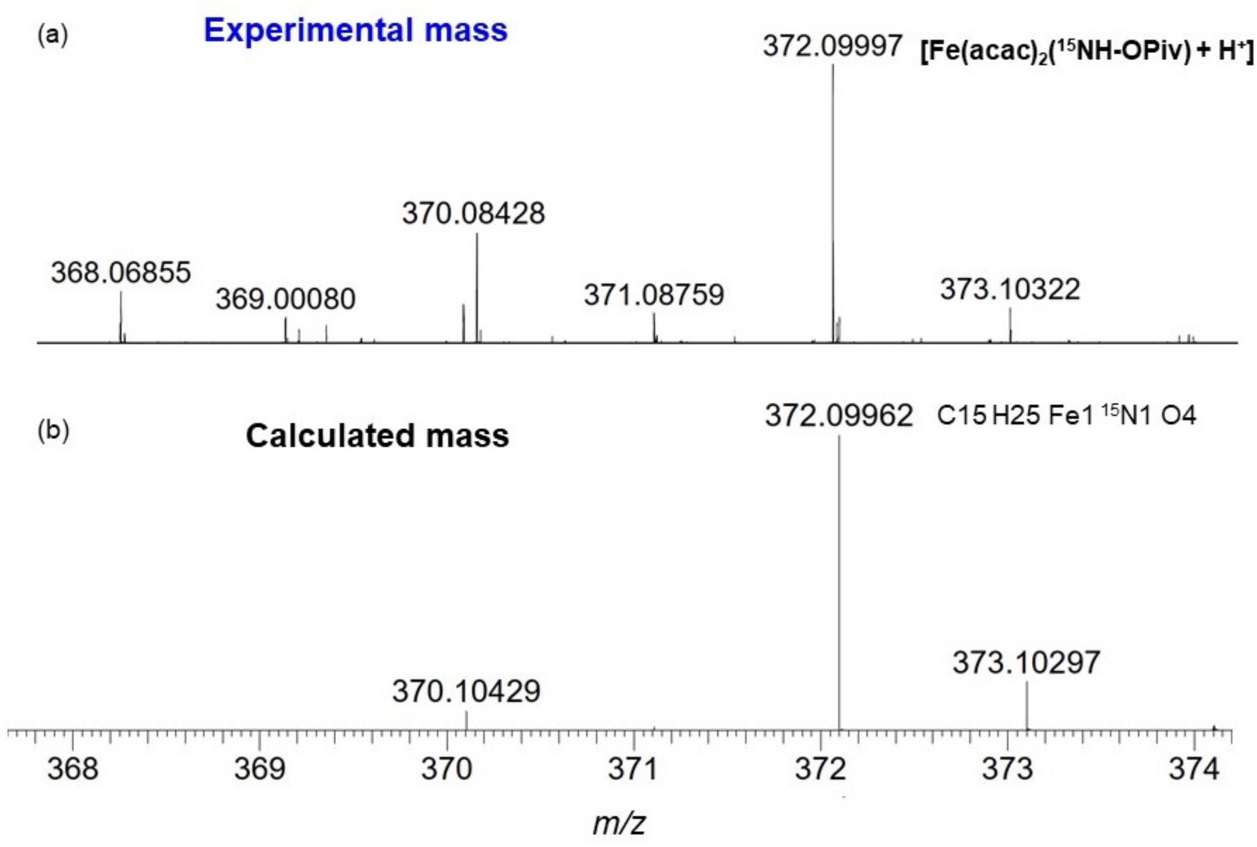

Figure S12. ESI-mass spectrum (positive ion mode in CH_3OH) of [Fe(acac)$_2$(H_2O)$_2$] (**1**) + PivO^{15}NH$_3$OTf (3 equiv.) in CH_2Cl_2:CH_3OH (3:1) within 15 min of mixing. (a) The experimental spectra showing the isotope distribution pattern of 372.09 peak and matching with the sum formula corresponding to **Int I** with ^{15}N labelled nitrogen i.e ([Fe(acac)$_2$(^{15}NH-OPiv) +H$^+$]. (b) The calculated isotope distribution pattern of the ion peak at *m/z* 372.09

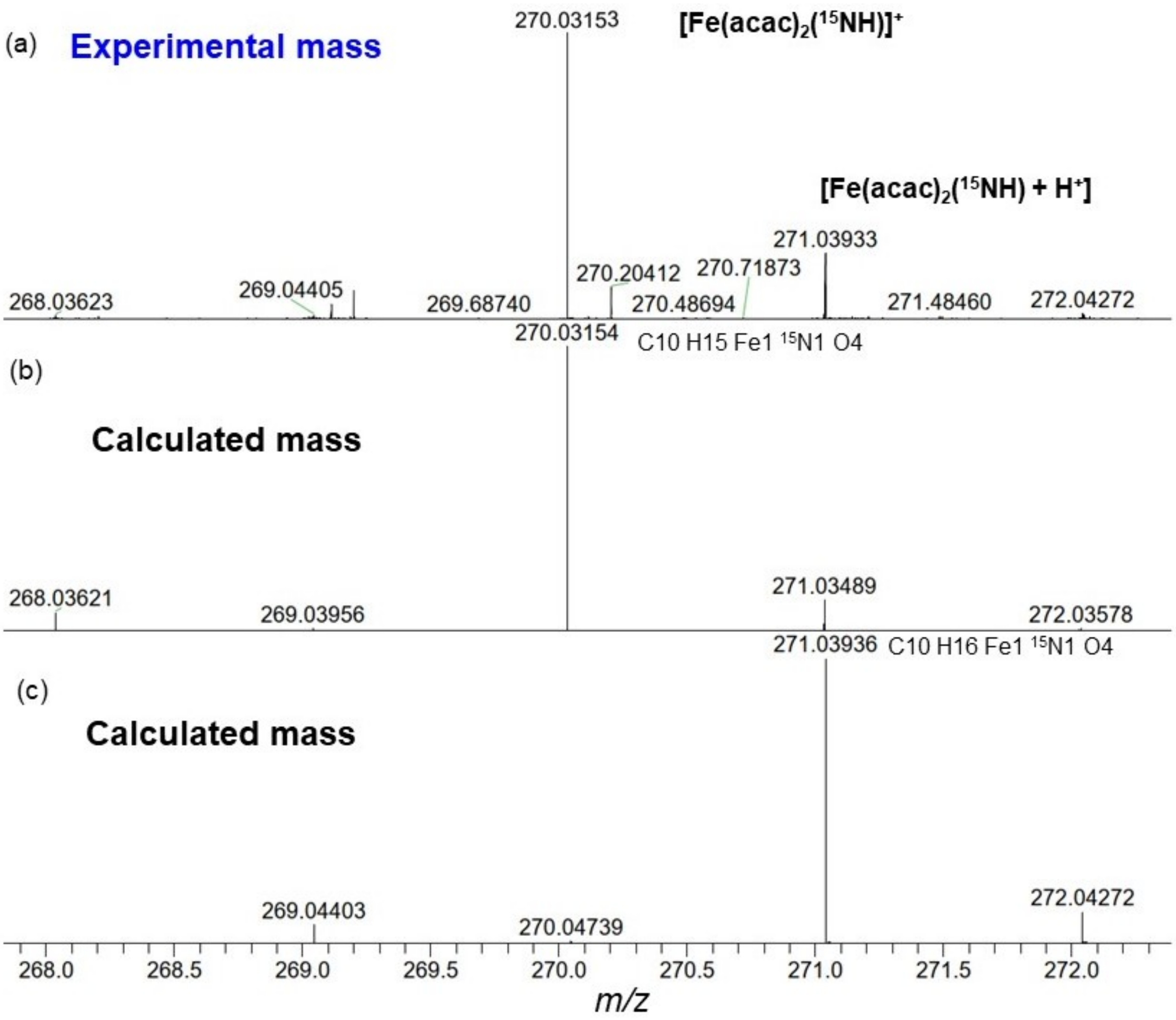

Figure S13. ESI-mass spectrum (positive ion mode in CH_3OH) of $[Fe(acac)_2(H_2O)_2]$ (**1**) + $PivO^{15}NH_3OTf$ (3 equiv.) in CH_2Cl_2:CH_3OH (3:1) within 90 min of mixing. (a) The experimental spectra showing the isotope distribution pattern of *m/z* 270.04 and 271.03 peaks and matching with the sum formula corresponding to **Int II** ($[Fe(acac)_2(^{15}NH)]^+$ and $[Fe(acac)_2(^{15}NH)] + H^+$).

(b) The calculated Isotope distribution pattern of the ion peaks at *m/z* 269.03 and (c) The calculated isotope distribution pattern of the ion peaks at *m/z* 270.04.

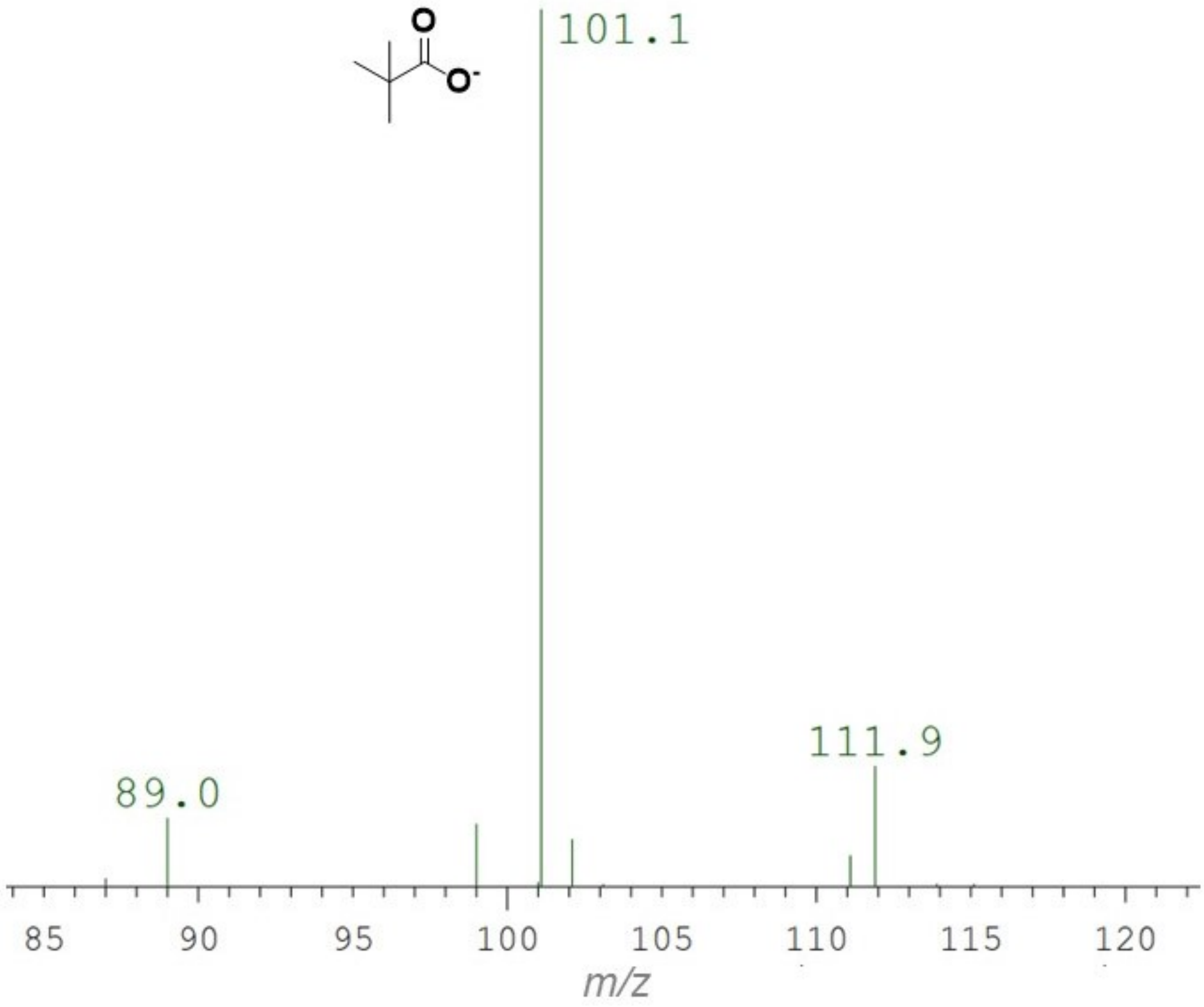

Figure S14. ESI-mass spectrum (negative ion mode in CH_3OH) of the organic product derived from $PivONH_3OTf$ after mixing $[Fe(acac)_2(H_2O)_2]$ (**1**) + $PivONH_3OTf$ (3 equiv.) in CH_2Cl_2:CH_3OH (3:1).

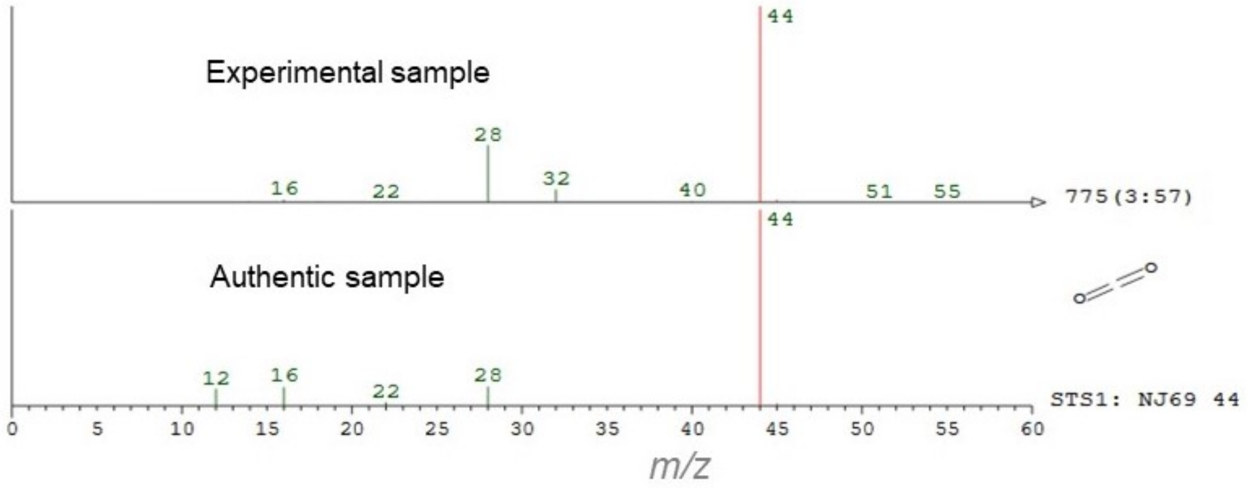

Figure S15. GC-mass spectrum of the organic product derived from $PivONH_3OTf$ after mixing $[Fe(acac)_2(H_2O)_2]$ (**1**) + $PivONH_3OTf$ (3 equiv.) in CH_2Cl_2:CH_3OH (3:1) and measuring within 90 min.

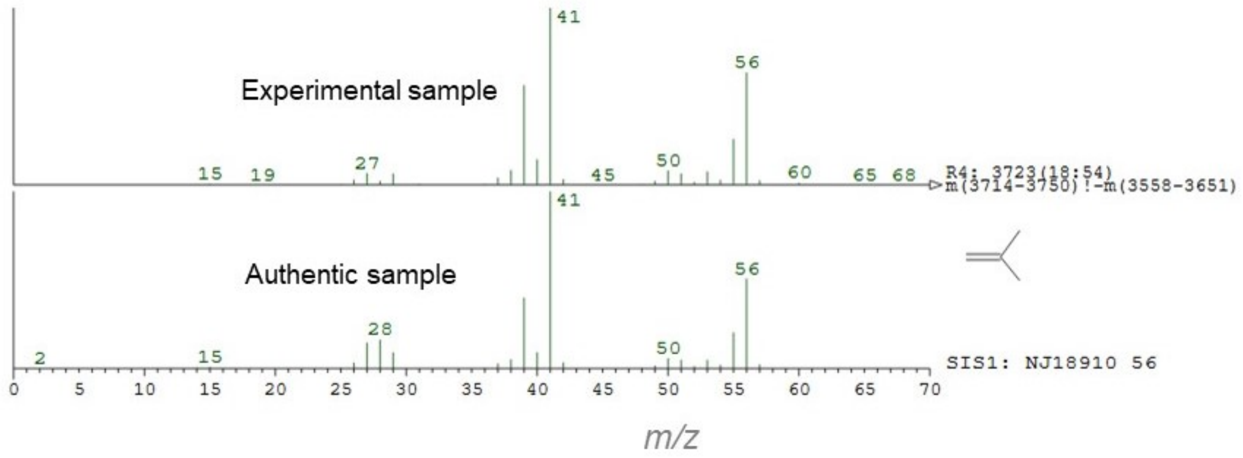

Figure S16. GC-mass spectrum of the organic product derived from $PivONH_3OTf$ after mixing $[Fe(acac)_2(H_2O)_2]$ (**1**) + $PivONH_3OTf$ (3 equiv.) in CH_2Cl_2:CH_3OH (3:1) and measuring within 90 min.

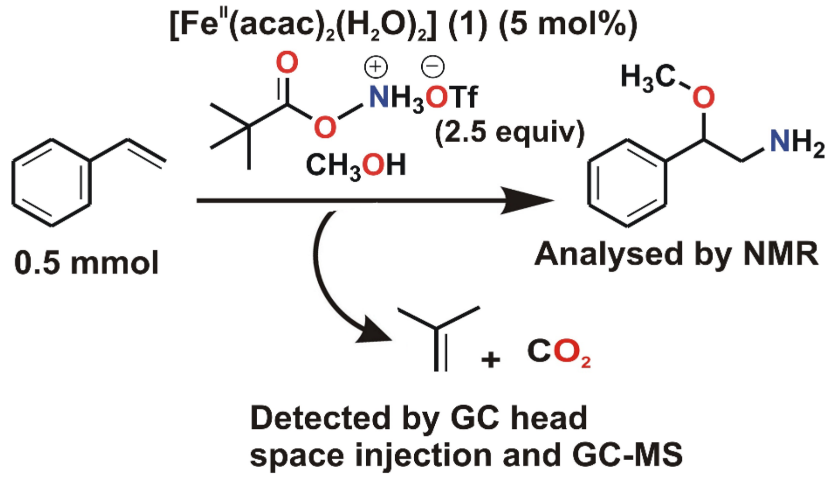

Scheme S1. Organic product analysis to probe the heterolytic vs homolytic N-O bond cleavage pathways for conversion of **Int I** to **Int II** in the iron catalyzed aminomethoxylation of styrene.

EPR

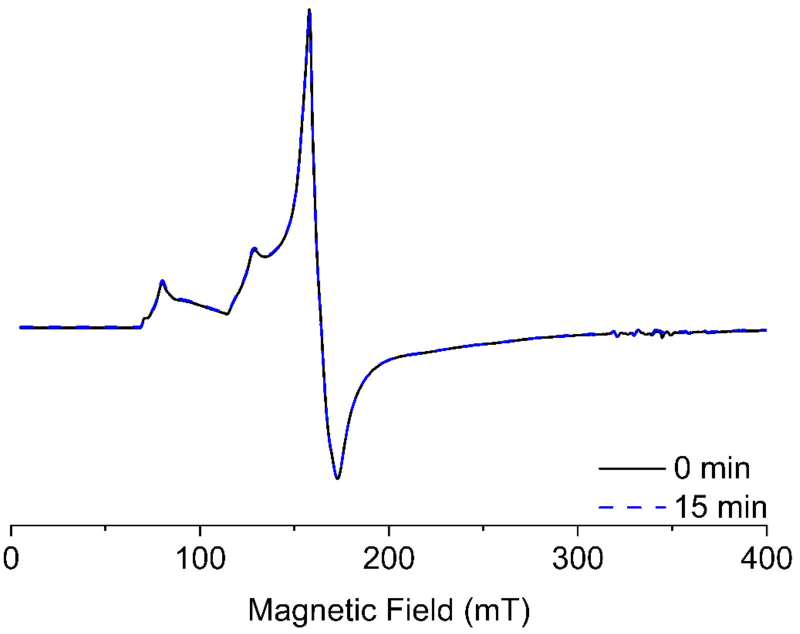

Figure S17. X-band EPR spectra of **Int I** recorded immediately (0 min, black) after mixing **1** and PivONH$_3$OTf and after 15 min (dashed blue) at 10 K.

Spin Quantification

Upon quantification using Cu(II) as a reference standard, **Int I** was found to be ~65-75% with respect to the iron content of the starting precursor based on the double integrals.

Spectral simulations

All spectral simulations have been obtained using Easyspin[2] 5.2.30 with the function "pepper"[2] and the following Spin Hamiltonian (SH) model:

$$\widehat{H} = \mu_{\mathrm{B}}\boldsymbol{S}\boldsymbol{g}\boldsymbol{B_0} + {}^{D}\!/_{3}\,(3S_z^2 - S(S+1)) + E(S_x^2 - S_y^2) \qquad (1)$$

Here, the first term denotes the electron Zeeman interaction, with the spin angular momentum, $\boldsymbol{S}$, the external magnetic field, $\boldsymbol{B_0}$, and the anisotropic $\boldsymbol{g}$-tensor. The second and third term are the ZFS terms, with the spin quantum number, S, the spin matrices, $S_{x,y,z}$, and the axial and transverse ZFS parameters, *D* and *E*, respectively.

Plotted in Figure S18 is the experimental X-band EPR spectra of **Int I** (a) alongside simulated spectra (b) obtained with the given ZFS- and g-values. In addition, deconvolution of the simulated spectrum is shown in c) and d).

EPR spectra of high-spin (S > 1/2) iron states are most sensitive to ZFS, which is an anisotropic property parameterized by an axial (*D*) and a rhombicity parameter *E*/*D* (eq.1 in the SI). The latter can take values between 0 and 1/3*.* The effective g values of the **Int I** spectrum in the range g_{eff} = 9 – 4.3 (Figure 4b) are typical of S = 5/2 systems with large ZFS (|*D*| > ≈ 0.3 cm^{-1}, *i.e.* the energy of microwave quanta at 9.6 GHz). However, the spectral shape suggests presence of at least two S = 5/2 components that differ particularly in *E/D*. The intense resonance at g_{eff} = 4.3 and the small signal at g_{eff} = 9.3 in the EPR spectrum of **Int I** can be assigned to a fully rhombic component with *E*/*D* = 1/3, whereas the peaks at g_{eff} = 8.6 and 5.4 indicate a subspectrum with smaller *E*/*D* < 0.15.[7] For the latter component, further signals are expected in the magnetic field range from 200 mT to 400 mT (see Figure S19, SI), but apparently these are blurred here and only a very shallow trough is observed instead. Such selective spectral broadening can be simulated by invoking pronounced strain in the *D* and *E* parameters (Δ*D* and Δ*E*,

respectively) as it would be expected for site-to-site disorder in the coordination environment of **Int I**. To constrict the possible ZFS values in the simulations, the magnitude and sign of *D* has been estimated from the temperature dependence of the EPR spectrum (see Figure S19, SI) to D = +0.4 cm^{-1} - +0.8 cm^{-1}. Reasonable agreement with the experiment could be obtained by assuming a superposition of just the two S = 5/2 as suggested above species with different *E/D* (Figure 4b). However, strong *D* and *E* strain had to be introduced to reproduce the characteristic spectral features.

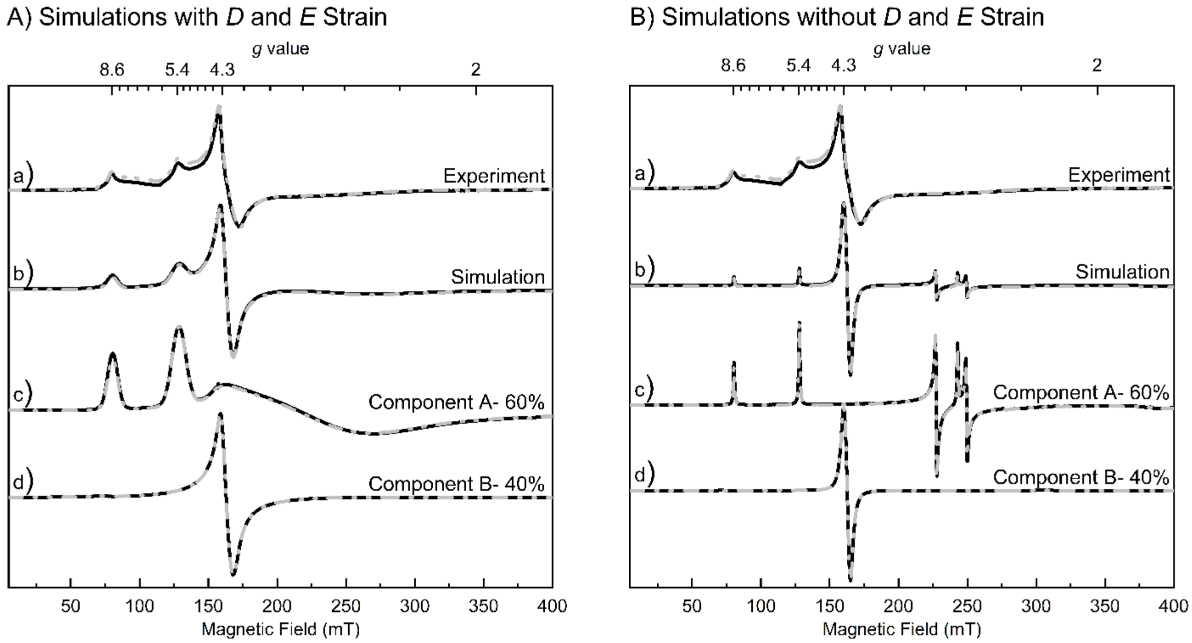

Figure S18. (A) Experimental (a) and simulated CW X-band EPR spectra (b) of **Int I** recorded at 10 K (black) and 70 K (grey). The deconvolution of the simulated spectrum is shown in (c) and (d) at 10 K (black) and 70 K (grey), respectively. Simulations were obtained using EasySpin[2] and following spin Hamiltonian parameters were used to simulate the X-band spectra component A (with approximately 60% abundance): g = 2, D = 0.4 cm^{-1}, E/D = 0.145, *D* and *E* strain of ΔD = 0.05 cm^{-1} and ΔE = 0.025 cm^{-1}, respectively and a Lorentzian line width broadening of 1 mT and component B (with approximately 40% abundance): g = 2, D = 0.4 cm^{-1}, E/D = 0.33, *D* and *E* strain of ΔD = 0.3 cm^{-1} and ΔE = 0.13 cm^{-1} and a Lorentzian line width broadening of 5 mT. B) Corresponding CW X-band EPR spectra simulated without *D* and *E* strain at 10 K (black) and 70 K (grey) are depicted for better visualization of all the high field peaks.

Determination of D

In the low-field limit, X-band EPR spectra of half integer states are independent of the absolute value of *D*. Determination of the latter, therefore, requires measurements at higher frequency and/or different temperatures. The minor changes in spectral shape of the X-band EPR spectrum of **Int I** at 10 K and 70 K (see Figure S19, left), provides an estimate for the size of *D*, via the temperature dependent Boltzmann population of the Kramers levels.

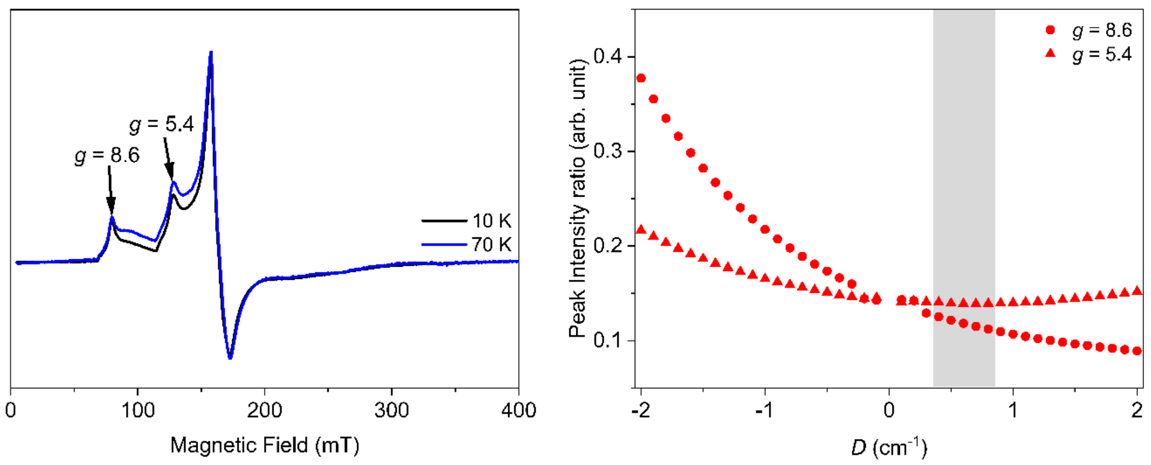

Figure S19. Experimental CW X-band EPR spectra of **Int I** recorded at 10 K (black) and 70 K (blue) shown on the left. Both spectra are normalized to the maximum peak intensity. The simulations on the right were obtained using spin Hamiltonian parameters of an S_t = 5/2 species with g = 2, D = -2 cm^{-1} to +2 cm^{-1} and E/D = 0.14 at 10 K and 70 K, respectively. The peak intensity ratios were calculated by dividing the signal intensity obtained at 70 K to the signal intensity obtained at 10 K for both g = 8.6 (●) and g = 5.4 (▲) peaks, respectively and plotted against *D* as show in red on the right. The simulated peak intensity ratios matched well against the experimental data for D = 0.4 cm^{-1} – 0.8 cm^{-1} as indicated by grey region.

To account for the sign and magnitude of *D*, the peak intensity ratios for g = 8.6 and g = 5.4 peaks were calculated by dividing the signal intensity at 70 K to the signal intensity at 10 K, respectively. The peak intensity ratios of 0.120 and 0.13 were derived experimentally

for g = 8.6 and g = 5.4 peaks, respectively and compared against the simulated data (Figure S19, right) obtained using S_t = 5/2, g = 2, E/D = 0.14 and D in the range of −2 to +2 cm^{-1}. The peak intensity ratios matched well for D in the range of 0.4 cm^{-1} to 0.8 cm^{-1} as indicated by grey region.

Mössbauer Spectroscopy:

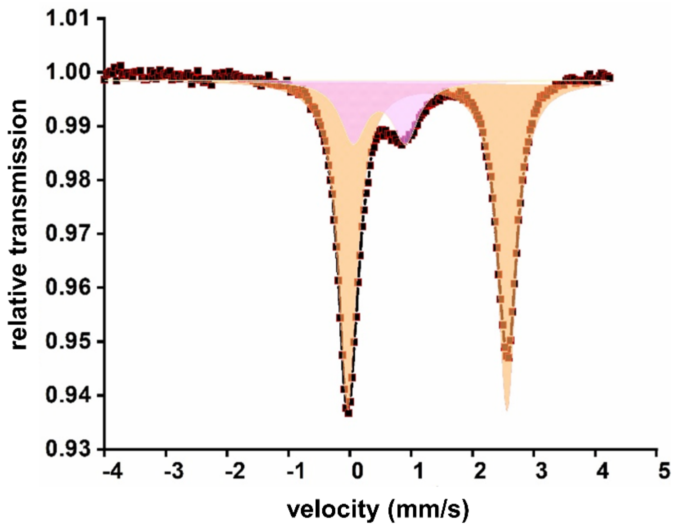

δ (mm/s)	ΔE_Q (mm/s)
1.26 (80%) (**1**)	2.60 (orange area)
0.48 (20%) (**1***)	0.85 (pink area)

Figure S20. Zero field Mössbauer spectrum of frozen solution of 2 mM samples of ^{57}Fe enriched **1** in toluene at 80 K.

From the Mössbauer spectra it is evident the majority of the starting material in solution is the complex **1** [$Fe(acac)_2(H_2O)_2$], a high spin Fe^{II} species (S_t = 2), with δ = 1.26 mm/s and ΔE_Q = 2.60 mm/s (orange area). The small amount of the other iron species (pink area) could be related to $Fe^{II}(acac)_2$ without the coordinated water, as evident when compared with the isolated Mössbauer spectrum of $Fe^{II}(acac)_2$ (**1***) (see Figure S23)

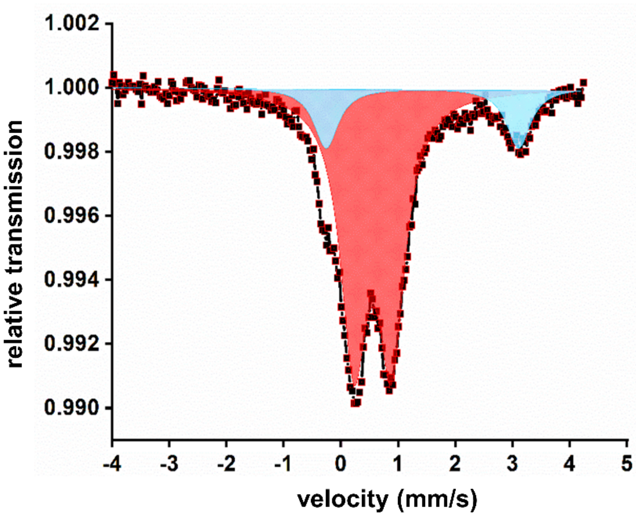

δ	***ΔE$_Q$***
0.55 (83%) (**Int I)**	0.66 (red area)
1.42 (17%) (**c1**)	3.36 (blue are)

Figure S21A. Zero field Mössbauer spectrum of frozen solution of **Int I** (2 mM sample of ^{57}Fe enriched **1** + $PivONH_3OTf$ frozen within 15 mins of mixing under anaerobic condition) in toluene at 80 K.

From the Mössbauer spectrum, it is evident the majority of the spectrum corresponds to a high spin Fe^{III} (S_t = 5/2) species with δ = 0.55 mm/s and ΔE_Q = 0.66 mm/s. This was also confirmed by EPR spectroscopy and the applied-field Mössbauer spectra shown below in part B of Figure S21. However, for the other component found in the reaction solution (c1, blue area) with δ = 1.42 mm/s and ΔE_Q = 3.36 mm/s we suggest erratic formation of another high spin ferrous species, during the sacrificial catalyst activation pathway, and the presence of several possible coordinating species in the reaction solution like H_2O, OTf^-, MeOH, $tBuCOO^-$ make it challenging to predict its unambiguous composition. This side product persists in the reaction mixture and does not take part in the subsequent reaction course as this was also found in the next step of the reaction.

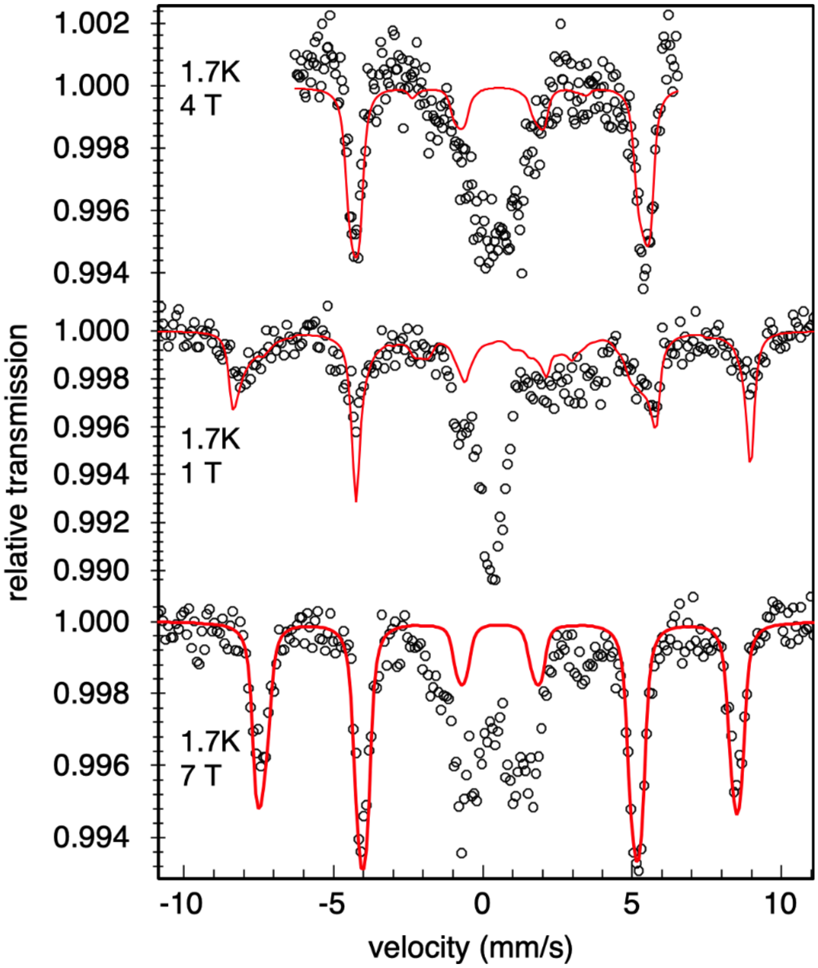

Figure S21B. Magnetic Mössbauer spectra of the 2 mM sample of ^{57}Fe-enriched **1** + PivONH$_3$OTf in toluene, frozen within 15 mins of mixing under anaerobic condition, as used for Figure S21A. The temperature was 1.7 K and fields of 1,4, and 7 T were applied perpendicular to the γ rays. The red lines represent a global spin Hamiltonian simulation with S = 5/2 for the major ferric high-spin component **Int I** found above (83%, δ = 0.56 mm/s and ΔE_Q = 0.66 mm/s). The ZFS parameters were taken form the major EPR subspectrum (D = 0.5 cm^{-1}, E/D = 0.15, g = 2), and an isotropic hyperfine coupling constant was found to be $A/g_N\beta_N$ = -22.26 T. The asymmetry parameter of the quadrupole interaction was close to its maximum, η = 0.95, and the electric feld gradient tensor (efg) was rotated by an Euler angle beta = 90°. The minor (17%) ferrous component found above was ignored.

The sensitivity of the magnetic Mössbauer spectra is not sufficient to resolve the distribution of subspectra and rhombic ZFS parameters found for **Int I** by EPR, but the

proper simulation of the field dependence of the major magnetic Mössbauer lines corroborates unambiguously that the main component of the experimental spectra, **Int I**, is a ferric high spin complex with spin S = 5/2 that is readily centered on iron.

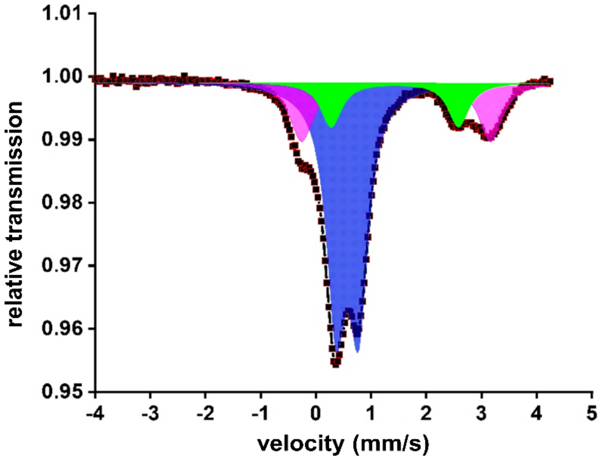

δ	ΔE_Q
0.57 (66%) (**Int II**)	0.40 (blue area)
1.42 (23%) (**c1**)	3.36 (pink area)
1.40 (11%) (**c2**)	2.28 (green area)

Figure S22A. Zero field Mössbauer spectrum of frozen solution of **Int II** (2 mM sample of ^{57}Fe enriched **1** + PivONH$_3$.OTf frozen within 90 min of mixing under anaerobic condition) in toluene at 80 K.

From the Mössbauer spectrum it is evident the majority of the spectrum corresponds to a high spin Fe^{III} (S = 5/2) species with δ = 0.57 mm/s and ΔE_Q = 0.40 mm/s. A decrease in the quadrupole splitting compared to **Int I**, suggests a change of coordination geometry around the iron center. Apparently, in this case the EPR silent nature of **Int II**, arises from spin coupling of the ferric central ion to a coordinated ligand radical. From quantum chemical calculations and other experimental data and literature precedence, **Int II** is best described as a high spin Fe^{III} (S = 5/2) species coupled to a nitrogen (NH) radical to form an iron imido species having overall integer spin S_t = 2, which is corroborated below in part B. The other components found in the reaction solution with δ = 1.42 mm/s and ΔE_Q

= 3.36 mm/s (c1, pink area) and with δ = 1.43 mm/s and ΔE_Q = 2.28 mm/s (c2, green area) though not assigned unambiguously, are likely some high spin Fe^{II} species formed in the reaction solution, as a result of complex multistep reaction pathways involved. (see catalyst activation section in the manuscript for the multistep pathway)

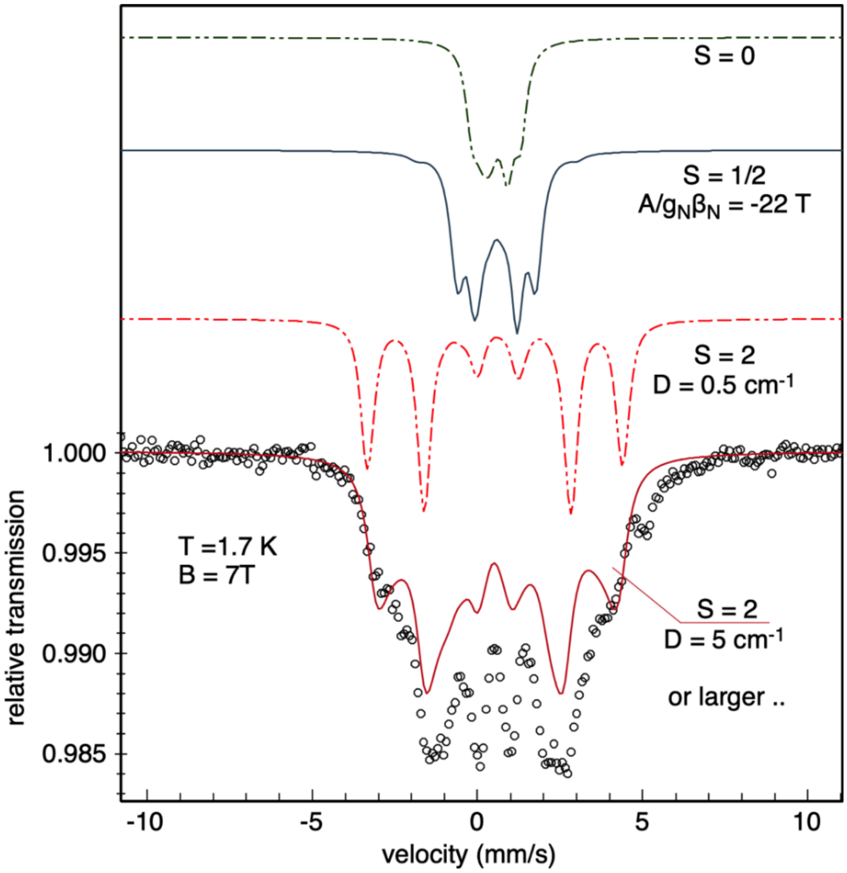

Figure S22B. Magnetic Mössbauer spectrum of the 2mM sample of ^{57}Fe-enriched **1** + $PivONH_3OTf$ in toluene, frozen within 90 mins of mixing under anaerobic condition, as used above for Figure S22A. The spectrum was measured at 1.7 K with a field of 7 T applied perpendicular to the γ rays. The solid red line is a preliminary simulation with total spin S_t = 2 for the main subspectrum, shown above in blue, which is assigned to intermediate **Int II** (66%, δ = 0.57 mm/s, ΔE_Q = 0.40 mm/s). The axial ZFS parameter had to be taken as $D \geq 5$ cm^{-1} (with E/D = 0.15, g = 2) to reproduce roughly the magnetic splitting of the spectrum with a reasonable hyperfine coupling tensor, $A_i/g_N\beta_N$ = (-8, -14.8, -20). The asymmetry parameter was fixed to η = 0.9, and the efg was rotated by Euler angles, alpha = 90, beta = -10°. The minor (23% + 11%) ferrous components found above were ignored.

The significant magnetic splitting of the experimental spectrum reveals the presence of a substancial internal magnetic field at iron. This excludes any alternative, hypotheticallly possible, interpretation of isomer shift and quadrupole splitting of **Int II** by adopting a ferrous low-spin compound with S = 0 (see simulation, dashed top trace). Also, a ferric low-spin complex with a coordinated radical, exhibiting total spin S_t = 0, is ruled out, as well as a ferrous low-spin complex with a radical spin (S_t = 1/2, solid black line).

The S_t = 2 simulation (red line overlayed with the data) is by no means unique, but since the trace of the A-tensor (≈ -18 T) is in the range expected for ferric high spin complexes, it strongyl suggests total spin S_t = 2 for **Int II**, due to antiferromagnetic coupling of a ferric high spin iron center (S = 5/2) with a coordinated amin radical (S* = 1/2). The broadened magnetic Mössbauer reveals further that the magnetic anisotropy due to ZFS of **Int II** must be significantly higher than that of **Int I**, which was found by EPR to be of the order 0.5 cm^{-1} (see dotted red line). This difference reflects again the different coordination spheres of **Int I** and **Int II**.

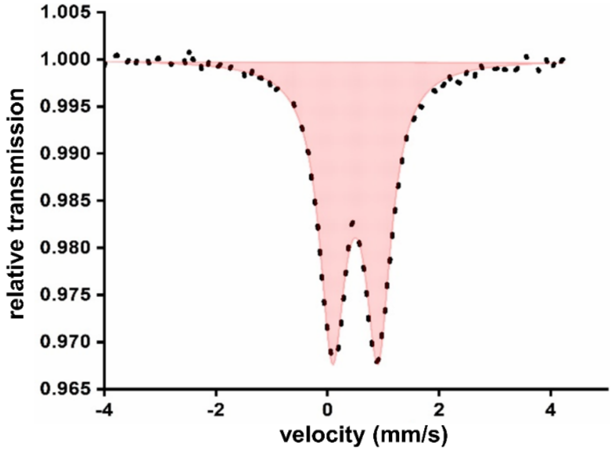

δ	ΔE_Q
0.48	0.82 (red area)

Figure S23. Zero field Mössbauer spectrum of a solid sample of anhydrous $Fe(acac)_2$ (**1***) in dimeric form at 80 K.

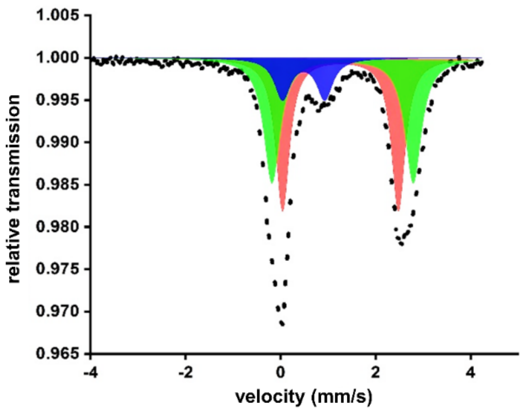

δ	ΔE_Q
1.26 (44%)	2.44 (red area)
1.30 (42%)	2.97 (green area)
0.48 (14%)	0.83 (blue area)

Figure S24. Zero field Mössbauer spectrum of frozen solution of $Fe(acac)_2$ (20 mM in toluene, natural abundance of ^{57}Fe) at 80 K.

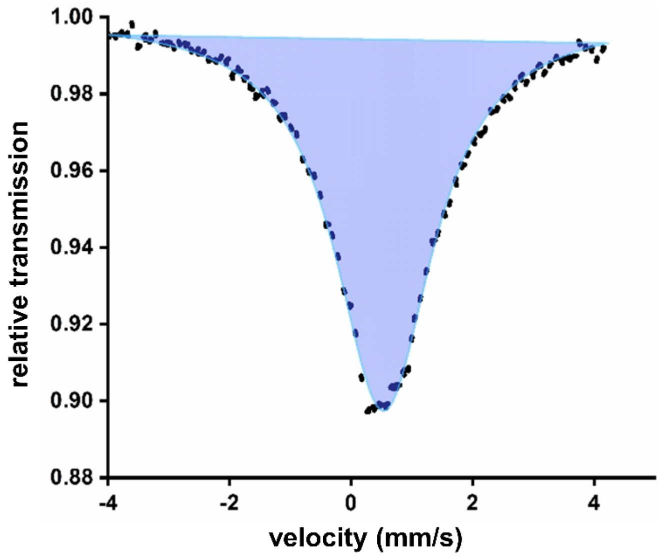

δ	ΔE_Q
0.54	0.0

Figure S25. Zero field Mössbauer spectrum of solid sample of $Fe(acac)_3$ at 80 K.

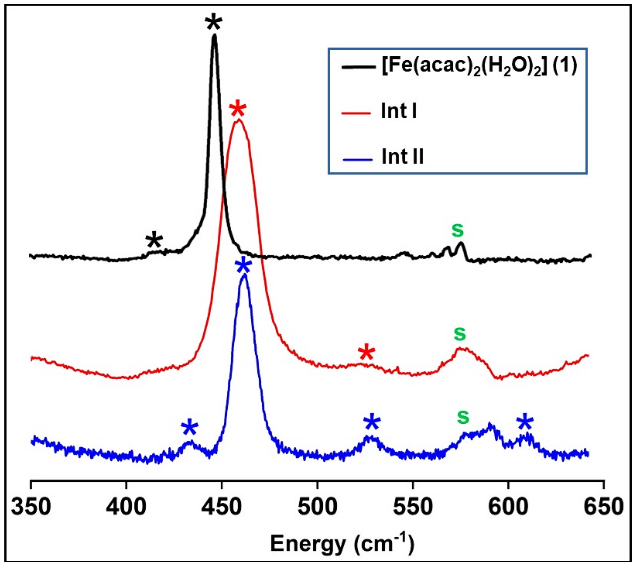

Figure S26. Resonance Raman spectra of frozen dichloromethane solution of **1** (black) and **Int I** (red) at 491 nm laser excitation and **Int II** (blue) at 660 nm excitation (* marked peaks are from precursor and intermediates, s denote solvent peaks). Experimental conditions: 1 mM solution 100 K, 100 mW, 500μm slit. For full spectral band assignments, see computational section Table S13, SI.

Nuclear Vibrational Resonance Spectroscopy (NRVS)

For NRVS measurements of $[Fe(acac)_2(H_2O)_2]$ (**1**) and the *in situ* formed intermediates (**Int I** and **Int II**) during reaction of **1** with an aminating agent $PivONH_3OTf$, the $[Fe(acac)_2(H_2O)_2]$ (**1**) precursor (20 millimolar) was labelled with ^{57}Fe. A sample of the precursor material was measured, as well as samples that were incubated with the aminating agent (3 equiv.) for 15 min and 90 min, conditions known to generate predominantly **Int I** and **Int II,** respectively, as established from the previous spectroscopic studies (UV-vis, EPR, Mossbauer, XAS, rR). Samples were quenched by freezing in liquid nitrogen before measuring the NRVS. The significant NRVS peaks of reaction components have been discussed in the main manuscript with the complete spectrum in Figure S27 and Table S9.

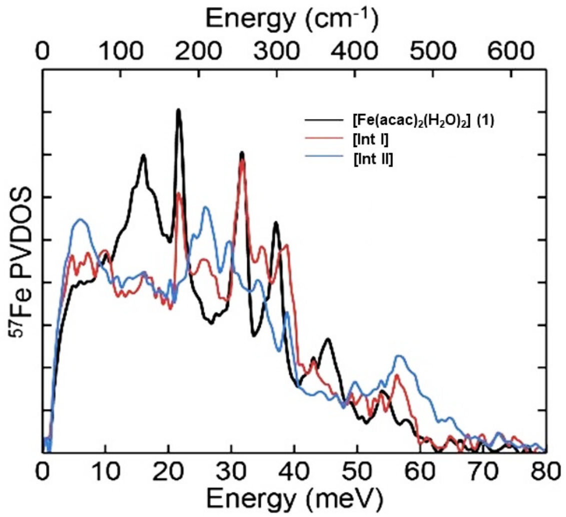

Figure S27. Experimental ^{57}Fe NRVS spectra of $^{57}Fe(acac)_2(H_2O)_2$ (**1**) precursor (black) (20 mM) and the reaction intermediates observed after 15 min **Int I** (red) and 90 min **Int II** (blue) of reaction of **1** with the aminating agent $PivONH_3OTf$ in frozen toluene.

Table S9. ^{57}Fe NRVS spectra of $^{57}Fe(acac)_2(H_2O)_2$ precursor **1** and the reaction intermediates observed after 15 min (**Int I**) and 90 min (**Int II**) of reaction with the aminating agent $PivONH_3OTf$.

**Energy (cm^{-1}) for Precursor 1**	**Energy (cm^{-1}) Int I (15 min data)**	**Energy (cm^{-1}) Int II (90 min data)**
130	-	-
174	174	-
-	207	207
256	256	241
-	280	280
299	313	313
366	393	-
440	458	462

COMPUTATIONAL SECTION

All calculations were carried out with the ORCA program package[8] version 4.2.1. Density functional theory was used with the B3LYP functional[9-12] together with Grimme's D3 dispersion correction[13] with Becke-Johnson damping.[14] The Ahlrichs def2-TZVP basis set[15] was used. To speed up the calculations, the resolution of identity[16] was invoked in the Split-RI-J variant.[17] In addition, the RIJCOSX approximation[18] to the exchange integrals was use used together with the corresponding auxiliary basis set.[19]Equilibrium geometries were proven to be real minima on the PES by the absence of imaginary frequencies, while transition states were proven to be first-order saddle points on the PES by the presence of one imaginary frequency. Free energies were calculated using the Quasi-RRHO approach[20] from Grimme. For geometry optimizations implicit solvation was included via the C-PCM model[21] (Toluene) together with the gaussian charge scheme.[22-23] Transition states were partially found with the help of the climbing image NEB method[24-26]. Excited states were calculated within the framework of time-dependent DFT.[27-28] Fine DFT and COSX Grids were used in all cases (Grid7 and GridX7 in ORCA notation). To facilitate the chemical analysis, natural transition orbitals[29] were computed. X-Ray absorption spectra were calculated with TD-DFT as well, but only the Fe 1s-electrons were allowed for excitation. All virtual orbitals were chosen as potential acceptor orbitals. For core spectroscopy scalar-relativistic effects were taken into account by ZORA[30-31] in combination with the corresponding uncontracted basis sets.[32] The deconvolution of the experimental absorption spectrum was carried out by performing a least-square fit of gaussians to the spectrum. One gaussian for each resolvable feature was used. The fitting was done with the help of the ORCA ASA program.[33-34] The ORCA ASA program was also used for the simulation of Resonance Raman spectra. Natural charges and natural spin densities were obtained within the Natural Bond Orbital (NBO) scheme[35] using the NBO 7.0 program.[36] The calculation of Mössbauer isomer shifts δ requires the electron density at the nucleus ρ_0:

$$\delta = \alpha(\rho_0 - C) + \beta$$

The fitting parameters α, β and C depend on the density functional and the basis set and can be found in literature.[37] For the calculations of Mössbauer parameters the CP(PPP) basis set[38] was invoked for Iron.

For the precursor and both postulated reaction intermediates possible conformers were obtained by performing conformer sampling with CREST[39] in combination with GFN2-xTB.[40-41] DFT geometry optimizations were carried out on all found conformers afterwards. For the simulated absorption spectra, a linewidth of 3500 cm^{-1} was used. For the simulated resonance Raman spectra, a linewidth of 10 cm^{-1}, for the NRVS spectra a linewidth of 15 cm^{-1} and for the simulated XAS spectra a linewidth of 0.8 eV was used.

[Fe(acac)$_2$(H$_2$O)$_2$] (1): The precursor

Energetics of the Spin States and Geometric Models for 1:

For the precursor [Fe(acac)$_2$(H$_2$O)$_2$] (**1**), the experimental Mössbauer parameters having an isomer shift (δ) value of 1.26 mm/s and a quadrupole splitting (ΔE_Q) value of 2.60 mm/s (Figure 4 top), along with the lower energy rising edge feature observed in HERFD-XAS (Figure 5a) have shown that iron is likely to be in the Fe(II) high spin state. The system's integer total spin was already confirmed by the absence of EPR signals in the perpendicular mode (Figure 3). From the relative free energy calculations, for the precursor [Fe(acac)$_2$(H$_2$O)$_2$] (1), the quintet state (S_t = 2; 0 kcal/mol) is clearly favored over the triplet (S_t = 1; 15.35 kcal/mol) and singlet (S_t = 0; 28.14 kcal/mol) state (Table S10). To directly compare the possible geometric features of the precursor complex 1, both *cis* and *trans* isomers were considered (Figure S28). Free energy calculations slightly favor the trans geometry over the *cis* one by ~1 kcal/mol. However, such a small energy difference is certainly within the uncertainty of the computational protocol.

Table S10. Relative free energies for different spin multiplicities of **1**. In each case the geometry was optimized in order to take the breathing mode of Fe into account.

Total Spin (S_t)	Spin multiplicity ($2S_t$+1)	Rel. DFT energy (kcal/mol)
0	1	28.138
1	3	15.354
2	5	0.000

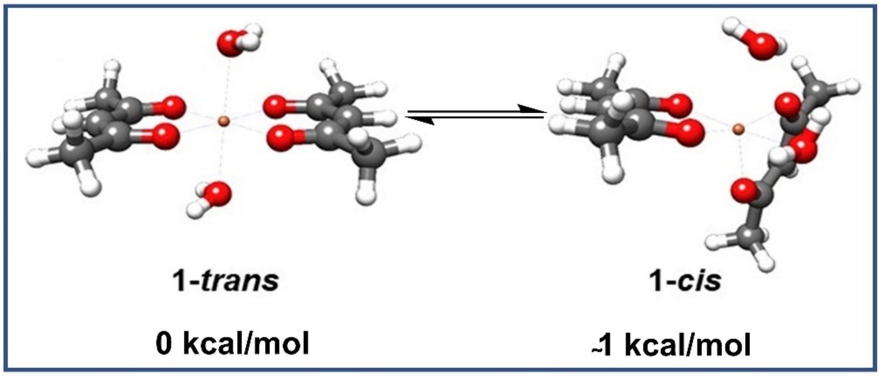

Figure S28. (a) Computed Geometries of [Fe(acac)$_2$(H$_2$O)$_2$] (1): 1-*trans* and 1-*cis* isomer. Calculations were carried out at the B3LYP-D3/def2-TZVP level.

Spectroscopic features of 1

A) Absorption Spectroscopy (ABS) of 1: As was evident from the electronic structure of 1-*cis* and 1-*trans* isomers, the lower energy MLCT transition is the most prominent feature to be expected in the absorption spectrum of **1**. The calculated absorption spectra for both the **1**-*cis* and **1**-*trans* isomer (see SI, Figure S29 and Table S11) do not differ significantly from each other and both match reasonably well with the experimental UV-Vis absorption spectrum (Figure 1). Hence, no clear indication of the conformation (*cis* or *trans*) can be concluded based on UV-Vis absorption spectroscopy. The *cis*-isomer has a slightly better agreement than the *trans*-isomer with respect to the first excitation (both in terms of excitation energy and oscillator strength, Table S11). The high-energy range >35,000 cm^{-1} of the computed spectrum consists of many different excitations leading to only two observable features in the experimental spectrum (Figure 1 and Figure S2).

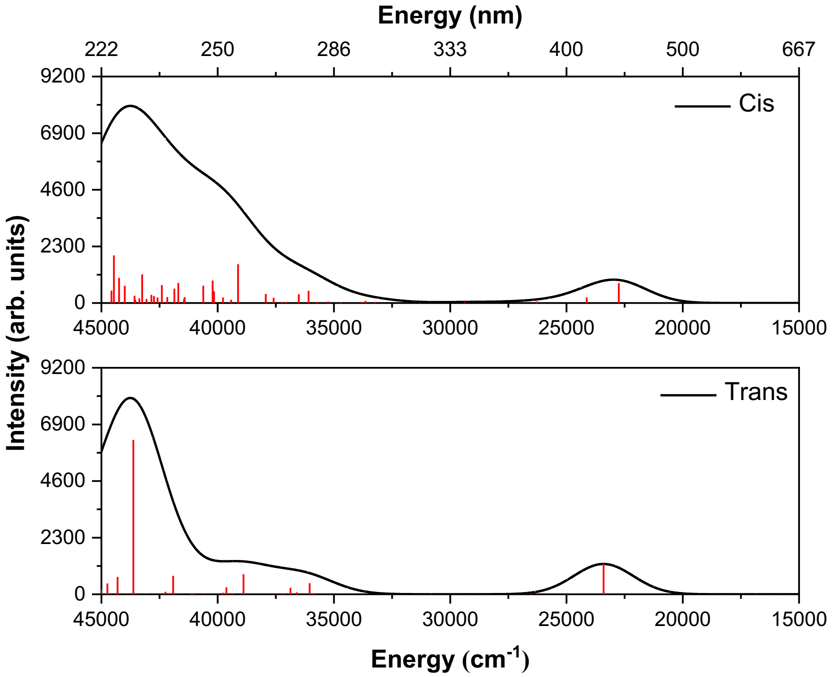

Figure S29. Computed absorption spectrum of the precursor (**1**). Top: *trans*-isomer. Bottom: *cis*-Isomer. The intensities were scaled to fit the experimental extinction coefficients.

In Table S11 below, we compare the calculated absorption energies and oscillator strengths with those obtained from the deconvolution of the experimental spectrum.

Table S11. Comparison between theoretical and experimental absorption parameters for the precursor **1**.

Excitation energy cm^{-1} (1)			Oscillator strength (1)		
**1**-Exp.	**1**-*cis*	**1**-*trans*	**1**-Exp.	**1**-*cis*	**1**-*trans*
22806	23083	23410	0.044	0.047	0.069
28459	-	-	0.021	-	-
35702	39014	38598	0.173	0.215	0.188
41837	43881	43894	0.274	0.424	0.462

NB: The presented excitation energies and oscillator strengths are obtained from a gaussian fitting procedure.

B) Mössbauer Parameters of 1: In the next step, the possible *cis* and *trans* isomers of **1** were evaluated by comparing the experimental and calculated Mössbauer parameters (Table S12). In evaluating the calculated Mössbauer parameters, we focus on the isomer shift δ, which is proportional to the charge density at the nucleus ρ_0, and the quadrupole splitting, derived from the electric field gradient at the iron nucleus and can be calculated using equation 2.[42]

$$\delta = \alpha(\rho_0 - C) + \beta \ldots\ldots (2)$$

The fitting parameters α, β and C depend on the density functional and the basis set and can be found in literature.[37] The standard deviation for the linear fitting of the parameters α, β and C is around 0.1 mm/s.[37] From the table (Table S12), it is evident that for both the ***1-cis*** and ***1-trans*** isomers the isomer shift values are lower than the experimentally observed isomer shift. However, the difference in the calculated isomer shift values between the ***1-cis*** and ***1-trans*** isomer is only 0.021 mm/s which is small compared to the error tolerance. Hence from the Mössbauer parameters clear distinction between the ***1-cis*** or ***1-trans*** conformer could not be made. Interestingly, the calculated quadrupole splitting is in very good agreement with experiment for both the isomers, with a slightly better agreement for the ***1-cis*** isomer.

Table S12. Calculated Mössbauer parameters for the different geometric isomers of 1 compared to the experimental result.

Mössbauer parameter	**1**-*cis* isomer	**1**-*trans* isomer	**1**-Exp.
δ (mm/s)	1.047	1.026	1.26
ΔE_Q (mm/s)	2.591	2.473	2.6

C) Resonance Raman (rR) Spectroscopy for 1: Computed resonance Raman spectra are useful for the characterization of excitations and normal modes. It relies on the enhancement of selected vibrational modes depending on the nature of the electronic

transition, so we selected the low energy MLCT transition for **1** as the electronic excitation. For the computed resonance Raman spectra (Figure S30, Table S13, SI), a vertical excitation energy of 22,749 cm^{-1} and 23,407 cm^{-1} was used for the **1**-*cis* and the 1-*trans* isomers respectively to fit the calculated excitation energies for the MLCT excitation (Fe-t_{2g} to acac-π*) (Figure S33, SI). Though the computed resonance Raman spectra for the 1-*cis* and 1-*trans* isomers do not differ significantly, there is good agreement between the experimentally observed excitations and the calculated ones (Figure S26 and Figure S30, Table S13, SI). For 1, the electronic transition corresponding to MLCT of the Fe^{II} d orbital to an empty π* orbital of the acac ligand, gives rise to the most prominent and intense peak in the resonance Raman spectrum, which is assigned to the Fe-O stretching mode. The experimentally observed Fe-O symmetric stretching mode for **1** was observed at 450 cm^{-1} with a laser excitation of 491 nm (20,367 cm^{-1}). For the symmetric Fe-O stretching mode, the calculated excitation occurs at lower energy for both isomers (420 cm^{-1} for **1**-*cis* and 417 cm^{-1} for **1**-*trans*) compared to the experimental observed rR stretch. Besides this, the experimentally observed excitation at 265 cm^{-1} assigned to as the δ(Fe-O_{acac}) were also well reproduced in the calculated spectra for both the isomers (Figure S30, Table S13, SI). The electronic structure calculations of **1** have revealed the electron density of the acac ligand is extensively delocalized and hence excitations involving the acac HOMO enhance C-H deformation vibrations of the methyl groups over the acac ligand backbone. This was also evident from the experiment, where the methyl C-CH_3 stretches (1284 cm^{-1}) as well as C-O carbonyl stretches (1625 cm^{-1}) from the acac ligand were observed in the experimental rR spectrum (see SI Table S13). Overall, the experimental rR spectrum agrees well with the computed spectrum (Figure S33, Table S13). Thus, taken together, the close similarity of the computed resonance Raman spectra of the **1**-*cis* and **1**-*trans* isomers, along with their correlation to the experimentally observed spectrum, does not allow clear assignment about the geometric isomer of **1** based on resonance Raman spectroscopy.

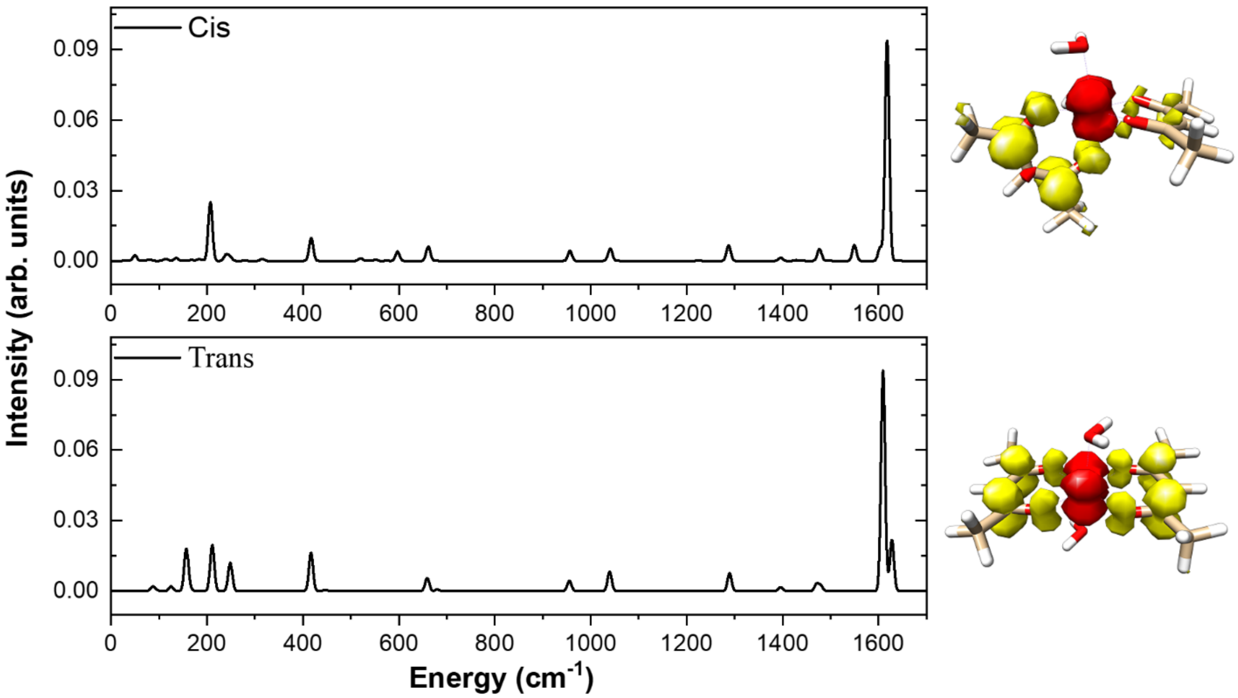

Figure S30. Simulated resonance Raman spectra for the precursor (**1**): Top: *cis*-isomer. Vertical excitation energy is 22749 cm^{-1}. Bottom: *trans*-isomer. Vertical excitation energy is 23407 cm^{-1}. The excitation energies were chosen to fit the calculated excitation energies for the MLCT excitation (Fe-t_{2g} to acac-π*). The difference density plots are shown on the right of the spectrum (donor region in red, acceptor region in yellow).

Table S13. Selected computed and experimental vibrational frequencies for the precursor **1**. All energies are in cm^{-1}.

Assignment	**1**-*cis*-isomer	**1**-*trans*-isomer	**1**-Exp.
δ(Fe-O_{acac})	256	262	265
*υ*s(Fe-O_{acac})	420	417	450
υ(C-CH_3)	1289	1290	1284
*υ*s(C-O)	1618	1610	1625

D) NRVS of 1: Since NRVS gives results analogous to rR, but with band intensity of the vibrational modes that is directly proportional to the amount of ^{57}Fe motion, the symmetric Fe-O stretch of the computed NRVS matches well with the computed rR results for both the isomers of **1** at around 417-420 cm^{-1} (Figure S31, Table S14, SI). Computed NRVS of **1** revealed quite intense deformational modes below 300 cm^{-1} that describe the iron atom moving 'in between' the Fe-O bonds (Figure S31, SI). For the 1-*trans* isomer, this peak overlaps with the δ(Fe-O$_{acac}$) vibrational mode resulting in a broadened peak in the 260-300 cm^{-1} region (Figure S31, Table S14). However, the computed NRVS for the **1**-*cis* isomer exhibits separate excitations at around 279 cm^{-1} for the δ(Fe-O$_{acac}$) mode and the mode around 300 cm^{-1} depicts the movement of iron 'in between' the Fe-O bonds (Figure S31, Table S14). From the experimental NRVS results for **1** (Figure S27, Table S9), the symmetric Fe-O stretch appears at higher energy (440 cm^{-1}) compared to the computed value, which was also evident from the rR calculation (see previous section). Taking a closer look at the experimental NRVS for **1** (Figure S27 and Table S9 and Table S14), the 256 cm^{-1} stretch could be assigned to the δ(Fe-O$_{acac}$) vibration and the peak around 299 cm^{-1} corresponds to the movement of iron 'in between' the Fe-O bonds. This experimental NRVS gives better agreement with the **1**-*cis* isomer compared to the **1**-*trans* (Figure S31, Table S14).

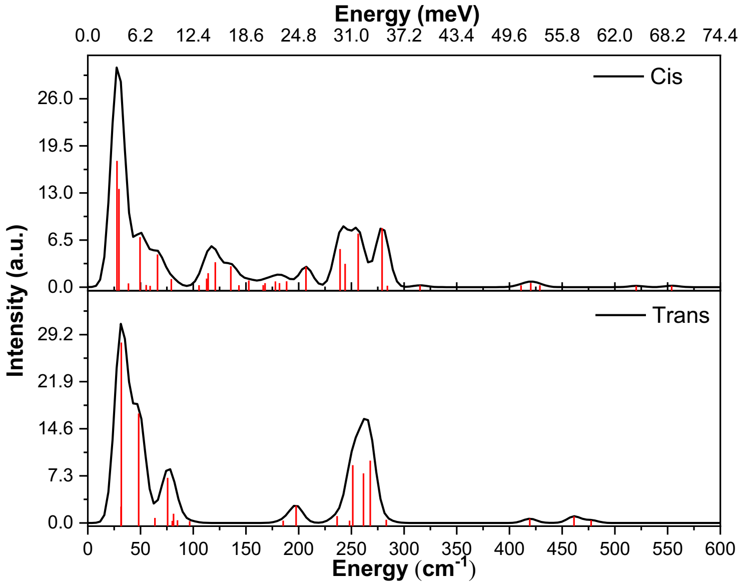

Figure S31. Simulated NRVS spectra for the precursor **1**: Top: *Cis*-isomer. Bottom: *Trans*-isomer.

Table S14. Selected computed and experimental vibrational frequencies of NRVS spectrum for the precursor **1**. All energies are in cm^{-1}

Assignment	**1**-*cis*-isomer	**1**-t*rans*-isomer	**1**-Exp.
δ(Fe-O_{acac})	256/279	262/268	256/300
us(Fe-O_{acac})	420	419	440

E) K$\beta_{1,3}$ HERFD XAS of 1: To complement the K$\beta_{1,3}$ HERFD XAS experimental results, we also pursued a systematic computational study of the TDDFT-computed pre-edges of **1** for both the **1**-*cis* and **1**-*trans* model systems (Figure S32, SI). The excitations occur into the singly occupied d-manifold, with a clear energetic separation between the nonbonding t_{2g}-orbitals and antibonding e_g-orbitals. The energy difference between these two peaks reflects the change in ligand field splitting, with the **1**-*cis* isomer having a slightly smaller splitting of the two peaks (ΔE = 1.1 eV) compared to the **1**-*trans* isomer (ΔE = 1.4 eV). The experimental XAS pre-edge of **1** shows a splitting of ~1.3 eV which consists of two grouped transitions, consistent with a distorted local octahedral O_h geometry around iron (Figure S32). Both the **1**-*cis* and **1**-*trans* models show a similar shape (Figure S32). To assess this more quantitatively, the calculated area, as well as the intensity weighted average energy (IWAE), is compared to the experiment in Table S15. Looking at the calculated IWAEs, both models agree reasonably well with experiment, with a better agreement for the **1**-*trans* conformer in terms of peak area. Moreover, the **1**-*trans* model also has better visual agreement to the experiment, in terms of intensity distribution and peak splitting.

Table S15. XAS parameters for both isomers of the precursor **1** and the experimental result. ΔE describes the difference between the two peak maxima in eV.

Entry	**1**-*cis*	**1**-*trans*	**1**-Exp.
IWAE (eV)	7112.84	7112.74	7112.95
Area/Amplitude	42	19	25
ΔE (eV)	1.1	1.4	1.3

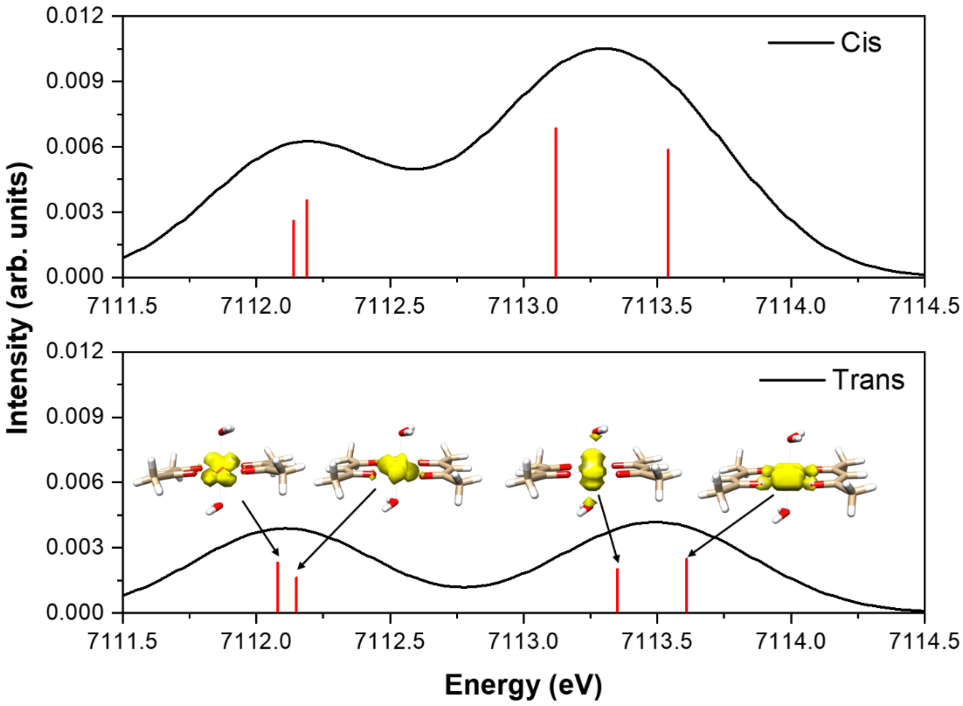

Figure S32. Simulated pre-edge region of HERFD-X-Ray absorption spectra for the precursor (**1**): Top: *cis*-isomer. Bottom: *trans*-isomer. To match the experimental spectra, the simulated spectra were shifted by 23.36 eV and scaled by 0.022. For the *trans*-isomer the calculated difference density plots for the respective excitations were added. The linewidth for the gaussian broadening was 0.8 eV. Only excitations of the β-electrons were allowed.

Electronic structure of 1:

To obtain more insight into the electronic structure quasi-restricted orbitals are used to assemble the orbital representation of the electronic structure. The MO diagrams derived from DFT calculations show a splitting pattern within the metal-d derived MOs that is broadly consistent with a strongly distorted octahedral ligand field with recognizable sets of metal t_{2g} and e_g parentage. The high-spin d^6 electron configuration leads to the lowest (d_{xy}) orbital to be doubly occupied while the other metal-d derived orbitals remain singly occupied. The covalent mixing is very limited as indicated by the average ligand characters of 5.9% (t_{2g} set) and 10.5% (e_g set). The highest doubly occupied ligand

orbitals are of acac- π character and the lowest unoccupied orbitals feature acac- π* orbitals (Figure S33).

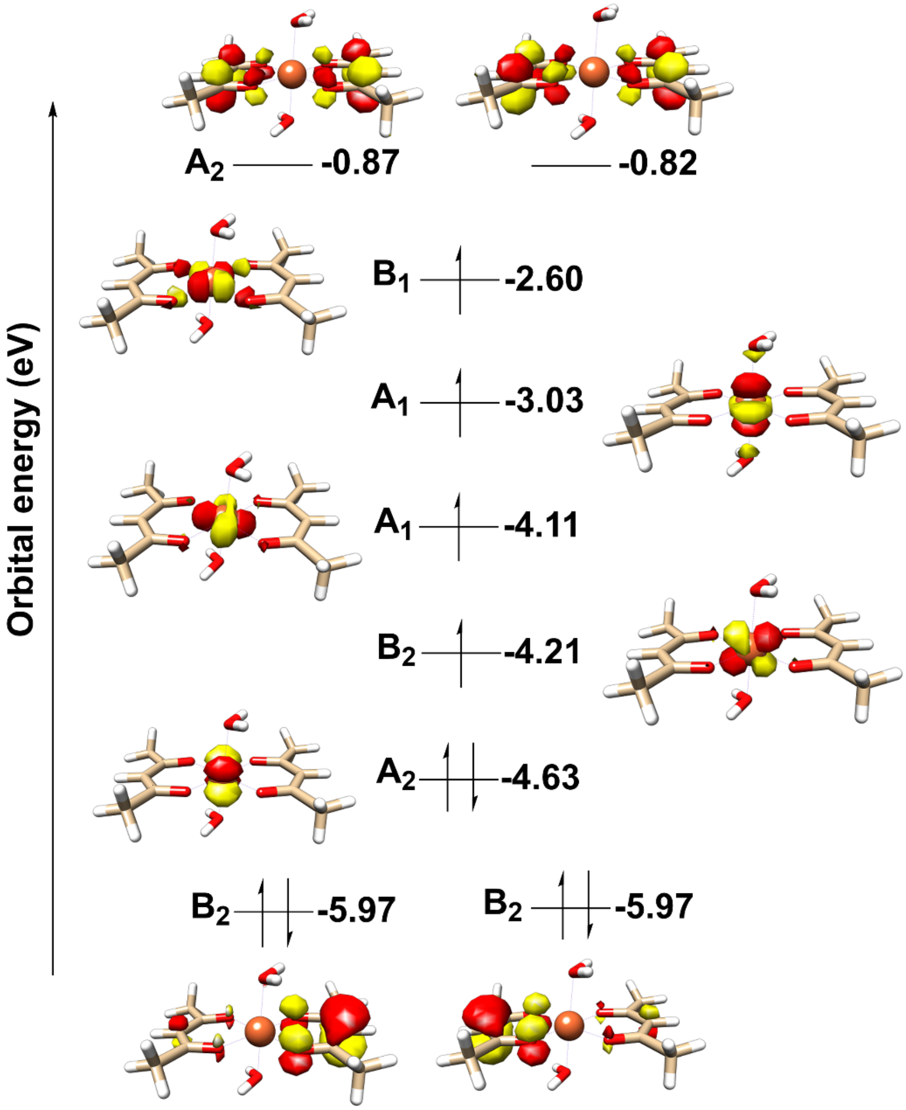

Figure S33. Electronic structure of the precursor **1** for **1**-*trans* model based on quasi-restricted orbitals (QROs[43]). Approximate symmetry labels refer to the C_{2v} point group. Calculations were carried out at the B3LYP-D3/def2-TZVP level.

Preferred model for precursor 1: The calculated spectroscopic parameters like UV-Vis ABS, Mössbauer Spectroscopy, Resonance Raman, NRVS and $K\beta_{1,3}$ HERFD XAS for the

1-*cis* and **1**-*trans* isomer have been compared with the experimental results. (Figure S34) For both computed isomers of **1**, the calculated absorption spectra result in a low energy feature at ~23000-23400 cm^{-1} (Figure S29 and Table S11) that matches the experimental data (~22806 cm^{-1}, Figure 1 and S2 and Table S1). This feature is assigned as an MLCT excitation due to the doubly low-lying t_{2g} orbital (Figure S33). These electronic transitions give rise to the most prominent and intense peaks in the experimental resonance Raman spectrum of **1** and can be well correlated with the calculated rR spectra for the **1**-*cis* and **1**-*trans* isomers (Figure S34). Comparison of experimental NRVS result with the computed values for **1**-*cis* and **1**-*trans* isomers show a slightly better agreement for the **1**-*cis* isomer (Figure 34). Experimental Mössbauer parameters of **1** (δ = 1.26 mm/s and ΔE_Q = 2.6 mm/s) agree reasonably well with the computed values of both the **1**-*cis* (δ = 1.05 mm/s and ΔE_Q = 2.6 mm/s) and **1**-*trans* (δ = 1.03 mm/s and ΔE_Q = 2.5 mm/s) models (Figure S34). In the calculated $K\beta_{1,3}$ HERFD-XAS pre-edge region of **1**, the **1**-*trans* model have better visual agreement to the experiment (Figure S34), in terms of intensity distribution and peak splitting, as well as to the experimental peak area or amplitude (42 for **1**-*cis vs* 19 for **1**-*trans*; 1-Exp = 25) (Figure S34). Hence, from the correlation of the different experimental spectroscopic results with the computational calculations, it is evident that no clear assignment of the geometric model for 1 (**1**-*cis* or **1**-*trans*) could be possible. Though the **1**-*trans* isomer may be slightly thermodynamically favored and more probable from XAS studies, it is plausible that both the conformers might exist in equilibrium in solution under the experimental conditions (Figure S34).

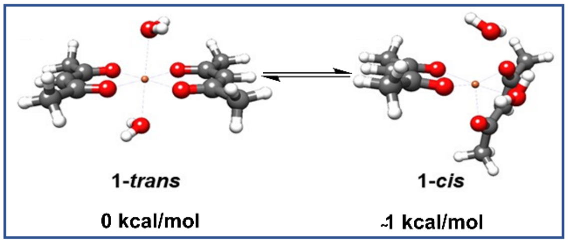

(a)

Mössbauer parameter	1-*cis*	1-*trans*	1-Exp.
δ (mm/s)	1.047	1.026	1.26
ΔE_Q (mm/s)	2.591	2.473	2.6

(b)

XAS Entry	1-*cis*	1-*trans*	1-Exp.
IWAE (eV)	7112.84	7112.74	7112.95
Area	21	19	25
ΔE (eV)	1.1	1.4	1.3

(c)

rR (cm^{-1})	1-*cis*	1-*trans*	1-Exp.
δ(Fe-O$_{acac}$) (bend)	256	262	265
υs(Fe-O$_{acac}$) (str)	420	417	450
υs(C-CH$_3$) (str)	1289	1290	1284
υs(C-O) (str)	1618	1610	1625

(d)

NRVS (cm^{-1})	1-*cis*	1-*trans*	1-Exp.
δ(Fe-O$_{acac}$) (bend)	256/279	262/268	256/300
υs(Fe-O$_{acac}$) (str)	420	419	440

Figure S34. Overview of the correlation of experimental and calculated spectroscopic parameters of **1** for (a) Mössbauer, (b) HERFD-XAS, (c) rR (d) NRVS. Calculations were carried out at the B3LYP-D3/def2-TZVP level.

Fe-*N*-acyloxy species: [Fe^{III}(acac)$_2$(NH-OPiv)] (Int I)

Energetics of spin states of Int I

Int I is a non-integer spin system. The relative DFT energies of the doublet, quartet and sextet state are presented in Table S16.

Table S16. Relative free energies for different spin multiplicities for **Int I**. In each case the geometry was optimized in order to take the breathing mode of Fe into account.

Total Spin (S_t)	Spin multiplicity ($2S_t$+1)	Rel. DFT energy (kcal/mol)
1/2	2	12.764
3/2	4	10.370
5/2	6	0.000

Spectroscopic properties of Int I

A) Absorption Spectroscopy (ABS) of Int I: As was evident from the electronic structure of **Int I**, the lower energy LMCT transition is the most prominent feature to be expected in the optical absorption spectrum. In the experimental absorption spectrum this LMCT occurs at around 480 nm exhibiting a red shift, compared to the MLCT chromophore of precursor **1** (437 nm) (Figure 1 and Figure S2). From the calculated optical absorption spectra for both the 6 and 5 coordinate models of **Int I** with Fe(N+O) and FeN binding respectively (Figure S35, Table S17, SI), the 6-coordinate model with the iron-nitrogen and iron-keto oxygen binding is clearly favored especially for the low energy excitation with a red shift of the band (Figure S35, Table S17, SI).

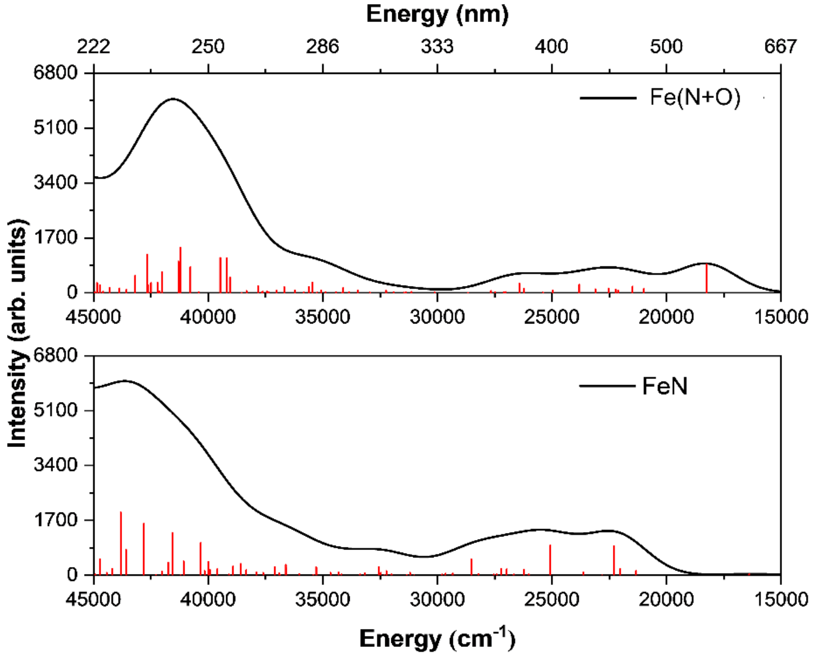

Figure S35. Computed absorption spectrum of **Int I**. Top: Fe(N+O) binding mode. Bottom: Fe-N binding mode. The intensities were are scaled to fit the experimental extinction coefficients.

Table S17. Comparison between theoretical and experimental absorption parameters for **Int I**.

Excitation energy cm^{-1} (**Int I**)			Oscillator strength (**Int I**)		
Exp.	Fe(N+O)	FeN	Exp	Fe(N+O)	FeN
20656	18051, 21918	22651	0.040	0.043, 0.065	0.086
28018	25980	2650	0.009	0.043	0.082
33819	38501	36695	0.066	0.163	0.226
41070	41748	40594, 44213	0.180	0.562	0.192, 0.296

NB: The presented excitation energies and oscillator strengths are obtained from a gaussian fitting procedure.

B) Mössbauer Parameters of Int I: Mössbauer parameters of the 6-coordinate vs 5-coordinate models of **Int I** are computed to compare with the experimental result. (Table S16). From the isomer shift values, it is clear that both of the models show an Fe(**III**) high spin species, with a better agreement of the experimental isomer shift for the 6-coordinate Fe(N+O) model. The quadrupole splittings are in moderate agreement with experiment, however, it is known from previous studies that calculated isomer shifts tend to be more reliable than the calculated quadrupole splittings.[44-48]

Table S18. Comparison of the Mössbauer parameters for the computed 6-coordinate Fe(N+O) and 5-coordinate FeN conformer of **Int I** with the experimental results.

Mössbauer parameter	Fe(N+O) **Int I**	FeN **Int I**	Exp. **Int I**
δ (mm/s)	0.609	0.518	0.55
ΔE_Q (mm/s)	1.431	1.329	0.66

C) Resonance Raman Spectroscopy for Int I: For the computed resonance Raman spectra (Figure S36, Table S19, SI), a vertical excitation energy of 18,259 cm^{-1} and 22,308 cm^{-1} was used for the 6-coordinate and the 5-coordinate conformers of **Int I**, respectively, to fit the calculated excitation energies for the LMCT excitation (N-p to Fe-t_{2g}). For the 6-coordinate Fe(N+O) octahedral isomer the symmetric Fe-O vibrational modes arise at 430 and 442 cm^{-1} (Figure S36). At 504 cm^{-1} there is an intense in-plane deformational mode of the substrate's (aminating agent) O-Fe-N-ring. The asymmetric Fe-O vibrational modes lie at 543 and 555 cm^{-1}. The most intense peak is at 560 cm^{-1}, describing the Fe-N+Fe-O_{subs} stretching mode. Due to the ring-like binding mode of the substrate, the corresponding Fe-N and Fe-O stretches heavily mix (Figure S36). For the 5-coordinate isomer without the iron-keto bond, the symmetric Fe-O stretching modes arise at 439 cm^{-1} and 450 cm^{-1} with increased intensities compared to the 6-corrdinate isomer. This is due to the fact that the corresponding modes mix with the Fe-N vibrational mode. However, the most intense peak is at 629 cm^{-1} for the 5-coordinate isomer, describing a mixture of the Fe-N stretching mode and N-H deformational mode (Figure S36, SI). The experimental resonance Raman spectrum of Int I has several peaks matching with the computed rR spectra following the same trend, with a red shift of the Fe-O_{acac} stretch at 460 cm^{-1} compared to precursor 1 (420 cm^{-1}), (Figure S26 and Table S19A, SI) however, interference of solvent peak around 585 cm^{-1} probably masks the heavily mixed Fe(N+O) stretches for the 6-coordinated conformer. No higher energy Fe-N stretch expected for the

5-coordinate isomer was detected in the experimental spectrum, thereby suggesting once again that this is likely not the preferred conformer for Int I.

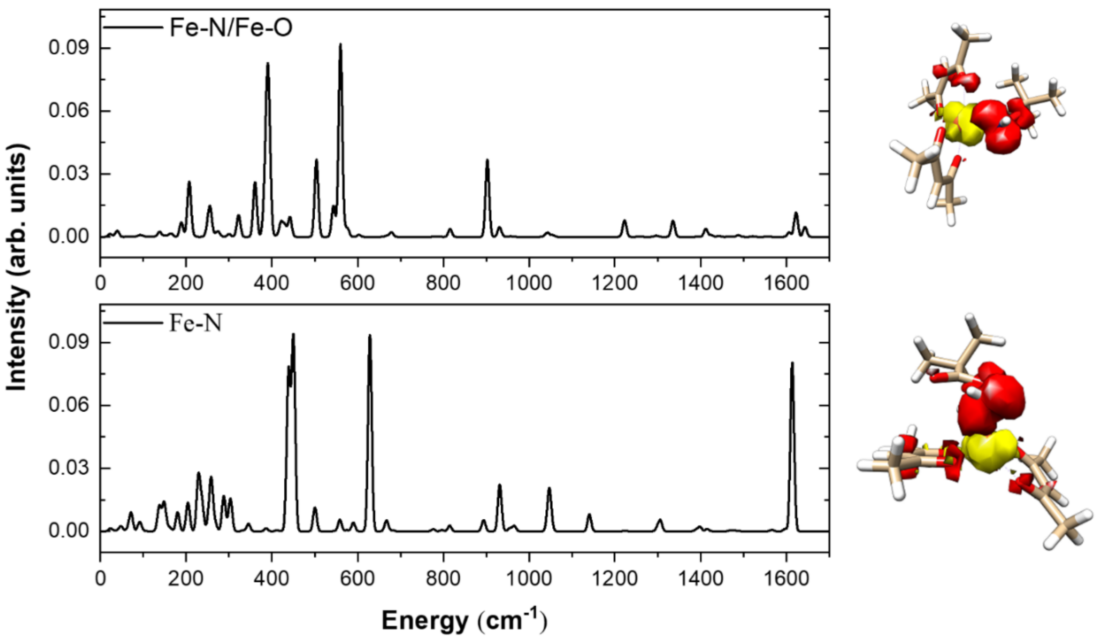

Figure S36. Computed resonance Raman spectra of **Int I**. Top: Fe(N+O) binding mode. Vertical excitation energy is 18259 cm^{-1}. Bottom: Fe-N binding mode. Vertical excitation energy is 22308 cm-1. The difference density plots are shown on the right of the spectrum (donor region in red, acceptor region in yellow).

Table S19A. Selected computed and experimental vibrational frequencies for Int I in the rR spectrum. Only the excitations found in experiment are reported. All energies are in cm^{-1}.

Assignment	**Int I** Fe(N+O)	**Int I** (FeN)	**Int I** Exp.
υs(Fe-O_{acac})	430	439	429
υs(Fe-O_{acac})	442	450	460
υas(Fe-O_{acac})	543	558	532
υs(Fe-N/Fe-O)	560	629	585
υ(N-O)	903	932	921
υ(C-O)	1223	1141	1215
υ(C=CH)	1294	1299	1293
δ(CH_3)	1396	1399	1384
υas(C=O)	1607	1597	1586
υs(C=O)	1623	1614	1614

D) NRVS of Int I: Analogous to rR, the computed Fe-O_{acac} stretch (442 cm^{-1} for 6-coordinate conformer and 447 cm^{-1} for 5-coordinate conformer) in the NRVS (Figure S37, Table S19B, SI) is shifted to higher energy compared to models for precursor **1** (~420 cm^{-1}) and this trend matches well with the experimental NRVS (458 cm^{-1} for **Int I** and 440 cm^{-1} for 1) (Figure S27, Table S9, SI). Taking a closer look at the experimental NRVS for **Int I**, the 393 cm^{-1} stretch assigned to the δ(N-Fe-O) vibration for the 6-coordinate Fe(N+O) conformer matches with the experimental 390 cm^{-1} peak, which is completely absent for the 5-coordinate conformer of **Int I**. Overall the experimental NRVS favors the 6-coordinate Fe(N+O) isomer compared to the 5-coordinate FeN conformer for **Int I** (see Figure S37, Table S19B, SI).

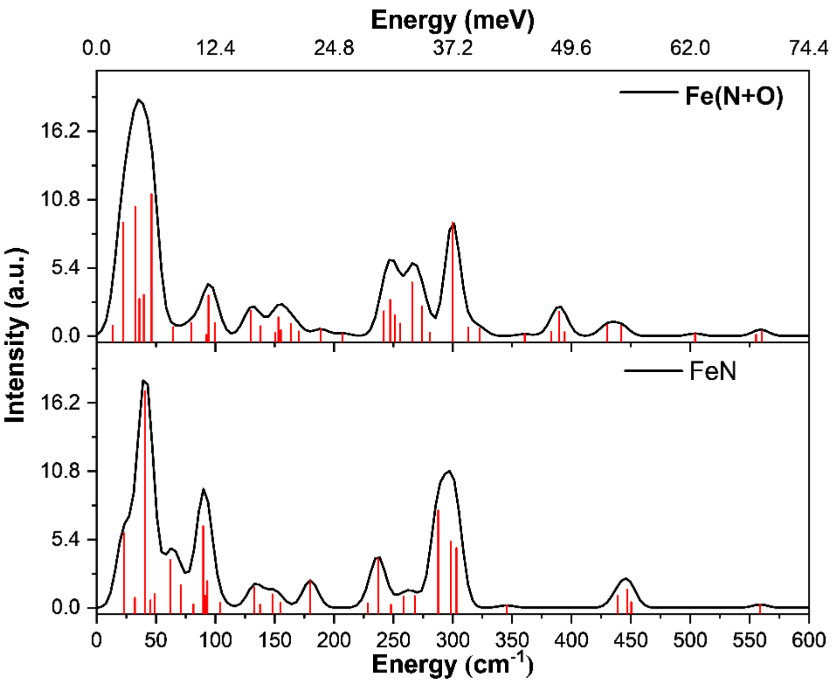

Figure S37. Simulated NRVS spectra for **Int I**: Top: Fe(N+O)-keto binding mode. Bottom: FeN binding mode only.

Table S19B. Selected computed vibrational frequencies for Int I that are present in the NRVS spectrum. All energies are in cm^{-1}

Assignment	**Int I** Fe(N+O)	**Int I** FeN	**Int I** Exp
$\delta(Fe\text{-}O_{acac})$	300	288/298/303	256/280/313
$\upsilon s(Fe\text{-}O_{acac})$	430/442	439/447/450	458
$\upsilon as(Fe\text{-}O_{acac})$	555	559	540

E) Kβ$_{1,3}$ HERFD XAS of Int I: TDDFT-computed pre-edges of **Int I** for both the 6-coordinate Fe(N+O) and the 5-coordinate FeN model systems were pursued to complement the Kβ$_{1,3}$ HERFD XAS experimental results. From the calculated pre-edge

peaks of the 6-coordinate and 5-coordinate models of **Int I** (Figure S38, SI) it becomes very clear that the experimental results clearly favor the 6-coordinate octahedral geometry around the iron center (Figure 5 and Table S21 and Figure S38, SI) with the bidentate mode of the aminating agent via nitrogen and the keto oxygen (Fe(N+O)) for **Int I**. The increase of the energy splitting, ΔE, for **Int I** (ΔE = 1.5 eV), with respect to the precursor complex 1 (ΔE = 1.3 eV) is correctly reproduced by the TDDFT calculations (Table S20). The experimental energy splitting ΔE has a better agreement for the 6-coordinate Fe(N+O) model (ΔE = 1.3 eV) compared to the 5-coordinate FeN model of **Int I**. Moreover, the 5-coordinate model shows a large intensity enhancement especially for the excitations into the σ*-bonds, which does not match with the experimental spectrum; rather the 6-coordinate octahedral conformer has better visual agreement, in terms of intensity distribution and peak splitting (Figure S38, SI). Moreover, the intensities in the experimental pre-edge spectra for the 6-coordinated distorted octahedral geometry of precursor **1** is comparable to that observed for **Int I** (Figure 5a). This further supports the octahedral environment around iron for **Int I**, therefore supporting the Fe(N+O) coordination mode. To assess this more quantitatively, the calculated area, as well as the intensity weighted average energy (IWAE), is compared to the experiment in Table S20. Looking at the calculated IWAEs, both the 6 and 5 coordinate models give good agreement with the experiment, however, considering the pre-edge intensity, the 6-coordinate Fe(N+O) model can clearly be considered as the favored conformer for **Int I**.

Table S20. XAS parameter for both the calculated isomers of the **Int I** and the experimental result. ΔE describes the difference between the two peak maxima in eV.

Entry	**Int I** Fe(N+O)	**Int I** FeN	**Int I** Exp.
IWAE (eV)	7112.75	7112.87	7112.88
Area/Amplitude	18	49	22
ΔE (eV)	1.3	1.9	1.5

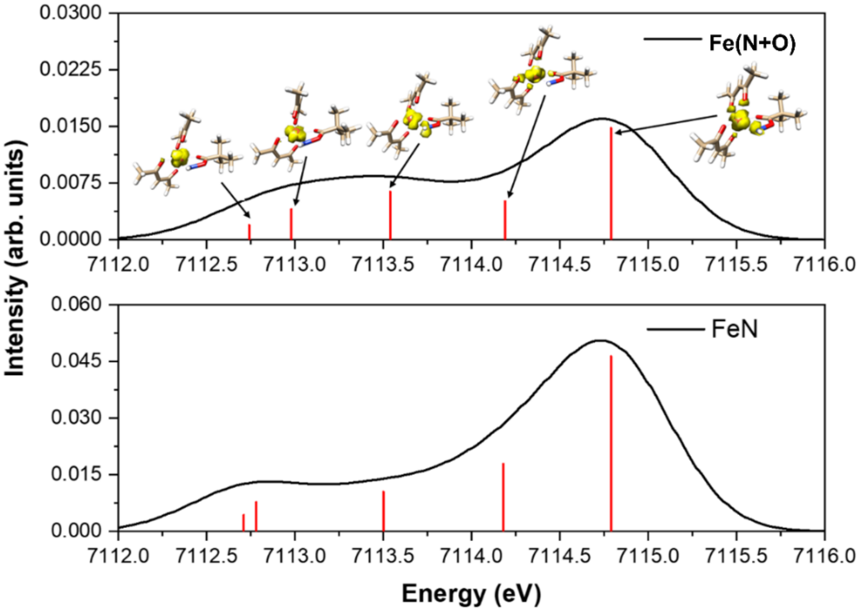

Figure S38. Simulated X-Ray absorption spectra for **Int I**: Top: Fe(N+O) binding mode. Bottom: FeN binding mode. To match the experimental spectra, the simulated spectra were shifted by 23.36 eV and scaled by 0.022. For the Fe(N+O) isomer the calculated difference density plots for the respective excitations were added. The linewidth for the gaussian broadening was 0.8 eV. Only excitations of the β-electrons were allowed.

Fe-iminyl radical species [Fe(acac)$_2$(NH)] (Int II)

Energetics of spin states of Int II

Table S21. Relative free energies for different spin multiplicities for **Int II**. In each case the geometry was optimized in order to take the breathing mode of Fe into account.

Total Spin (S_t)	Spin multiplicity ($2S_t$+1)	Rel. DFT energy (kcal/mol)
0	1	13.900
1	3	8.089
2	5	0.000
3	7	4.101

The quintet is in clear favor over the other spin states. However, the quintet state is described in terms of broken symmetry DFT and the relative energy of the heptet state is not too high in energy. Therefore, the relative energy between the quintet and heptet state was also calculated at the CAS-SCF level, which prefers the quintet by 10.9 kcal/mol. The CAS-SCF calculations were performed on the quintet states geometry, however, the CAS-SCF calculation shows as well, that the quintet state is favored as the spin ground state.

Spectroscopic properties of Int II

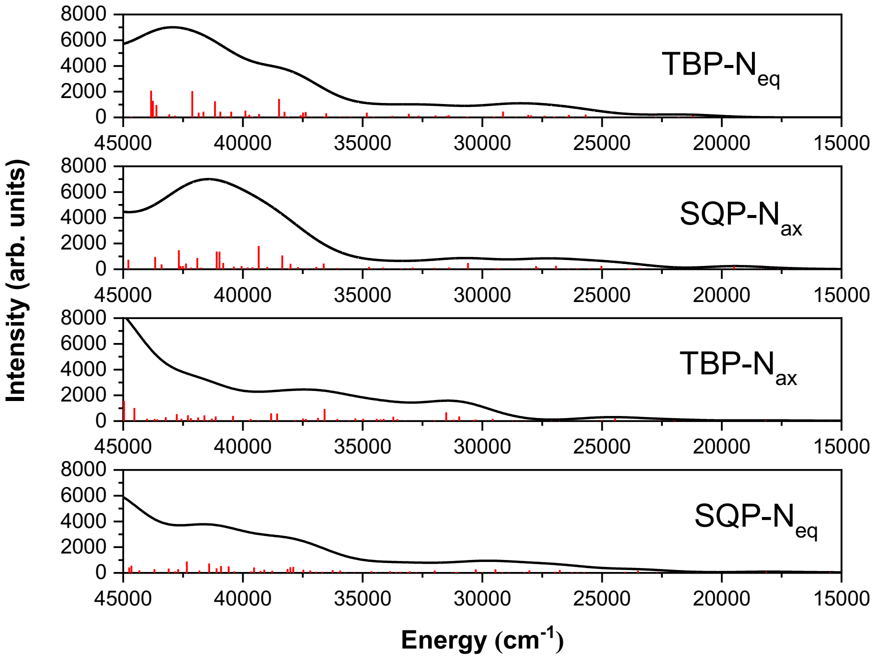

Figure S39. Computed absorption spectrum of **Int II**. From top to bottom: Distorted trigonal-bipyramidal geometry with equatorial Nitrogen. Square-pyramidal geometry with axial Nitrogen. Trigonal-bipyramidal geometry with axial Nitrogen. Square-pyramidal geometry with equatorial Nitrogen. The intensities were are scaled to fit the experimental extinction coefficients.

Table S22. Comparison between theoretical and experimental absorption parameters for **Int II**.

Excitation energy cm^{-1} (**Int II**)					Oscillator strength (**Int II**)				
Exp.	TBP-N_{eq}	SQP-N_{ax}	TBP-N_{ax}	SQP-N_{eq}	Exp.	TBP-N_{eq}	SQP-N_{ax}	TBP-N_{ax}	SQP-N_{eq}
14342	21487	19000	24108	17998	0.019	0.010	0.017	0.020	0.006
27128	28149	26104	31606	29451	0.021	0.091	0.046	0.112	0.112
34606	34938	30833	36799	37784	0.112	0.112	0.065	0.177	0.176
41339	39197	39646	40987	40907	0.136	0.267	0.174	0.130	0.125
	43429	41990	46355	47151		0.408	0.551	0.657	0.524

NB: The presented excitation energies and oscillator strengths are obtained from a gaussian fitting procedure. For good fits, five gaussians were needed for each computed spectrum.

(A) Mössbauer Parameters of Int II: From the calculated Mössbauer spectrum of **Int II**, the quadratic-pyramidal isomer with the axial nitrogen SQP-N_{ax} gives better agreement with the experiment in terms of both the isomer shift and quadrupole splitting (Table S23).

Table S23. Mössbauer parameter for the different geometries of **Int II** and comparison with experiment.

**Int II conformer**	**Mössbauer parameters**	
	δ (mm/s)	ΔE_Q (mm/s)
TBP-N_{eq}	0.41	1.46
SQP-N_{ax}	0.47	0.78
SQP-N_{eq}	0.41	1.66
TBP-N_{ax}	0.41	2.07
Exp.	0.57	0.40

B) Resonance Raman Spectroscopy for Int II: For **Int II**, the computed resonance Raman spectra (Figure S40, Table S24, SI) differ significantly depending on the isomers, mainly due to the different kind of excitation taking place as evident from the difference density plot (Figure S40). For **Int II**, an LMCT transition occurs for all the isomers as also evident from the experimental optical absorption spectrum (Figure 1 and Figure S2). For the trigonal-bipyramidal isomer with the equatorial nitrogen (TBP-N_{eq}), LMCT occurs mainly from the N-p to Fe-t_{2g} with a vertical excitation energy of 21,217 cm^{-1} (Figure S40, Table S24, SI). For the quadratic-pyramidal isomer with axial nitrogen (SQP-N_{ax}) the excitation occurs from the acac-π orbitals to the Fe-N-σ antibonding orbital with a vertical excitation energy of 19,596 cm^{-1} to fit to the LMCT excitation (Figure S40, Table S24, SI). For **Int II** (Figure S26, Table S24, SI), laser excitation at 660 nm gave experimental resonance-enhanced bands at 433 cm^{-1} assigned to the Fe-O symmetric stretch from acac, and the asymmetric Fe-O stretch from acac at 528 cm^{-1}. The intense δ(H-N-Fe) peak at 462 cm^{-1} is slightly sensitive to ^{15}N isotope (from $PivO^{15}NH_3OTf$) and shifts by four wave numbers (Table S25, SI). The computed Resonance Raman for all the conformers of **Int II**, agrees well with the experimentally observed υs(Fe-O_{acac}) and υas(Fe-O_{acac}) stretches as well as the δ(H-N-Fe) stretch with a small calculated ^{15}N isotope shift in agreement with the experimental result (Figure S40, Table S24 and Table S25, SI). However, the distinguishing peak among the model conformers are the Fe-N stretch which occurs at around 600 cm^{-1} for both the TBP-N_{eq} and TBP-N_{ax} isomers, and shifts to higher energy at 623 cm^{-1} for the SQP-N_{eq} model and at 655 cm^{-1} for the SQP-N_{ax} conformer (Figure S40, Table S24, SI). In the experimental rR spectrum of **Int II** (Figure S26, Table S24 and Table S25, SI), the Fe-N stretch is observed at 612 cm^{-1} with small ^{15}N isotope sensitivity, which does not match with the square pyramidal isomers, but matches more with the trigonal bipyramidal geometry.

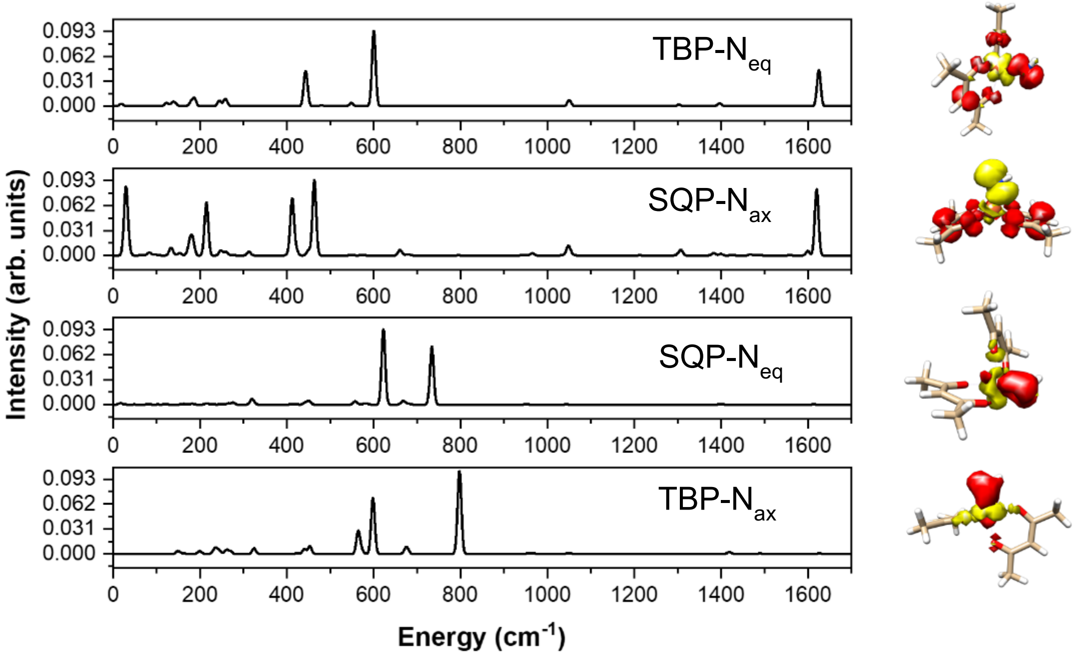

Figure S40. Computed resonance Raman spectra of **Int II**. From Top to Bottom: Trigonal-bipyramidal (TBP-N$_{eq}$) geometry. Vertical excitation energy is 21217 cm^{-1}. Square-pyramidal (SQP-N$_{ax}$) geometry. Vertical excitation energy is 19596 cm^{-1}. Square-pyramidal (SQP-N$_{eq}$) geometry. Vertical excitation energy is 18149 cm^{-1}. Trigonal-bipyramidal (TBP-N$_{ax}$) geometry. Vertical excitation energy is 18175 cm^{-1}. The difference density plots are shown on the right of the spectrum (donor region in red, acceptor region in yellow).

Table S24. Selected computed and experimental vibrational frequencies for **Int II**. Only the excitations found in experiment are reported. All energies are in cm^{-1}.

Assignment of mode	**Int II** TBP-N_{eq}	**Int II** SQP-N_{ax}	**Int II** TBP-N_{ax}	**Int II** SQP-N_{eq}	**Int II** Exp.
υs(Fe-O_{acac})	442/444	448	440/453	443/451	433
δ(N-H)	480	465	798	734	462
υas(Fe-O_{acac})	548/549	549/560	564/574	558	528
υ(Fe-N)	600	655	598	623	612
δ(C-H)	793/795	794/800	794/797/802	796/797	802
δ(CH_3)	1050	1047	1039	1038	1024
υ(C=CH)	1303	1307	1304	1305	1299
δ(CH_3)	1471	1468	1468	1471	1454

Table S25. Computed ^{15}N isomer shifts for selected vibrational frequencies for different conformers of **Int II** and comparison to experimental result.

Mode	**Int II** TBP-N_{eq}	**Int II** SQP-N_{ax}	**Int II** TBP-N_{ax}	**Int II** SQP-N_{eq}	**Int II** Exp.
υs(Fe-O_{acac})	-0.03	-0.03	-0.11	-0.16	0
δ(N-H)	-6.13	-0.95	-1.12	-0.01	-4.0
υas(Fe-O_{acac})	-0.12	0.00	-6.98	-0.61	0
υs(N-H)	-10.04	-15.21	-7.72	-14.59	-4.0

NB: All energies are in cm^{-1}. Isomer shifts both calculated and experimental are very small in total.

C) NRVS of Int II: Analogous to rR, the computed Fe-O_{acac} stretch in the NRVS of **Int II** is shifted to higher energy compared to models for **Int I** and precursor **1** and this trend matches well with the experimental NRVS (461 cm^{-1} for **Int II**, 458 cm^{-1} for **Int I** and 440 cm^{-1} for **1**, Figure S27, Table S9), with a small feature around 583 cm^{-1} for **Int II** in the experimental spectrum corresponding to the Fe-NH stretch (Figure S41, Table S26, SI).

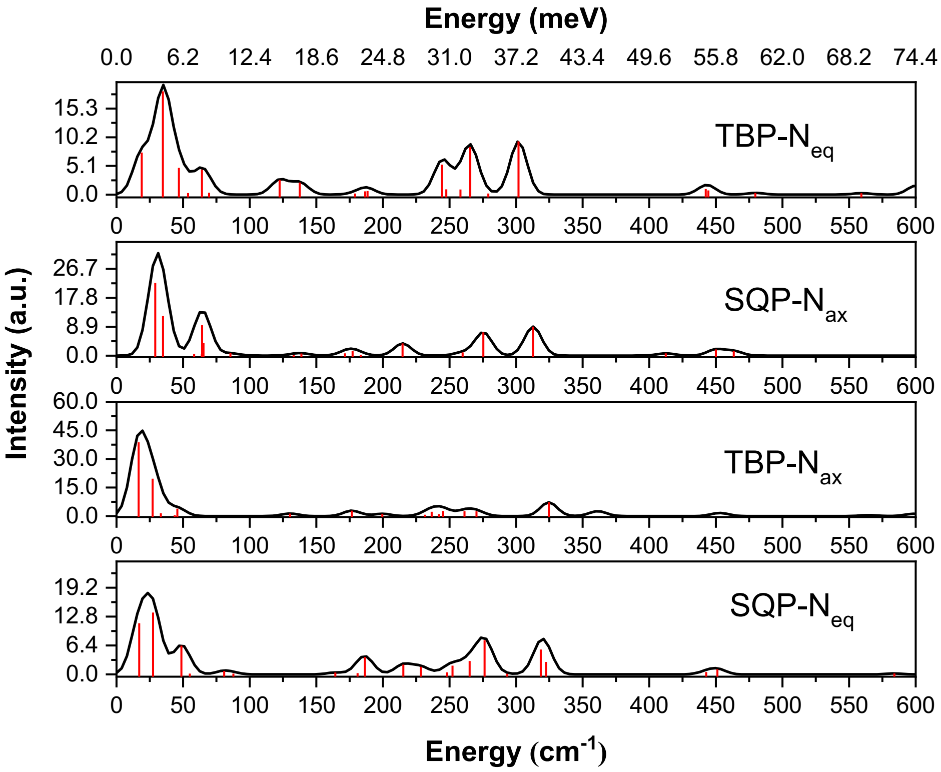

Figure S41. Calculated NRVS spectra for **Int II**. From top to bottom: Distorted trigonal-bipyramidal with equatorial nitrogen (TBP-N_{eq}), Quadratic-pyramidal with axial nitrogen (SQP-N_{ax}), Trigonal-bipyramidal with axial nitrogen (TBP-N_{ax}), Quadratic-pyramidal with equatorial nitrogen (SQP-N_{eq}).

Table S26. Selected computed vibrational frequencies for different conformers of **Int II** that are present in the NRVS spectrum. All energies are in cm^{-1}.

Assignment	**Int II** TBP-N_{eq}	**Int II** SQP-N_{ax}	**Int II** TBP-N_{ax}	**Int II** SQP-N_{eq}	**Int II** Exp.
δ(Fe-O_{acac})	302	311	325	319	313
υs(Fe-O_{acac})	442/444	448	453	443/451	462
υas(Fe-O_{acac})	559	-	564	-	520
υ(Fe-N)	600	655	598	623	583

D) K$\beta_{1,3}$ HERFD XAS of Int II: To obtain insight into the K$\beta_{1,3}$ HERFD XAS experimental results, TDDFT-computed pre-edges of **Int II** for all 4 conformers i.e TBP-N_{eq}, SQP-N_{ax}, SQP-N_{eq} and TBP-N_{eq} were pursued (Figure S42, SI). In the experimental K$\beta_{1,3}$ HERFD XAS, we observed an increase in intensity of the pre-edge peaks compared to **Int I** and precursor **1**, both of which have distorted octahedral geometry (Figure 5b). This is consistent with the 5-coordinate iron center in **Int II** with a decrease in metal symmetry, thereby increasing the dipole contributions due to the 3d-4p mixing. The experimentally observed energy splitting, ΔE for **Int II** (ΔE = 1.2 eV), matches exactly with the calculated energy split for the SQP-N_{ax} isomer where there is a clear separation between the first two and the last three pre-edge excitations (Table S27). The experimentally observed reduction of the energy gap for **Int II** (ΔE = 1.2 eV) (Table S27), with respect to **Int I** (ΔE = 1.5 eV) (Table S20), can also be reproduced for the SQP-N_{ax} model. For the distorted trigonal-bipyramidal isomer with equatorial nitrogen (TBP-N_{eq}) no second peak can be located since there is no clear energetic gap between groups of excitations (Figure S42). However, from the fitting of the pre-edge peaks, the TBP-N_{ax} conformer seem to give better visual agreement, in terms of intensity distribution (Figure S42). To assess this more quantitatively, the calculated area, as well as the intensity weighted average energy (IWAE), is compared to the experiment in Table S27. Looking at the calculated IWAEs, all the models agree very well with experiment. In terms of pre-edge area, the trigonal bipyramid geometries (TBP-N_{ax} and TBP-N_{eq}) seem to give better agreement with

experiment, with the TBP-N_{ax} having better match with the experimental spectral shape. The quadratic bipyramid with an axial nitrogen (SQP-N_{ax}) gives moderate agreement in terms pre-edge area and spectral shape, with the IWAE and ΔE values matching very well with the experiment. The square-pyramid model with the equatorial nitrogen deviates more strongly in terms of both area, spectral shape and ΔE values.

Table S27. XAS parameter for all 4 model conformers of the **Int II** and the experimental value. ΔE describes the difference between the two peak maxima in eV.

Entry	**Int II** TBP-N_{eq}	**Int II** SQP-N_{ax}	**Int II** TBP-N_{ax}	**Int II** SQP-N_{eq}	**Int II** Exp.
IWAE (eV)	7112.74	7112.66	7112.74	7112.97	7112.79
Area/Amplitude	34	38	35	25	32
ΔE (eV)	-	1.2	1.7	1.6	1.2

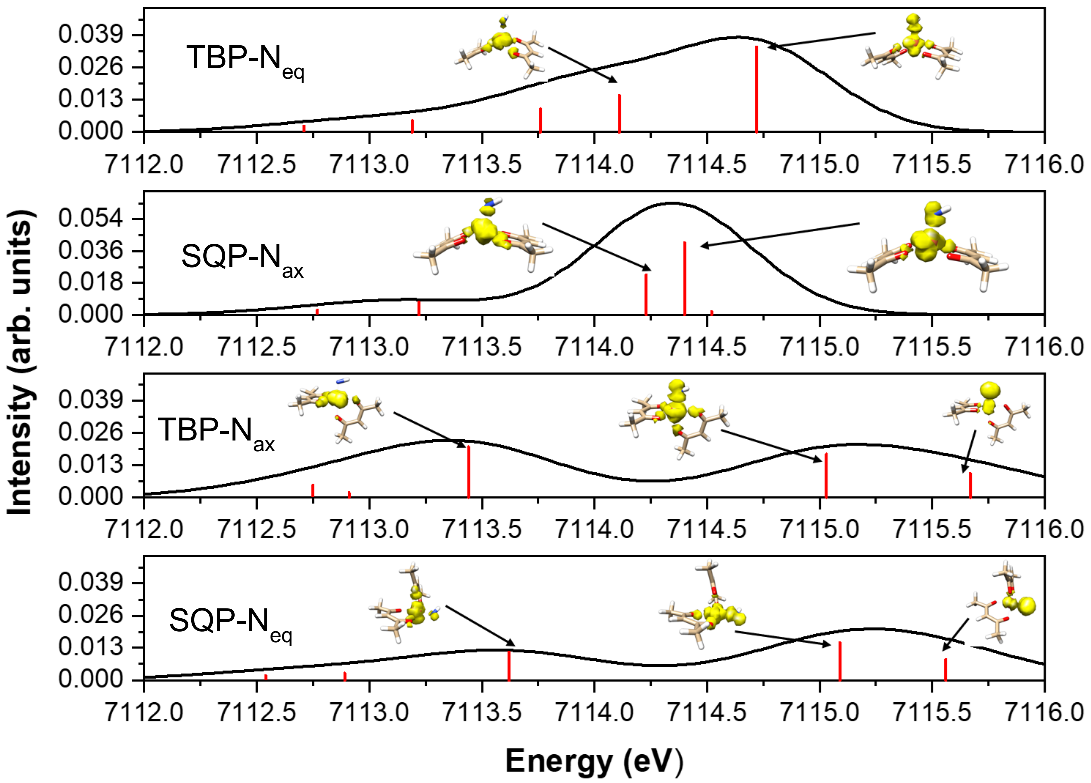

Figure S42. TDDFT calculated pre-edge region of HERFD-X-Ray absorption spectra for **Int II**: From top to bottom: Distorted trigonal-bipyramidal geometry with equatorial nitrogen (TBP-N_{eq}). Square-pyramidal geometry with axial nitrogen (SQP-N_{ax}). Trigonal-bipyramidal geometry with axial nitrogen (TBP-N_{ax}). Square-pyramidal geometry with equatorial nitrogen (SQP-N_{eq}). To match the experimental spectra, the calculated spectra were shifted by 23.36 eV and scaled by 0.022. For all isomers the calculated difference density plots for the dominant respective excitations were added. The linewidth for the gaussian broadening was 0.8 eV. Only excitations of the β-electrons were allowed.

NBO analyses: The NBO analysis for the energetically favorable conformer of **Int II**, the TBP-N_{eq}, shows that a significant portion of the spin density resides at the nitrogen atom, while the spin population at the nitrogen atom for the SQP-N_{ax} model is smaller but still negative (entry 2 and 3, Table S28). Taken together, the electronic structure of **Int II** (TBP-N_{eq} and SQP-N_{ax}) can be described in terms of broken-symmetry DFT. The nitrogen radical (S = 1/2) couples antiferromagnetically with the high spin Fe(III) center (S = 5/2) to form an overall spin state of S_t = 2 for **Int II**. The coupling constant J is defined via [49-50]

$$J = (\mathrm{E[HS]} - \mathrm{E[BS]})/(< S^2 >_{\mathrm{HS}} - < S^2 >_{\mathrm{BS}})$$

and comes to -524.41 cm^{-1} for the TBP-N_{eq} isomer and to -893.41 cm^{-1} for the SQP-N_{ax} isomer at the BS-DFT level. Comparison of the NBO atomic charges for Int I and Int II is shown in Table S28.

Table S28. NBO analysis for model conformers of **Int I** and **Int II** with respect to Fe and N at the B3LYP-D3/def2-TZVP level.

Entry	Intermediate model type	Natural charge (e)		Natural spin density	
		Fe	N	Fe	N
1.	**Int I** Fe(N+O)	1.76	-0.62	3.94	0.35
2.	**Int II** TBP-N_{eq}	1.71	-0.74	3.90	-0.52
3.	**Int II** SQP-N_{ax}	1.61	-0.68	3.60	-0.13
4.	**Int II** TBP-N_{ax}	1.48	-0.62	3.08	0.53
5.	**Int II** SQP-N_{eq}	1.50	-0.63	3.16	0.47

From Table S28, it can be seen, that while the charge and spin density of the iron center is unaffected in the TBP-N_{eq} conformer of **Int II** compared to **Int I**, the charge on the nitrogen significantly decreases for both the TBP-N_{eq} and SQP-N_{ax} conformers of **Int II**. The spin density of the TBP-N_{eq} conformer increases in value, but has a negative sign, which reflects the antiferromagnetic coupling between iron and nitrogen in **Int II**. For the SQP-N_{ax} isomer the spin density decreases and thus becomes closer to zero. The bond between the high spin iron and the nitrogen radical (iron-iminyl bond) can be understood in terms of one σ/σ* and one π/π* orbital pair. The σ- and π-bonding orbitals are occupied for both the TBP-N_{eq} and SQP-N_{ax} isomers. For the TBP-N_{eq} isomer the σ*-orbital carries one electron while the π*-orbital is empty. For the SQP-N_{ax} isomer the σ*-orbital is empty while the π*-orbital is partially occupied.

Löwdin Charge and spin density: CAS-SCF calculations with 12 electrons in 11 orbitals was performed on the electronic ground state. The starting orbitals are, however, the QROs obtained from the DFT calculation. Interestingly, different from DFT, CAS-SCF

predicts very similar electronic structures and therefore, atomic charges and spin populations for all isomers of **Int II** with sufficient negative spin population on the nitrogen (Table S29)

Table S29. Löwdin charge and spin analysis for **Int II** with respect to Fe and N based on CAS(12,11) calculations.

Entry	**Int II** model	Löwdin charge (e)		Löwdin spin density	
		Fe	N	Fe	N
1.	TBP-N_{eq}	0.75	-0.38	4.31	-0.45
2.	SQP-N_{ax}	0.71	-0.34	4.24	-0.39
3.	TBP-N_{ax}	0.68	-0.33	4.19	-0.34
4.	SQP-N_{eq}	0.68	-0.33	4.22	-0.36

Table S30. DFT-Calculated Bond lengths of the different conformers of **1**.

**Bond**	**Distance (Å)**	
	**1-*cis***	**1-*trans***
Fe-OH_2(1)	2.429	2.283
Fe-OH_2(2)	2.290	2.282
Fe-O_{acac} (mean)	2.047	2.047

Table S31. DFT-Calculated Bond lengths of the different conformers of **Int I**.

**Bond**	**Distance (Å)**	
	**Int I** Fe(N+O)	**Int I** FeN
Fe-N	2.003	1.964
Fe-O_{keto}	2.180	3.632
Fe-O_{acac} (mean)	2.028	2.000

Table S32. DFT-Calculated Bond lengths of the different conformers of **Int II**.

**Bond**	**Distance (Å)**			
	**Int II** (TBP-N_{eq})	**Int II** (SQP-N_{ax})	**Int II** (TBP-N_{ax})	**Int II** (SQP-N_{eq})
Fe-N	1.856	1.763	1.759	1.742
Fe-O_{acac} (mean)	2.006	1.997	1.972	1.980

Table S33. Comparison of the experimental spectroscopic parameters with computational calculations for the different reaction components (**1**, **Int I** and **Int II**) considered in this study.

Entry	Calculated spin state and energy	CW EPR	Mössbauer (mm/s)	HERFD-XAS	rR (cm^{-1})	NRVS (cm^{-1})
**1** Exp.	-	EPR silent	δ = 1.26; ΔE_Q = 2.6	IWAE (eV) = 7112.95; ΔE (eV) = 1.3 Area = 25	265; 450; 1284; 1625	256; 300; 440
**1**-*cis*	S_t = 2; 0.95 kcal/mol	-	δ = 1.05; ΔE_Q = 2.6	IWAE (eV) = 7112.84; ΔE (eV) = 1.1 Area = 42	256; 420; 1289; 1618	256; 279; 420
**1**-*trans*	S_t = 2; 0 kcal/mol	-	δ = 1.03; ΔE_Q = 2.5	IWAE (eV) = 7112.74; ΔE (eV) = 1.4 Area = 19	262; 417; 1290; 1610	262; 268; 417
**Int I** Exp.	-	g_{eff} = 9.3, 8.6, 5.4, 4.3 and g_{eff} = 4-2 (broad tail)	δ = 0.55; ΔE_Q = 0.66	IWAE (eV) = 7112.88; ΔE (eV) = 1.5 Area = 22	434; 460; 532; 937; 1188; 1293; 1384;1586; 1606	256; 280; 313; 390; 458; 550
**Int I** 6-coord	S_t = 5/2; 0 kcal/mol	-	δ = 0.61; ΔE_Q = 1.43	IWAE (eV) = 7112.75; ΔE (eV) = 1.3	430; 442; 543; 560; 903; 1223; 1294;	266; 274; 300; 394;

Fe(N+O)				Area = 18	1396; 1607; 1623	430; 442; 555; 560
**Int I** 5-coord. FeN	S_t = 5/2; 5.6 kcal/mol		δ = 0.52; ΔE_Q = 1.33	IWAE (eV) = 7112.87; ΔE (eV) = 1.9 Area = 49	439; 450; 558; 629; 932; 1141; 1299; 1399; 1597; 1614	237; 288; 298; 303; 439; 447; 450; 559; 628
**Int II** Exp.	-	EPR silent	δ = 0.57; ΔE_Q = 0.40	IWAE (eV) = 7112.79; ΔE (eV) = 1.2 Area = 32	433; 462; 528; 612; 802; 1024; 1299; 1454	241; 280; 313; 462; 520; 583
**Int II** TBP-N_{eq}	S_t = 2; 0 kcal/mol	-	δ = 0.41; ΔE_Q = 1.46	IWAE (eV) = 7112.74; ΔE (eV) = - Area = 34	442; 444; 480; 548; 559; 600; 794; 795; 1050; 1303; 1471	245; 266; 302; 442; 444; 559; 600
**Int II** SQP-N_{ax}	S_t = 2; 2.3 kcal/mol	-	δ = 0.47; ΔE_Q = 0.78	IWAE (eV) = 7112.66; ΔE (eV) = 1.2 Area = 38	448; 465; 549; 560; 655; 795; 800; 1047; 1306; 1468	275; 311; 448; 655
**Int II** SQP-N_{eq}	S_t = 2; 4.0 kcal/mol	-	δ = 0.41; ΔE_Q = 2.07	IWAE (eV) = 7112.97; ΔE (eV) = -1.6 Area = 25	443; 451; 558; 623; 734; 797; 1038; 1306; 1471	276; 319; 443; 451; 623

**Int II** TBP-N_{ax}	S_t = 2; 4.2 kcal/mol	-	δ = 0.41; ΔE_Q = 1.66	IWAE (eV) = 7112.74; ΔE (eV) = 1.7 Area = 35	440; 453; 564; 574; 798; 1041; 1304; 1470	325; 453; 564; 598

Catalyst activation:

Step 1 Oxidation of the precursor 1:

$$\mathrm{Fe(acac)_2(H_2O)_2} + t\mathrm{BuCOONH_3^+OTf^-} \rightarrow \mathrm{[Fe(acac)_2(H_2O)_2]^+} + t\mathrm{BuCOO\cdot} + \mathrm{NH_3} + \mathrm{OTf^-}$$

Step 2 $t\mathrm{BuCOONH_3^+}$ coordination:

$$\mathrm{[Fe(acac)_2(H_2O)_2]^+} + t\mathrm{BuCOONH_3^+OTf^-} \rightarrow \mathrm{[Fe(acac)_2NHCOO}t\mathrm{Bu]} + \mathrm{2H_2O} + \mathrm{2H^+} + \mathrm{OTf^-}$$

Step 1 + Step 2

$$\mathbf{[Fe(acac)_2(H_2O)_2]} + \mathbf{2}\boldsymbol{t}\mathbf{BuCOONH_3^+OTf^-} \rightarrow \mathbf{[Fe(acac)_2NHCOO}\boldsymbol{t}\mathbf{Bu]} + \boldsymbol{t}\mathbf{BuCOO\cdot} + \mathbf{NH_3} + \mathbf{2H_2O} + \mathbf{2H^+} + \mathbf{2OTf^-}$$

In Step 1, the electron transferred by the precursor molecule **1**, induces the homolytic cleavage of the aminating reagent's (H_2N-OPiv.HOTf) N-O bond along with the formation of ammonia and *t*BuCOO$^\bullet$ radical (Step 1). The free reaction energy for the first reaction step is +17.39 kcal/mol. The reason for the high calculated energy is the cleavage of the *t*BuCOONH_3^+OTf^- ion pair, while no ion pairs are formed at the product side. After Step 2, the protons released need to be distributed over the possible available species (H_2O, OTf^-, **Int I**, NH_3). Since no ammonia gas was detected during the reaction, it is highly probable that ammonium triflate is formed during the activation. This is consistent with the fact that ammonia is the strongest base in the system. Ion-pairs between the remaining triflate and associated cations are also likely to be formed in solution. The final reaction equation leading to the formation of **Int I** can, thus, be summarized as:

$$\mathbf{[Fe(acac)_2(H_2O)_2]} + \mathbf{2}\boldsymbol{t}\mathbf{BuCOONH_3^+OTf^-} \rightarrow \mathbf{[Fe(acac)_2NHCOO}\boldsymbol{t}\mathbf{Bu]} + \boldsymbol{t}\mathbf{BuCOO\cdot} + \mathbf{NH_4^+OTf^-} + \mathbf{HOTf} + \mathbf{2H_2O}$$

The corresponding free reaction energy is -7.0 kcal/mol (Figure 9). Subsequent reactions like the protolysis equilibrium of the formed acids were not considered.

Simulations of reaction kinetics

In order to connect the calculated reaction profile, in Figure 9 of the main manuscript, to experimental findings about intermediate accumulation, the concentration profiles for different intermediates were simulated based on the free activation energies obtained from DFT calculations. From the given free activation energies the rate constants are calculated from Eyring equation.[51] The resulting system of coupled differential equations was then solved numerically. The initial conditions are: c(1) = 0.032 mol/L, c(2) = 0.803 mol/L, c(3) = 0.321 mol/L, c(4) = 17.802 mol/L, T = 293 K, where c is the concentration and T the reaction temperature. The initial concentrations of the remaining species are zero. Two different simulations were carried out: First, the kinetic profile in the absence of styrene was simulated. Experiments under the same conditions show the accumulation of **Int II** within 110 minutes. In the presence of styrene, no intermediate besides **Int I** is unambiguously found in experiment. We discuss first the results in the absence of styrene. Figure S43 shows the experimental concentration profiles for **Int I** and **Int II** together with the simulated profiles based on the transition state energies given in Figure 9. In addition, the transition state energies were fitted to reproduce the experimental profiles best.

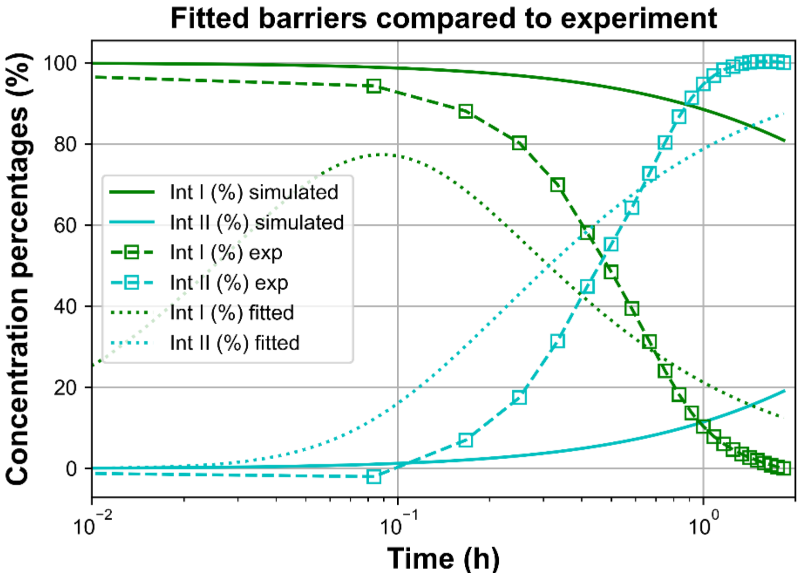

Figure S43. Time-dependence of experimental, simulated and fitted concentration percentages of **Int I** and **Int II**. Squares indicate experimental data points. The fitting process was carried out by integrating the system of differential equations for each 100 different transition state energies associated with the formations of **Int I**, **I-R** and **Int II** in the range of 0.0 kcal/mol to 25.0 kcal/mol leading to 100^3 simulations in total. The barrier for the formation of Int I was not calculated at the DFT-level but is known to be small from experiment.

The RMSE between the best fit and the experimental data is 24.7%. Note, that the error bars of the corresponding transition state energies are large. Within a RMSE of 0.5%, the difference between the maximum and the minimum transition state energies are 17.2 kcal/mol, 12.1 kcal/mol and 16.7 kcal/mol, respectively. This means, that many different tuples of barriers can be chosen to achieve fits of almost the same quality.

In Figures S44 and S45 the simulated concentration profiles in the absence of styrene are shown.

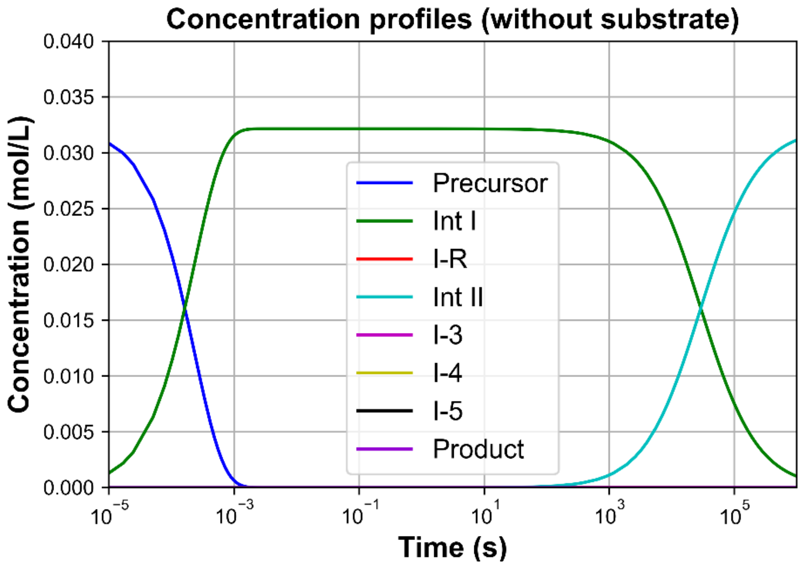

Figure S44. Concentration profiles in the absence of styrene. Linear concentration scale.

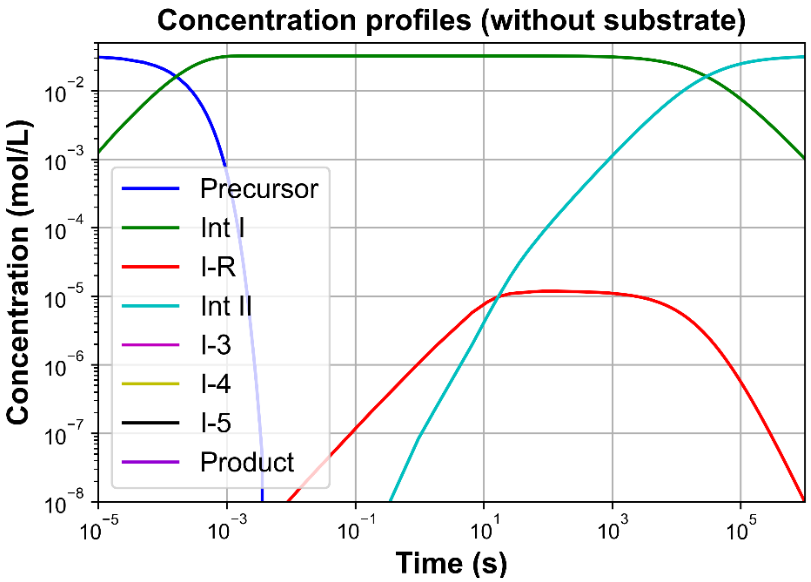

Figure S45. Concentration profiles in the absence of styrene. Logarithmic concentration scale.

From Figure S45 it can be seen that the maximal concentration of I-R is too low to allow detection which agrees with the experiment.

In addition, simulations in the presence of styrene were carried out. The reaction profile in Figure 9 describes the regeneration of the catalyst $Fe(acac)_2$ during the decomposition of

I-3. Simulations were carried out with and without the reactivation of Fe(acac)$_2$ leading to **Int I**. We discuss the simulations without reactivation first. In this case the maximum product concentration is capped by the maximum precursor concentration. The results are shown in Figures S46 and S47.

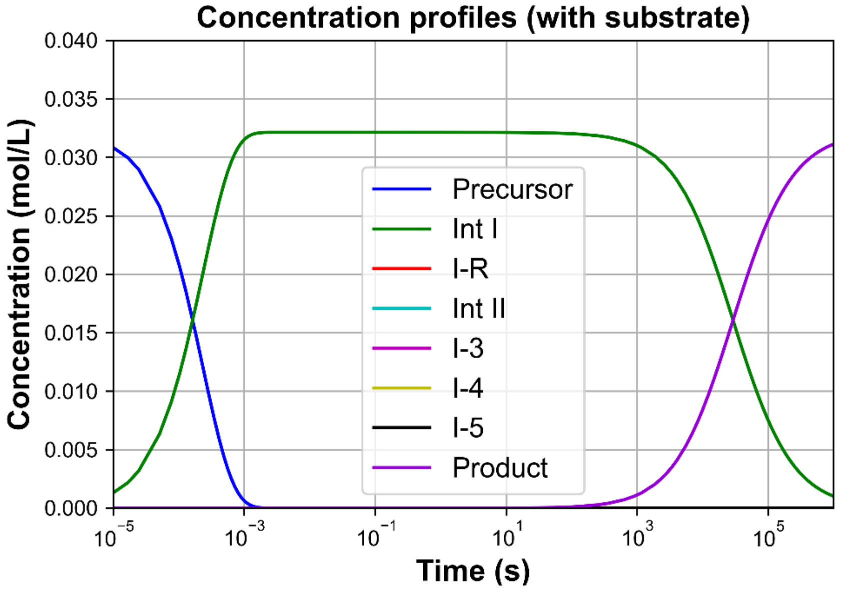

Figure S46. Concentration profiles in the presence of styrene but without catalyst regeneration. Linear concentration scale.

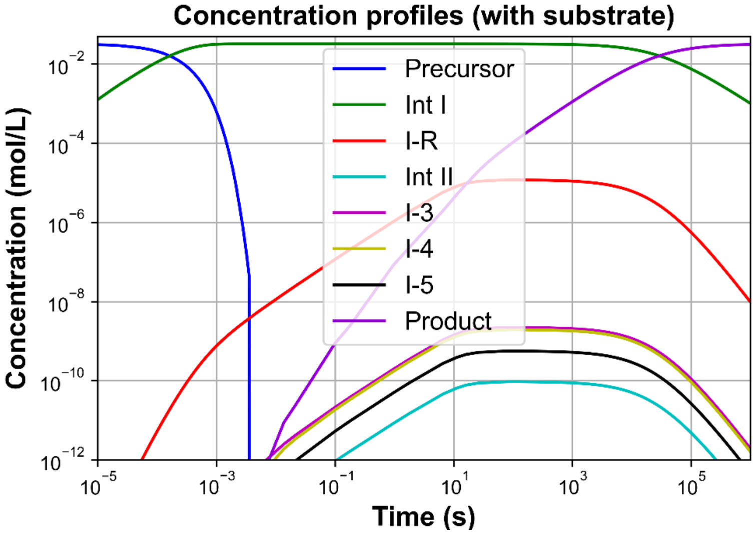

Figure S47. Concentration profiles in the presence of styrene but without catalyst regeneration. Logarithmic concentration scale.

As can be seen, none of the intermediates besides **Int I** accumulates significantly during the reaction course. This is in agreement with the experiment.

Finally, simulations in the presence of styrene and with enabled catalyst regeneration were carried out. The maximum concentration of the product is determined by the maximum substrate concentration. Here, styrene is the reaction limiting compound. The results can be found Figures S48 and S49.

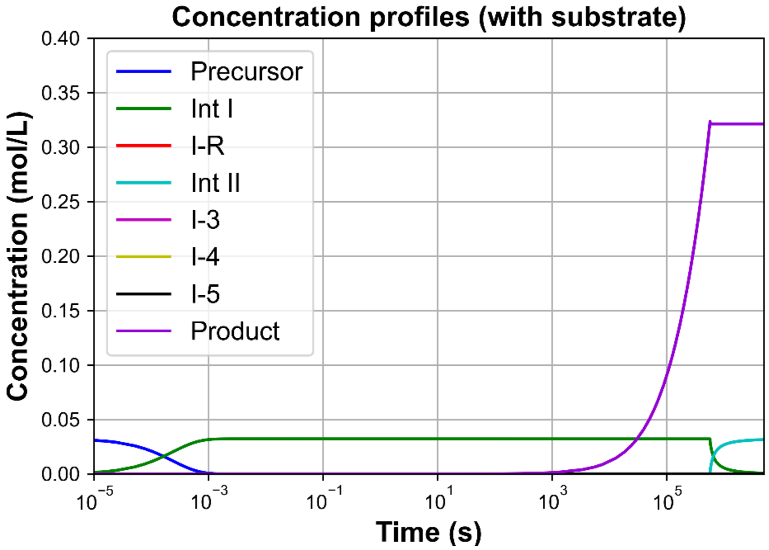

Figure S48. Concentration profiles in the presence of styrene but without catalyst regeneration. Linear concentration scale.

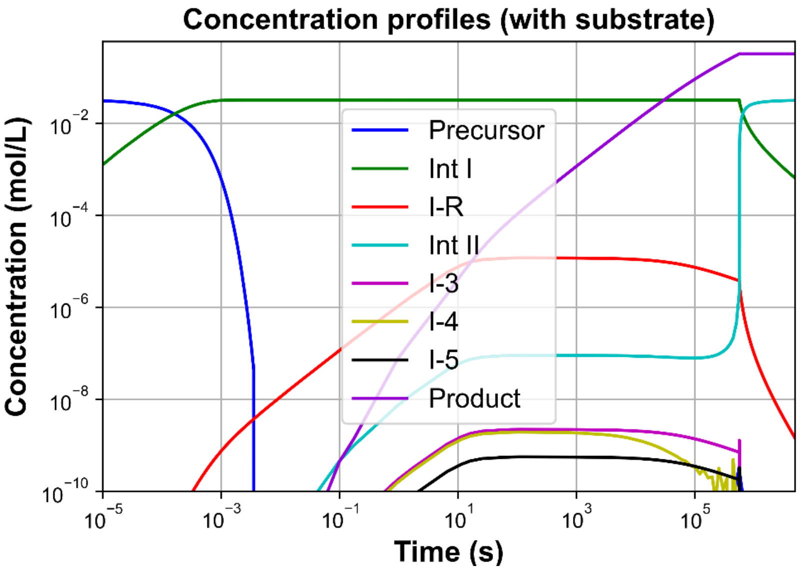

Figure S49. Concentration profiles in the presence of styrene, but without catalyst regeneration. Logarithmic concentration scale.

The steady-state concentration of **Int I** corresponds to the initial concentration of the precursor, since the formation of **Int I** is much faster than its consumption. Again, no other intermediate accumulates during the course of the reaction, which is in agreement with the experimental findings. The maximum concentration of the product corresponds to the initial concentration of styrene. After styrene is completely consumed, **Int II** starts to accumulate again, since the aminating reagent was used in excess, but no consecutive reaction for **Int II** is possible.

Step-wise reaction mechanism

The decomposition of **I-R** to form **Int II**, as shown in Figure 9, consists of three concerted bond cleavages: (i) the Fe-O bond involving the keto group of the PivONH ligand, (ii) the N-O bond and (iii) the C-C bond. In the step-wise mechanism, the Fe-O bond involving the keto group of the PivONH ligand is cleaved first. The formation of Int II is then reached by the concerted cleavage of the N-O bond and the C-C bond. The optimized transition state geometry TS-2s of the step-wise mechanism is shown in Figure S50.

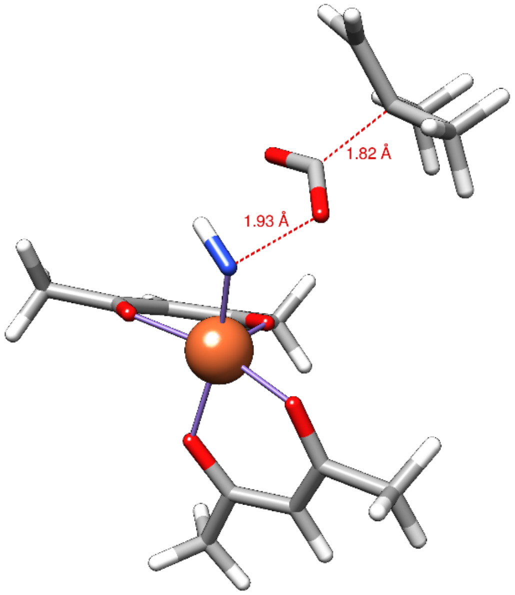

Figure S50. Transition state TS-2s leading to the formation of **Int II** in a step-wise mechanism, where the Fe-O_{PivONH} bond is already cleaved.

The Gibbs free energy of TS-2s relative to the precursor is 24.9 kcal/mol. We carried out kinetic simulations using this barrier and found reaction times that are much longer than those observed experimentally. Hence, this mechanism is not consistent with the experimental kinetic studies. Stimulated by these results, we searched for another plausible mechanism for the formation of **Int II**. This led to the identification of the concerted mechanism discussed in the main manuscript. The structure of the corresponding TS is shown in Figure S51. Repeating the kinetic simulations with the transition state energy of TS-2 yields reasonable concentration profiles within the common error ranges (see Figure 10 and the previous section of the SI), agreeing with the experimental results and validating the calculated reaction pathway (Figure 9).

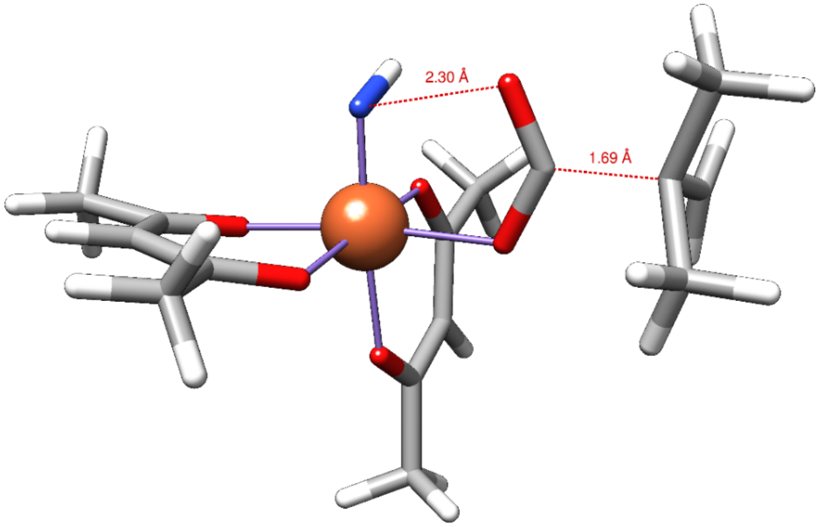

Figure S51. Transition state TS-2 leading to the formation of **Int II** in a concerted mechanism. Its relative Gibbs free energy is 15.2 kcal/mol, which is 9.7 kcal/mol smaller than that of TS-2s. This illustrates the importance of the additional Fe-O bond for the system's stability.

Plausible alternate reaction pathways:

The reaction pathway considered in Figure 9 of the main manuscript is not only validated by the simulated kinetic analyses which agrees well with the experimental kinetic profile, but it is also the lowest energy pathway found from computaional analyes. The *t*BuCOO$^{\bullet}$ radical, which is generated during the activation of Fe(II) catalyst 1 by the hydroxyl amine derived N-O reagent (PivONH$_3$OTf) to form **Int I** (Figure 9), abstracts a hydrogen atom from **Int I** leading to the formation of a transient radical intermediate **I-R**, thereby lowering the barrier for the homolytic cleavage of the N-O bond of Int I. We also computed the direct decomposition of **Int I**, leading to **Int II**, which had a very high energy barrier compared to the one postulated in Figure 9 (see Figure S52) and so was not considered.

Moreover, considering the protonated form of the reagent (PivONH$_3$OTf), we also considered a doubly protonated version of **Int I**, i.e **[Int I-H]**$^+$ [Fe(acac)$_2$NH$_2$COO*t*Bu]$^+$ as the first putative reaction intermediate. The key computed spectroscopic parameters such as Mössbauer, XAS and vibrational frequencies, do not vary much between **Int I** and **[Int I-H]**$^+$ as evident from the calculated spectroscopic parameters given below. So it could be

very likely that **Int I** is in equilibrium with its protonated counterpart [**Int I-H**]$^+$ under the acidic reaction condition. We thus considered calculating the reaction pathway with the protonated form of **Int I** i.e [**Int I-H**]$^+$. However for the protonated species [Int I-H]$^+$, a much higher energy barrier was calculated for both T.S-1 and T.S-2 (Table S34), implying further that the deprotonated species (**Int I**) is significantly more active in the reaction pathway described in Figure 9.

Table S34. Comparative view of energy barriers for the different calculatd pathways.

Entry	Free activation energy	
	TS-1	TS-2
Step-wise **Int I** →**Int II**	21.1	18.5
Direct **Int I** →**Int II**	43.9	
Step-wise [**Int I-H**]$^+$ → [**Int II-H**]$^+$	28.4	42.2
Direct [**Int I-H**]$^+$ → [**Int II-H**]$^+$	37.4	

Note: The step-wise mechanism describe the hydrogen abstraction from **Int I** by the *t*Bu-radical followed by the homolytic cleavage of the N-O bond to form **Int II**, CO_2 and isobutene (see Figure 9). The direct pathway describes the homolytic cleavage of the N-O bond of **Int I** to form **Int II**, CO_2 and the *t*Bu radical. All energies are in kcal/mol.

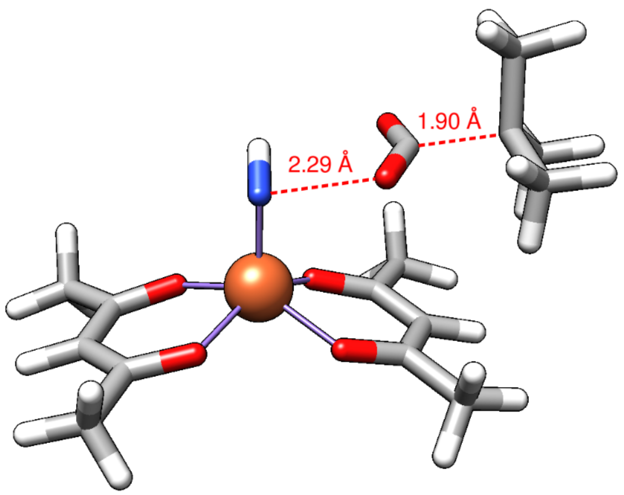

Figure S52: Higher energy transition state for the direct decomposition of **Int I** to **Int II**.

Comparison of calculated spectroscopic parameters between Int I and [Int I-H]$^+$

Similar to **Int I**, the Fe(N+O) and the FeN binding mode were calculated for [**Int I-H**]$^+$. The Fe(N+O) is energetically favored by 7.3 kcal/mol indicating the importance of the additional Fe-O bond in [**Int I-H**]$^+$, and consistent with the results obtained for **Int I**. Here, we present only the spectroscopic properties of the Fe(N+O) isomer for **Int I** and [**Int I-H**]$^+$.

Table S35. Calculated Mössbauer parameters for **Int I** and [**Int I-H**]$^+$ for comparison.

Mössbauer parameter	[**Int I-H**]$^+$ Fe(N+O)	**Int I** Fe(N+O)	**Int I** Exp.
δ (mm/s)	0.564	0.609	0.55
ΔE_Q (mm/s)	1.239	1.431	0.66

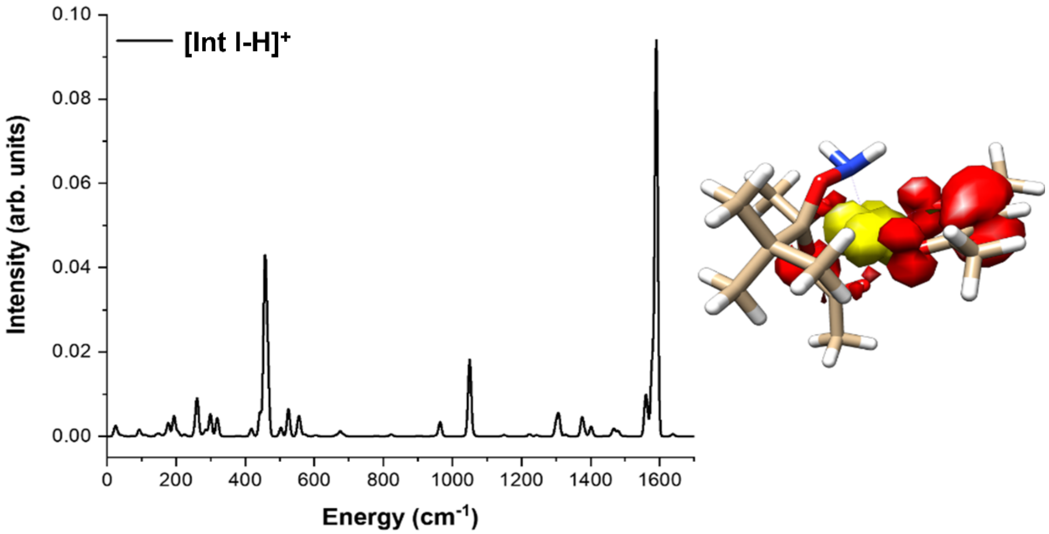

Figure S53: Computed resonance Raman spectrum of protonated **[Int I-H]$^+$**. Fe(N+O) binding mode. Vertical excitation energy is 17692 cm^{-1}. The difference density plot is shown on the right of the spectrum (donor region in red, acceptor region in yellow).

Table S36. Selected computed and experimental vibrational frequencies for **Int I** and [**Int I-H**]$^+$ for comparison. Only the excitations found in experiment are reported. All energies are in cm^{-1}.

Assignment	**Int I** Fe(N+O)	[**Int I-H**]$^+$ Fe(N+O)	**Int I** Exp.
$\upsilon s(Fe\text{-}O_{acac})$	430	443	429
$\upsilon s(Fe\text{-}O_{acac})$	442	456	460
$\upsilon as(Fe\text{-}O_{acac})$	543	554	532
υs(Fe-N/Fe-O)	560	443	585
υ(N-O)	903	944	921
υ(C-O)	1223		1215
υ(C=CH)	1294	1300	1293
$\delta(CH_3)$	1396	1400	1384
υas(C=O)	1607		1586
υs(C=O)	1623	1590	1614

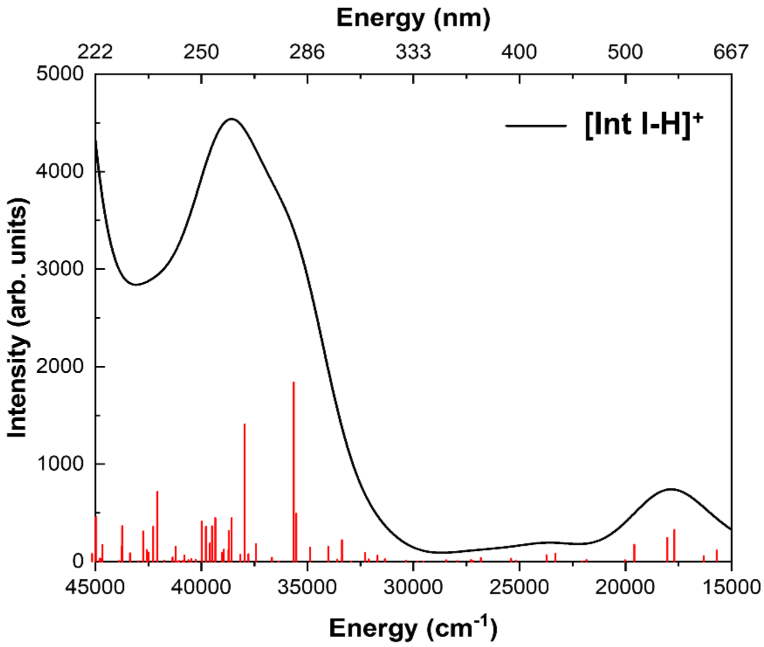

Figure S54. Computed absorption spectrum of protonated **[Int I-H]$^+$**. The intensities are scaled to fit the experimental extinction coefficients.

Table S37. Comparison between theoretical and experimental absorption parameters for **Int I** and [**Int I-H**]$^+$. The presented excitation energies and oscillator strengths are obtained from a gaussian fitting procedure.

Excitation energy cm^{-1}			Oscillator strength		
Exp.	**Int I**	[**Int I-H**]$^+$	Exp	**Int I**	[**Int I-H**]$^+$
20656	18051, 21918	17650	0.040	0.043, 0.065	0.066
28018	25980	25000	0.009	0.043	0.021
33819	38501	35534	0.066	0.163	0.194
41070	41748	39394	0.180	0.562	0.331

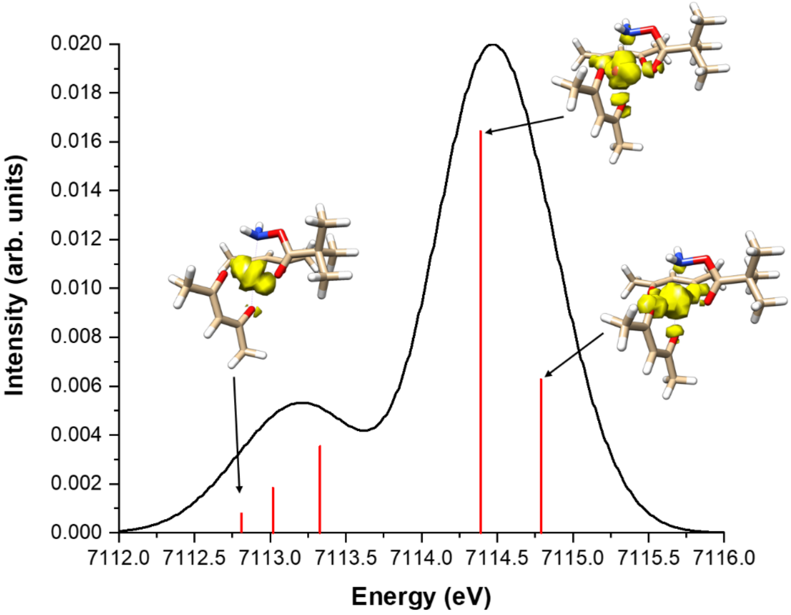

Figure S55. Computed X-Ray absorption spectra for **[Int I-H]⁺** Fe(N+O) binding mode. To match the experimental spectra, the simulated spectra were shifted by 23.36 eV and scaled by 0.022. For the Fe(N+O)-isomer the calculated difference density plots of selected excitations were added. The linewidth for the gaussian broadening was 0.8 eV. Only excitations of the *β*-electrons were allowed.

Table S38. XAS parameter for both the calculated Fe(N+O) isomers of **Int I** and **[Int I-H]⁺** and the experimental result for comparison. ΔE describes the difference between the two peak maxima in eV

Entry	**Int I** Fe(N+O)	**[Int I-H]**⁺ Fe(N+O)	**Int I** Exp.
IWAE (eV)	7112.75	7112.87	7112.88
Area/Amplitude	18	16	22
ΔE (eV)	1.3	1.3	1.5

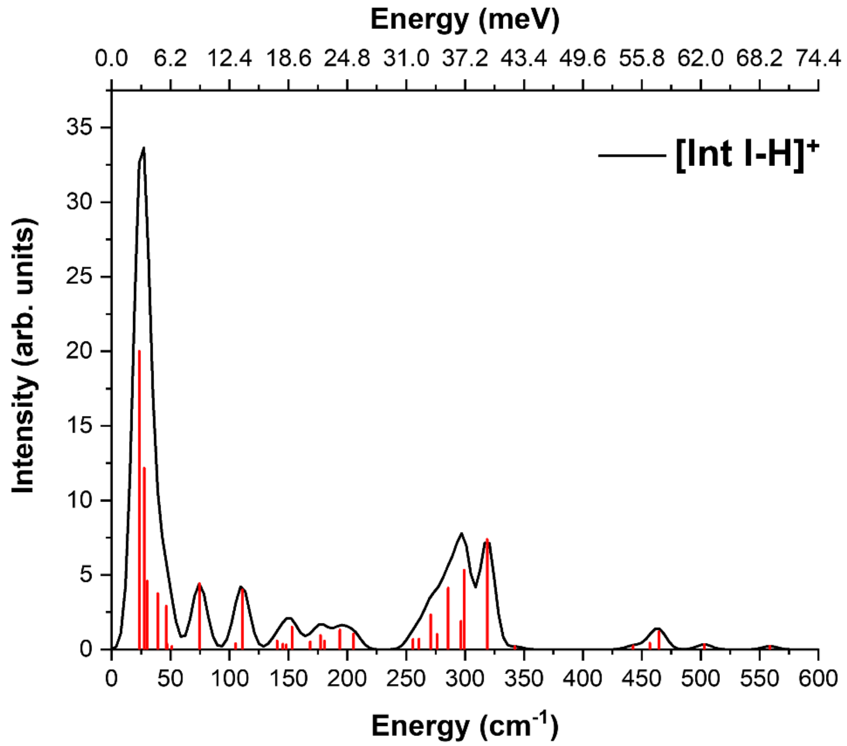

Figure S56. Calculated NRVS spectra for [**Int I-H**]$^{+}$.

Table S39. Selected computed vibrational frequencies for [**Int I-H**]$^{+}$ that are present in the NRVS spectrum. All energies are in cm^{-1}

Assignment	[**Int I-H**]$^{+}$ Fe(N+O)	**Int I** Exp
δ(Fe-O_{acac})	300/318	256/280/313
υs(Fe-O_{acac})	443/457	458
υas(Fe-O_{acac})	558	540

Multivariate Curve Resolution of Experimental UV-vis Absorption Spectroscopy:

A Multivariate Curve Resolution (MCR) of the experimental absorption spectra around the isobestic point (590 nm) (Figure S57 and S58) has been carried out which clearly brings out the intermediate that prevents the observation of clean isosbestic point for the converiosn of **Int I** to **Int II**. The variation of the formed intermediate through the series of spectra are also displayed as Singular Value Decomposition component (SVD) (Figure S58b).

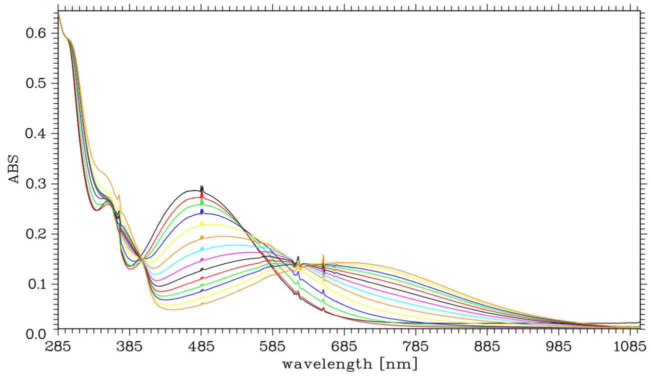

Figure S57. Experimental UV-Vis Absorption Spectra for Multivariate Curve Resolution (MCR) during the conversion of **Int I** to **Int II**.

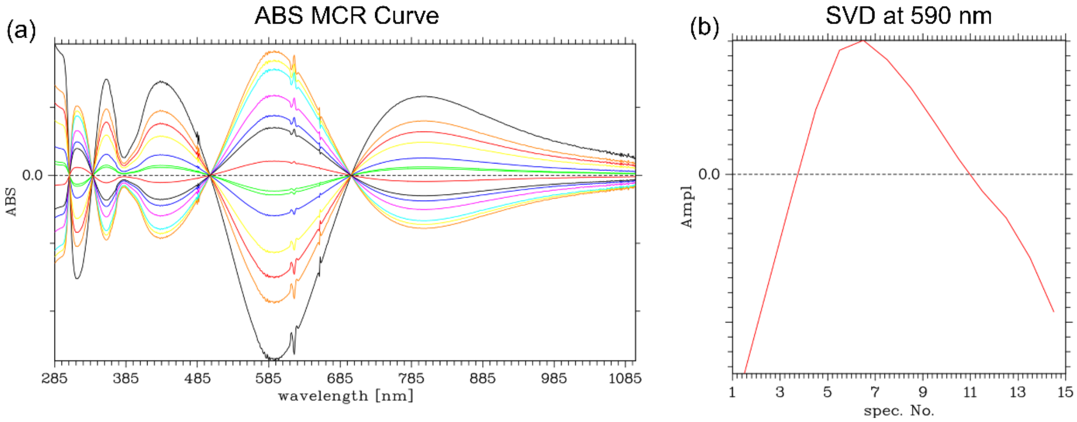

Figure S58. (a) Multivariate Curve Resolution (MCR) of experimental UV-Vis Absorption spectra around the isobestic point (590 nm) for the conversion of **Int I** to **Int II**.

Our calculated energy profile (Figure 9) depicts that conversion of **Int I** to **Int II** goes via a transient intermediate **I-R**, and further kinetic simulations of the experimental results

reveal that the maximal concentration of **I-R** is too low to allow its detection, which agrees well with the experimental result. So, the intermediate monitored affecting the isobestic point of conversion of **Int I** to **Int II** in UV-Vis Kinetics could be attributed to the transient intermediate **I-R** in the computed reaction pathway.

References

1. Makai, S.; Falk, E.; Morandi, B., Preparation of O-Pivaloyl Hydroxylamine Triflic Acid. *Org. Synth.* **2020,** *97*, 207.
2. Stoll, S.; Schweiger, A., EasySpin, a comprehensive software package for spectral simulation and analysis in EPR. *J. Magn. Reson.* **2006,** *178* (1), 42-55.
3. Buckingham, D. A.; Gorges, R. C.; Henry, J. T., The polymeric nature of Bis(acetylacetonato)-, Bis(trifluoroacetylacetonato)-, Bis(hexafluoroacetylacetonato)-, and Bis(2,2,6,6-tetramethylheptane-3,5-dionato)-iron(II). *Aust. J. Chem.* **1967,** *20* (2), 281-296.
4. Legnani, L.; Morandi, B., Direct Catalytic Synthesis of Unprotected 2-Amino-1-Phenylethanols from Alkenes by Using Iron(II) Phthalocyanine. *Angew. Chem. Int. Ed.* **2016,** *55* (6), 2248-2251; *Angew. Chem*. **2016**, *128* (6), 2288 –2292.
5. van Leest, N. P.; Tepaske, M. A.; Venderbosch, B.; Oudsen, J.-P. H.; Tromp, M.; van der Vlugt, J. I.; de Bruin, B., Electronically Asynchronous Transition States for C–N Bond Formation by Electrophilic [CoIII(TAML)]-Nitrene Radical Complexes Involving Substrate-to-Ligand Single-Electron Transfer and a Cobalt-Centered Spin Shuttle. *ACS Catalysis* **2020,** *10* (14), 7449-7463.
6. Jiang, X.-K., Establishment and Successful Application of the σJJ• Scale of Spin-Delocalization Substituent Constants. *Acc. Chem. Res.* **1997,** *30* (7), 283-289.
7. Weisser, J. T.; Nilges, M. J.; Sever, M. J.; Wilker, J. J., EPR Investigation and Spectral Simulations of Iron−Catecholate Complexes and Iron−Peptide Models of Marine Adhesive Cross-Links. *Inorg. Chem.* **2006,** *45* (19), 7736-7747.
8. Neese, F., Software update: the ORCA program system, version 4.0. *WIREs Computational Molecular Science* **2018,** *8* (1), e1327.
9. Becke, A. D., Density-functional thermochemistry. III. The role of exact exchange. *The Journal of Chemical Physics* **1993,** *98* (7), 5648-5652.
10. Lee, C.; Yang, W.; Parr, R. G., Development of the Colle-Salvetti correlation-energy formula into a functional of the electron density. *PRB* **1988,** *37* (2), 785-789.
11. Stephens, P. J.; Devlin, F. J.; Chabalowski, C. F.; Frisch, M. J., Ab Initio Calculation of Vibrational Absorption and Circular Dichroism Spectra Using Density Functional Force Fields. *J. Phys. Chem.* **1994,** *98* (45), 11623-11627.
12. Vosko, S. H.; Wilk, L.; Nusair, M., Accurate spin-dependent electron liquid correlation energies for local spin density calculations: a critical analysis. *Canadian Journal of Physics* **1980,** *58* (8), 1200-1211.
13. Grimme, S.; Antony, J.; Ehrlich, S.; Krieg, H., A consistent and accurate ab initio parametrization of density functional dispersion correction (DFT-D) for the 94 elements H-Pu. *J. Chem. Phys.* **2010,** *132* (15), 154104.

14. Grimme, S.; Ehrlich, S.; Goerigk, L., Effect of the damping function in dispersion corrected density functional theory. *J. Comput. Chem.* **2011,** *32* (7), 1456-1465.
15. Weigend, F.; Ahlrichs, R., Balanced basis sets of split valence, triple zeta valence and quadruple zeta valence quality for H to Rn: Design and assessment of accuracy. *Phys. Chem. Chem. Phys.* **2005,** *7* (18), 3297-3305.
16. Eichkorn, K.; Treutler, O.; Öhm, H.; Häser, M.; Ahlrichs, R., Auxiliary basis sets to approximate Coulomb potentials. *Chemical Physics Letters* **1995,** *240* (4), 283-290.
17. Neese, F., An improvement of the resolution of the identity approximation for the formation of the Coulomb matrix. *J. Comput. Chem.* **2003,** *24* (14), 1740-1747.
18. Neese, F.; Wennmohs, F.; Hansen, A.; Becker, U., Efficient, approximate and parallel Hartree-Fock and hybrid DFT calculations. A ‘chain-of-spheres’ algorithm for the Hartree-Fock exchange. *Chemical Physics* **2009,** *356* (1), 98-109.
19. Weigend, F., Accurate Coulomb-fitting basis sets for H to Rn. *Phys. Chem. Chem. Phys.* **2006,** *8* (9), 1057-1065.
20. Grimme, S., Supramolecular Binding Thermodynamics by Dispersion-Corrected Density Functional Theory. *Chem. Eur. J.* **2012,** *18* (32), 9955-9964.
21. Barone, V.; Cossi, M., Quantum Calculation of Molecular Energies and Energy Gradients in Solution by a Conductor Solvent Model. *J. Phys. Chem. A* **1998,** *102* (11), 1995-2001.
22. Garcia-Ratés, M.; Neese, F., Effect of the Solute Cavity on the Solvation Energy and its Derivatives within the Framework of the Gaussian Charge Scheme. *J Comput Chem* **2020,** *41* (9), 922-939.
23. York, D. M.; Karplus, M., A Smooth Solvation Potential Based on the Conductor-Like Screening Model. *J. Phys. Chem. A* **1999,** *103* (50), 11060-11079.
24. Mills, G.; Jónsson, H.; Schenter, G. K., Reversible work transition state theory: application to dissociative adsorption of hydrogen. *Surface Science* **1995,** *324* (2), 305-337.
25. Henkelman, G.; Jónsson, H., Improved tangent estimate in the nudged elastic band method for finding minimum energy paths and saddle points. *The Journal of Chemical Physics* **2000,** *113* (22), 9978-9985.
26. Henkelman, G.; Uberuaga, B. P.; Jónsson, H., A climbing image nudged elastic band method for finding saddle points and minimum energy paths. *The Journal of Chemical Physics* **2000,** *113* (22), 9901-9904.
27. Hohenberg, P.; Kohn, W., Inhomogeneous Electron Gas. *PR* **1964,** *136* (3B), B864-B871.
28. Runge, E.; Gross, E. K. U., Density-Functional Theory for Time-Dependent Systems. *PRL* **1984,** *52* (12), 997-1000.
29. Martin, R. L., Natural transition orbitals. *J. Chem. Phys.* **2003,** *118* (11), 4775-4777.
30. Wüllen, C. v., Molecular density functional calculations in the regular relativistic approximation: Method, application to coinage metal diatomics, hydrides, fluorides and chlorides, and comparison with first-order relativistic calculations. *The Journal of Chemical Physics* **1998,** *109* (2), 392-399.
31. Lenthe, E. v.; Baerends, E. J.; Snijders, J. G., Relativistic regular two-component Hamiltonians. *The Journal of Chemical Physics* **1993,** *99* (6), 4597-4610.

32. Pantazis, D. A.; Chen, X.-Y.; Landis, C. R.; Neese, F., All-Electron Scalar Relativistic Basis Sets for Third-Row Transition Metal Atoms. *J. Chem. Theory Comput.* **2008,** *4* (6), 908-919.
33. Petrenko, T.; Neese, F., Analysis and prediction of absorption band shapes, fluorescence band shapes, resonance Raman intensities, and excitation profiles using the time-dependent theory of electronic spectroscopy. *J. Chem. Phys.* **2007,** *127* (16), 164319.
34. Petrenko, T.; Neese, F., Efficient and automatic calculation of optical band shapes and resonance Raman spectra for larger molecules within the independent mode displaced harmonic oscillator model. *The Journal of Chemical Physics* **2012,** *137* (23), 234107.
35. Weinhold, F.; Landis, C. R., NATURAL BOND ORBITALS AND EXTENSIONS OF LOCALIZED BONDING CONCEPTS. *Chemistry Education Research and Practice* **2001,** *2* (2), 91-104.
36. Glendening, E. D.; Landis, C. R.; Weinhold, F., NBO 7.0: New vistas in localized and delocalized chemical bonding theory. *Journal of Computational Chemistry* **2019,** *40* (25), 2234-2241.
37. Römelt, M.; Ye, S.; Neese, F., Calibration of Modern Density Functional Theory Methods for the Prediction of 57Fe Mössbauer Isomer Shifts: Meta-GGA and Double-Hybrid Functionals. *Inorg. Chem.* **2009,** *48* (3), 784-785.
38. Neese, F., Prediction and interpretation of the 57Fe isomer shift in Mössbauer spectra by density functional theory. *Inorganica Chimica Acta* **2002,** *337*, 181-192.
39. Pracht, P.; Bohle, F.; Grimme, S., Automated exploration of the low-energy chemical space with fast quantum chemical methods. *Phys. Chem. Chem. Phys.* **2020,** *22* (14), 7169-7192.
40. Grimme, S.; Bannwarth, C.; Shushkov, P., A Robust and Accurate Tight-Binding Quantum Chemical Method for Structures, Vibrational Frequencies, and Noncovalent Interactions of Large Molecular Systems Parametrized for All spd-Block Elements (Z = 1-86). *J. Chem. Theory Comput.* **2017,** *13* (5), 1989-2009.
41. Bannwarth, C.; Ehlert, S.; Grimme, S., GFN2-xTB--An Accurate and Broadly Parametrized Self-Consistent Tight-Binding Quantum Chemical Method with Multipole Electrostatics and Density-Dependent Dispersion Contributions. *J. Chem. Theory Comput.* **2019,** *15* (3), 1652-1671.
42. Neese, F., Prediction and interpretation of the 57Fe isomer shift in Mössbauer spectra by density functional theory. *Inorg. Chim. Acta* **2002,** *337*, 181-192.
43. Neese, F., Importance of Direct Spin−Spin Coupling and Spin-Flip Excitations for the Zero-Field Splittings of Transition Metal Complexes: A Case Study. *J. Am. Chem. Soc.* **2006,** *128* (31), 10213-10222.
44. Kurian, R.; Filatov, M., DFT Approach to the Calculation of Mössbauer Isomer Shifts. *J. Chem. Theory Comput.* **2008,** *4* (2), 278-285.
45. Liu, T.; Lovell, T.; Han, W.-G.; Noodleman, L., DFT Calculations of Isomer Shifts and Quadrupole Splitting Parameters in Synthetic Iron−Oxo Complexes: Applications to Methane Monooxygenase and Ribonucleotide Reductase. *Inorg. Chem.* **2003,** *42* (17), 5244-5251.
46. Van Stappen, C.; Davydov, R.; Yang, Z.-Y.; Fan, R.; Guo, Y.; Bill, E.; Seefeldt, L. C.; Hoffman, B. M.; DeBeer, S., Spectroscopic Description of the E1 State of Mo

Nitrogenase Based on Mo and Fe X-ray Absorption and Mössbauer Studies. *Inorg. Chem.* **2019,** *58* (18), 12365-12376.
47. Bjornsson, R.; Neese, F.; DeBeer, S., Revisiting the Mössbauer Isomer Shifts of the FeMoco Cluster of Nitrogenase and the Cofactor Charge. *Inorg. Chem.* **2017,** *56* (3), 1470-1477.
48. Schulz, C. E.; Castillo, R. G.; Pantazis, D. A.; DeBeer, S.; Neese, F., Structure–Spectroscopy Correlations for Intermediate Q of Soluble Methane Monooxygenase: Insights from QM/MM Calculations. *J. Am. Chem. Soc.* **2021,** *143* (17), 6560-6577.
49. Yamaguchi, K.; Takahara, Y.; Fueno, T. In *Ab-Initio Molecular Orbital Studies of Structure and Reactivity of Transition Metal-OXO Compounds*, Springer: 1986; 155-184.
50. Soda, T.; Kitagawa, Y.; Onishi, T.; Takano, Y.; Shigeta, Y.; Nagao, H.; Yoshioka, Y.; Yamaguchi, K., Ab initio computations of effective exchange integrals for H–H, H–He–H and Mn2O2 complex: comparison of broken-symmetry approaches. *Chem. Phys. Lett.* **2000,** *319* (3), 223-230.
51. Eyring, H., The Activated Complex in Chemical Reactions. *The Journal of Chemical Physics* **1935,** *3* (2), 107-115.

XYZ Coordinates of important species in Figure 9

35

1 (Precursor) Trans-isomer

Fe	-0.00487737994253	0.00762900630488	0.00674219415211
O	0.16241734464471	-0.03908092229989	2.28831831751870
O	-0.13170345002244	0.05018865161143	-2.27202248071802
O	1.48167858524646	-1.39667859534721	-0.03114129103220
O	-1.49574662083112	1.40946462700679	0.00744686398419
O	-1.48320716564432	-1.40769387167915	-0.02771744191757
O	1.48061937485708	1.41915856526992	0.07553169369160
C	-2.75111159523854	1.23986129060685	-0.06877076672770
C	-3.39340195809608	-0.00681316131723	-0.13298539127604
C	-2.73992959701307	-1.24906325928028	-0.09494351683594
C	-3.58063345655843	2.50043439222007	-0.07889822151334
C	-3.55827644649941	-2.51625463284375	-0.11832229780232
C	2.73704115546630	1.25230427261694	0.13826280949309
C	3.38665628571142	0.00789794581482	0.11873156373540
C	2.73871464304316	-1.23412868373866	0.01594936483087
C	3.55869225559759	2.51492318440681	0.22783011122885

C	3.56322587594434	-2.49504976249556	-0.05915743988674
H	-4.47202626681075	-0.01092254083789	-0.19968842946921
H	-3.18346122307055	3.18979338146602	-0.82555100316803
H	-3.48780932665565	2.99188784106543	0.89284753402721
H	-4.63324890378547	2.30933835307886	-0.28201133087458
H	-3.11252661422714	-3.22396985765528	-0.81864344154886
H	-4.59868952299018	-2.33877225904040	-0.38655735964010
H	-3.52196456146857	-2.97722550048168	0.87244793484786
H	4.46602144016280	0.00582915875148	0.16843595964506
H	3.13623016377061	3.16678149487357	0.99415013819700
H	3.48873172740335	3.05098246392410	-0.72223262516845
H	4.60663203172769	2.32051415016085	0.45145084480934
H	4.61171444936866	-2.32713541553667	0.18241391891216
H	3.49301701900608	-2.89994779792059	-1.07234809036523
H	3.14490143456816	-3.24403064846737	0.61512091716870
H	-0.75061011247353	-0.65040645933265	-2.51706799609386
H	-0.57778711030115	0.87867149820120	-2.49286359100264
H	0.65345634565746	-0.84654797086487	2.48990537064669
H	0.75946955325307	0.68841751355912	2.50691487475198

35

1 (Precursor) *cis*-Isomer

Fe	0.10895276018927	0.45010378048990	0.54622858944591
O	0.55146353202251	1.46548892528204	2.54999599516475
O	-0.18555736576723	2.77699003775613	-0.08516498491938
O	-0.75332834401697	-0.23320477831644	-1.19519312837141
O	1.08255768122740	-1.27427915055118	1.01869792247871
O	1.85307460371028	1.07879614757609	-0.33581005920704
O	-1.77275869692930	0.52683965164841	1.32261842592192
C	2.13489570688574	-1.73013004978561	0.48182274406558
C	2.97720026279079	-1.00486359826590	-0.38242098060843
C	2.79892950789305	0.33164282535639	-0.75020300648594

C	2.48590487225358	-3.16154041440535	0.80790202216972
C	3.77656561822546	0.97663814062647	-1.69869799315253
C	-2.87120668459778	0.15344458407090	0.79121885236791
C	-3.00897715181155	-0.37626496464536	-0.49466215094236
C	-1.95337378026603	-0.55936854776458	-1.41149585540114
C	-4.09679450910320	0.32423454787557	1.65138496579169
C	-2.23421267394029	-1.18432863957607	-2.75728255227216
H	3.83152348814377	-1.52160252843565	-0.79329216413790
H	2.65237493068616	-3.25950602585427	1.88385627860190
H	3.37381581647896	-3.50212224891196	0.27915959950829
H	1.64192072141743	-3.80424711241466	0.54842433121700
H	3.22371274435099	1.45068589637695	-2.51239561475998
H	4.48956647914170	0.26492462061868	-2.11088738250310
H	4.32302656332767	1.76344265343898	-1.17167368573343
H	-3.99909514855827	-0.67654225171482	-0.80431675129491
H	-3.95068362184740	-0.20296130822537	2.59657154382785
H	-5.00017507169038	-0.04271845620000	1.16753131888551
H	-4.22275805814622	1.38380337651270	1.88937503272163
H	-1.65385082536682	-2.10569096477541	-2.84677062644492
H	-1.88844408697947	-0.51073272966284	-3.54394582360608
H	-3.28889165911606	-1.41080999332013	-2.90706749080370
H	-0.90244036336546	2.81997289903625	-0.72979609934305
H	0.63115208497094	2.67139287173182	-0.60127493940035
H	-0.32819019623089	1.36985919399147	2.94375755075535
H	0.63312382731761	2.40652279133684	2.34391570186408

47

Int I (Fe(N+O)

Fe	-0.69590297450722	0.27500979090863	0.21470824082889
N	-0.16700370267335	-1.17990102297188	1.48635867597023
H	-0.31549634361474	-1.13926380047008	2.49016122470950
O	1.22534096932884	-1.54604540703392	1.41796225094049

C	1.76122083934281	-1.23314668382620	0.26225414841668
O	1.12307123848376	-0.61614878820853	-0.59216979502192
C	3.20039283284582	-1.66204574544523	0.05382585396415
C	3.75396850346602	-2.42651919383196	1.25893537346999
H	4.78648078449675	-2.71584672896677	1.05566178467300
H	3.74117997722813	-1.81085463657932	2.15844946267874
H	3.17899955732383	-3.33150888722958	1.45603863144304
C	3.23254055610325	-2.53969236204044	-1.21059741240908
H	2.86661806389706	-1.98326947505808	-2.07291519270524
H	4.25894099030944	-2.85557836289754	-1.40491718574950
H	2.61649845716746	-3.43180374802484	-1.08447751793574
C	4.02047340952715	-0.38151761353060	-0.19118809885053
H	4.05450929685296	0.23848499311387	0.70669999743146
H	3.58732026153271	0.20676124634649	-0.99909492820976
H	5.04089279188293	-0.65595373462584	-0.46441631790736
O	-2.32666589409210	0.74563633066184	1.23842838769664
C	-3.47307375246060	0.17488302422506	1.20766232450012
C	-3.87703491354521	-0.74506938356372	0.24620716727473
C	-3.09014696897389	-1.15163043268443	-0.84854559139496
O	-1.89146706771671	-0.80060507439773	-1.03756479965737
C	-3.71265544567091	-2.03090256270672	-1.89977798063717
H	-4.47962335228161	-2.67857416761080	-1.47629791281003
H	-4.18710383289501	-1.39269571982789	-2.65138685087660
H	-2.94534793721302	-2.62518249411907	-2.39394841482589
H	-4.87905768512739	-1.14098924500856	0.31714119296385
C	-4.41753917093580	0.57086808955453	2.30702780821510
H	-3.96126225033360	0.32629484835905	3.26925496058118
H	-4.57069991694227	1.65214813238927	2.28304889767012
H	-5.37781367251071	0.06579548462945	2.22502057739576
O	-0.47535487321082	1.59293037805468	-1.30437584442957
C	0.42996924791936	2.46973775357394	-1.48372205280431

C	1.32596855511397	2.90174859745962	-0.50133680313676
C	1.28718223079397	2.47042992887611	0.83216090745634
O	0.48489193086724	1.59854852335253	1.28404381097091
C	2.23702415887785	3.07574906555956	1.83255270345961
H	1.66277230707754	3.66506257795282	2.55232535713870
H	2.73547400808367	2.27922473757824	2.38779931697180
H	2.97971701574037	3.71655319671460	1.36088087606148
H	2.05572367324866	3.64965615865584	-0.77183693695317
C	0.51216616397401	3.05930128943835	-2.86649372233486
H	1.22245955809199	3.88186552530646	-2.92360538168901
H	0.81789974460852	2.27649390757749	-3.56544366394715
H	-0.47550736948120	3.40665169037131	-3.17576752859652

47

Int I (FeN)

Fe	1.04493504031916	0.19959658126964	-0.30420751550840
N	-0.16572496964627	-0.08929796026557	-1.82339170492659
H	-0.63525046661563	0.75805293321087	-2.14574503841243
O	-1.21867560284429	-0.95240364711061	-1.37743778422161
C	-2.31642995511678	-0.34142813919237	-0.87740051451393
O	-2.47325383889496	0.85599190203795	-0.92019744356525
C	-3.27068408506934	-1.35454462073673	-0.25726726218823
C	-3.77913585961818	-2.31353514707341	-1.34690752017615
H	-2.95848694108192	-2.86566335042837	-1.80447630491736
H	-4.46971811058593	-3.02939933884787	-0.89633996639832
H	-4.31253916666080	-1.76908092338825	-2.12883679690618
C	-2.50789312187715	-2.14570776404828	0.82027455062363
H	-2.06911151957508	-1.47809444491563	1.56317350083908
H	-3.19911517882934	-2.82202956945125	1.32676746408826
H	-1.70203939374931	-2.73002335589574	0.37956849777886
C	-4.44560585821512	-0.60063578680985	0.36739377986444
H	-5.11798689011813	-1.31302186011387	0.84855548541972

H	-5.00528005101482	-0.04783110182093	-0.38782666098147
H	-4.10108112883560	0.11270185826267	1.11607723956399
O	1.17606872032073	-1.68604105006729	0.37367987722186
C	2.21196441417689	-2.35174842601892	0.68822842921722
C	3.52081792005563	-1.94234948434178	0.40812933650728
C	3.84096569657752	-0.78194346540975	-0.30401436925850
O	2.99513903487788	0.08552424709805	-0.70686471731333
C	5.27614920434290	-0.52175331982384	-0.66801270341136
H	5.95912237308228	-1.15841642105424	-0.10927692666205
H	5.40948098240355	-0.72276323605675	-1.73530934434849
H	5.51851930903934	0.52724947284801	-0.49214515452362
H	4.33133815593762	-2.58521409597761	0.71587133480311
C	1.96536423084827	-3.66767837897252	1.37500045867760
H	1.25340295554048	-3.52151582596988	2.18844892453173
H	1.50726727631459	-4.35706444016623	0.66118427176208
H	2.88164150163191	-4.10964432403262	1.76247029274854
O	1.16946696862569	2.18993021420587	-0.58724159448777
C	0.43460274271946	3.09739020975441	-0.08007442304153
C	-0.43539697778129	2.89450414212401	0.99648405647315
C	-0.59415466931249	1.67525720667061	1.65079194257951
O	0.02881371448661	0.59853266937464	1.35046959692342
C	-1.54955482505490	1.55891587521953	2.80022374036804
H	-1.98852120431502	2.51663060607861	3.07289667561912
H	-2.34689625024970	0.86779007755879	2.51909666623928
H	-1.02946110252086	1.12838122517808	3.65791811067785
H	-1.01578430458032	3.73561249448401	1.34297913320494
C	0.54158450251285	4.46161138375136	-0.69742967993008
H	1.58094122567676	4.79455459349824	-0.65377926435036
H	0.25820811638067	4.39895959998996	-1.75157854189019
H	-0.09379210620753	5.19063788217488	-0.19980908649953

63

TS-1

Fe	-0.77712886133243	0.04936673939713	0.09109698649318
N	-0.19610350331810	-1.26922056324719	1.47496870064647
H	-0.36650223211488	-1.20095628765020	2.47277547131981
O	1.16288880098259	-1.72531391981533	1.43463548867707
C	1.72093565164228	-1.48454900876721	0.27534454592405
O	1.13126774068102	-0.86201406070840	-0.60685951299854
C	3.13471477939980	-2.01183396580487	0.09661693946971
C	3.64096633381800	-2.77234429693627	1.32567630728559
H	4.64432706760902	-3.15194254360666	1.12622634854194
H	3.68705352479127	-2.11583653355037	2.19385158528957
H	2.99701940567725	-3.61694022537966	1.56083850428788
C	3.16058389185879	-2.87563672639071	-1.14783875293764
H	2.67678068325776	-2.43639137688260	-2.01506929750051
H	4.14401262855211	-3.28847329268355	-1.36623164830285
H	2.50103155488974	-3.96021667296899	-0.88724237474470
C	4.03479795790672	-0.78154350284279	-0.18983424894053
H	4.06899176784219	-0.13673823148388	0.69041757006106
H	3.65137880289688	-0.20630228614053	-1.03050350341547
H	5.04560459558008	-1.12029742461103	-0.41863785533788
O	-2.38893207339147	0.40905608130849	1.20501671416701
C	-3.34295588926559	-0.42139852835143	1.41315142881625
C	-3.71004098015976	-1.43753757042997	0.53390556193890
C	-3.06809846987233	-1.67772689618150	-0.69541387792000
O	-1.99089429278875	-1.12082186830231	-1.05515524387239
C	-3.70649152593901	-2.61632944818665	-1.68317818477663
H	-4.40418682372195	-3.29954010566542	-1.20138672624801
H	-4.25974978351061	-2.01760794755697	-2.41277861402303
H	-2.94534041792096	-3.17587188347530	-2.22259165779379
H	-4.57963495938457	-2.03005011915357	0.77462215555266
C	-4.08883914483138	-0.24123756347504	2.70690436105409

H	-3.41287886280319	-0.49579943082526	3.52797890874682
H	-4.37423170546957	0.80470412624436	2.83256357532606
H	-4.97047280600799	-0.87620265766478	2.76847180488329
O	-0.51285174169396	1.10729098911792	-1.61522791557910
C	0.41923816254894	1.92766071959623	-1.89166229848481
C	1.24231965070069	2.53852732370001	-0.94216661364485
C	1.06892803900509	2.38606906380733	0.43936134888659
O	0.20108351680569	1.62972073004807	0.97526238778194
C	1.94602442341532	3.16439200216266	1.38349362559372
H	1.32038338301873	3.78590639463876	2.02807757281544
H	2.48385731421613	2.46566241758629	2.02853572117936
H	2.66016297037935	3.79363779397003	0.85548167342191
H	2.00734252945977	3.21545817203605	-1.29040632472341
C	0.61592098368836	2.23661123846991	-3.35028137736191
H	1.38073224438149	2.99292850079887	-3.51372356532970
H	0.90325531625303	1.31906392042696	-3.86996125905381
H	-0.32830194950083	2.57544861481207	-3.78110682781671
C	0.42698997375834	-5.16289724945854	-2.25284170997025
C	-0.16921403242287	-3.78205749594741	-1.94116003759319
C	-0.70330135330216	-6.10904202579422	-2.67081797535020
C	1.45647290230299	-5.07100221982211	-3.39444697124083
C	1.10900020988958	-5.72636608031923	-1.00763448867121
O	2.07844175287487	-5.04956806615503	-0.40014029169620
O	0.82156560732532	-6.79458844452973	-0.50099325987928
H	2.29259225638921	-4.42016946653684	-3.14695992066949
H	-0.73537357449791	-3.43225964006112	-2.80465912434204
H	0.58336482774677	-3.03193589429813	-1.72700878019031
H	-0.84575833125066	-3.82883814314750	-1.08792559143344
H	-1.19541648874500	-5.70412277876048	-3.55696621803192
H	-1.44575472994391	-6.20666103449483	-1.87873907036003
H	-0.32425520384974	-7.10300976727568	-2.90855223458113

H	0.96878632985792	-4.66576057422957	-4.28295988622079
H	1.85479215563604	-6.05712100855145	-3.64052204712349

46

I-R This structure was obtained with ORCA 5.0. The Gibbs energy was calculated with ORCA 4.2.1 as detailed in the section "Computational details".

Fe	-0.02018501824266	0.68053994551381	0.30007160074129
N	0.43294882660667	-0.36869852942853	1.79518900306181
H	1.08330446868558	0.15378727146043	2.38439147395856
O	1.22680263617305	-1.53400545002816	1.41609165500556
C	1.94568886340318	-1.37992045955688	0.30846728849609
O	1.92474365570837	-0.35177196328041	-0.35033688649735
C	2.80323394682689	-2.60667535682108	-0.02543625125658
C	1.98328438938315	-3.89335184002866	0.18236327678469
H	2.59214738802784	-4.75156136896727	-0.10470215047859
H	1.68220345710717	-4.01066306412922	1.22178958482916
H	1.08623769032167	-3.88015031742468	-0.43873403257766
C	3.27493313438626	-2.50109378115955	-1.48030718590280
H	3.85843791783342	-1.59566488877385	-1.63982385260521
H	3.89597573948093	-3.36545984551845	-1.71753710869881
H	2.42342893048128	-2.48396887373475	-2.16139839746286
C	3.96553615800318	-2.55261479467938	0.91908728977381
H	3.86171070928547	-2.92468045800234	1.92927328254396
H	-1.29511178006812	3.32968005237377	-3.08258530643929
H	4.85576911592341	-1.99975585308343	0.65107145986390
O	-1.86549981838164	1.27632544097353	0.95313558246449
C	-2.88293237493415	0.52815656728828	1.03810728355767
C	-2.97321979314950	-0.76036335370353	0.48451354115808
C	-1.97973735588462	-1.36660561184534	-0.28476871177242
O	-0.84537854779821	-0.85345764123970	-0.56258104394173
C	-2.21884958168473	-2.73149963634206	-0.87160981500510
H	-3.22792993896095	-3.09042566966847	-0.67976667592297

H	-2.03980826115466	-2.70064009543134	-1.94798946416595
H	-1.49999288858807	-3.43262762630850	-0.44059657130715
H	-3.89405453799837	-1.30521891405673	0.62740142748199
C	-4.06467603996941	1.09581990270441	1.77971429571661
H	-3.76825683914518	1.31166508685098	2.80868601512623
H	-4.35483383112567	2.04273313474824	1.31982687801874
H	-4.91671280633738	0.41879157609663	1.78448589557338
O	-0.14505517652953	1.69468075523272	-1.36321456046438
C	0.08444426791712	2.93360527419073	-1.51449466781233
C	0.61095360556489	3.77558988680523	-0.52592219351239
C	1.00372329912175	3.34339575120341	0.73922687691109
O	0.89377105856090	2.14972758847324	1.18342501489074
C	1.62618727301247	4.31492359409828	1.70177113112872
H	1.02267373751712	4.35000769277742	2.61132559819791
H	2.61800576356580	3.95475376207493	1.98342727679477
H	1.70884089358166	5.31496235702150	1.28175825562241
H	0.76449249775476	4.81417495842122	-0.77527889097211
C	-0.23445656258643	3.48710922200951	-2.87502251488852
H	-0.00169903159492	4.54672722665123	-2.95556260657135
H	0.32896791497562	2.93118005024368	-3.62720916429634

46

TS-2 This structure was obtained with ORCA 5.0. The Gibbs energy was calculated with ORCA 4.2.1 as detailed in the section "Computational details".

Fe	-0.43912161797143	0.20396617938368	0.38829441578916
N	-0.46782013514093	-0.80491895131685	1.80537465744609
H	0.10312719785086	-0.38166462215262	2.54821236543282
O	1.37260406246298	-2.13171021907586	1.45631389095262
C	1.81318670410542	-1.61457406165320	0.44246648501587
O	1.33274144303730	-0.73257239277935	-0.29701107114118
C	3.33349195482394	-2.18023690257530	-0.04171741274877
C	3.71253228678518	-3.38728113242417	0.80918994705162

H	4.66999169084824	-3.78155499434766	0.46271025333268
H	3.80717211602642	-3.11638794444596	1.85967544798913
H	2.96312567058631	-4.17301554292203	0.72340657844564
C	3.14443325567574	-2.54462202082849	-1.52088876971157
H	2.89113674973581	-1.66802246750535	-2.11292806203497
H	4.07352994911198	-2.97508902666781	-1.89867290539392
H	2.35191083968948	-3.28490282857507	-1.63520547847488
C	4.15128137120856	-1.02185187969932	0.19265200976522
H	4.68947002874548	-0.90643094248142	1.12482873202970
H	-0.76847027608470	3.32711252417738	-2.95301668039615
H	4.11839427445537	-0.17966318392602	-0.48553420180210
O	-2.16309570034392	1.22186879534915	0.85978166579617
C	-3.33084440780524	0.73162993213340	0.82810908051518
C	-3.67842059158484	-0.47772930591124	0.20498956916942
C	-2.80210846598389	-1.27349703257539	-0.53757062752910
O	-1.56351097956368	-1.03058758428822	-0.71029036630808
C	-3.31621793315571	-2.51249960005450	-1.21855842177426
H	-4.39136065607144	-2.63323821337117	-1.10172480374695
H	-3.06541740580535	-2.47024984360347	-2.28025713618641
H	-2.80690397908095	-3.38292693016926	-0.79809012293357
H	-4.71079543365610	-0.78833201660740	0.25906435030003
C	-4.39900931543557	1.54325516564084	1.51277386313180
H	-4.17169793027409	1.59887595788013	2.58008527420527
H	-4.38182104514451	2.56275949745147	1.12303369930017
H	-5.39169884225134	1.11803891188513	1.37867377835466
O	-0.25743611100585	1.39295817265799	-1.22617206760838
C	0.34456375439042	2.49892636124078	-1.33960860335272
C	1.04608825039052	3.13964823491592	-0.30534909197531
C	1.16658528306277	2.64014134980867	0.98954815500673
O	0.66640226031079	1.54417335698175	1.41187850622445
C	1.94388847747519	3.41598896275393	2.01835487682504

H	1.29250520327487	3.63572066887163	2.86689470952261
H	2.76324248637804	2.79657210553578	2.38991970328571
H	2.34492468505443	4.34522007112278	1.61883705877962
H	1.51985852680785	4.08346382862798	-0.52775136436847
C	0.27750559782574	3.14286521378011	-2.69853106434825
H	0.83018837161887	4.07921183211360	-2.74021808057606
H	0.67547833462073	2.44948251764501	-3.44218274125632

31

Int II (TBP-Neq)

Fe	-0.021650	-0.050389	-0.573867
N	-0.173313	0.041293	-2.421837
H	-0.736296	0.781824	-2.854406
O	-0.807114	-1.673653	0.234436
C	-1.985860	-1.914538	0.661760
C	-3.001311	-0.962860	0.754506
C	-2.831373	0.388391	0.416129
O	-1.762383	0.880868	-0.056968
C	-3.959599	1.360411	0.623520
H	-4.235463	1.801587	-0.337232
H	-4.833447	0.892967	1.072553
H	-3.609894	2.173868	1.262370
H	-3.958622	-1.273811	1.143743
C	-2.220185	-3.333033	1.100087
H	-3.246284	-3.505285	1.420146
H	-1.541880	-3.566111	1.923839
H	-1.967620	-4.006740	0.278770
O	1.753808	-1.022756	-0.370040
C	2.833953	-0.640513	0.168056
C	3.020963	0.622320	0.754060
C	2.026671	1.595615	0.819682
O	0.844771	1.460683	0.351105

C	2.288827	2.916411	1.486165
H	1.612576	3.024596	2.337198
H	3.317227	3.011323	1.830163
H	2.055127	3.720436	0.785559
H	3.984414	0.844203	1.186432
C	3.957933	-1.638965	0.161231
H	3.626384	-2.544457	0.674759
H	4.856782	-1.256454	0.640252
H	4.183361	-1.920098	-0.869821

31

Int II (SQP-Nax)

Fe	0.020190	0.060451	0.416971
N	0.427142	-0.059032	2.128658
H	1.379931	-0.068443	2.506437
O	1.600717	-0.853005	-0.354720
C	2.750900	-0.385742	-0.648915
C	3.055174	0.976318	-0.709984
C	2.104212	1.983422	-0.499879
O	0.909527	1.768064	-0.123573
C	2.451763	3.425302	-0.745058
H	3.490103	3.558618	-1.044687
H	1.799426	3.813431	-1.529936
H	2.245550	4.002487	0.158221
H	4.053459	1.264200	-1.003821
C	3.794735	-1.410116	-0.996773
H	3.673376	-2.289140	-0.364769
H	4.802850	-1.011265	-0.895122
H	3.648146	-1.728582	-2.033624
O	-1.690258	1.014473	0.068454
C	-2.811795	0.595195	-0.369285
C	-3.107712	-0.745255	-0.615609

C	-2.177388	-1.786128	-0.465532
O	-0.978989	-1.630116	-0.078934
C	-2.577542	-3.193556	-0.822162
H	-2.035748	-3.900527	-0.196150
H	-3.651371	-3.349341	-0.724331
H	-2.293080	-3.384713	-1.861907
H	-4.095307	-0.993037	-0.974707
C	-3.837619	1.665472	-0.619285
H	-3.401275	2.425243	-1.270832
H	-4.082806	2.155252	0.326259
H	-4.747221	1.274455	-1.070490

31

Int II (TBP-Nax)

Fe	-0.07783114995510	-0.79375564366574	0.16057538849474
N	0.29727000099065	-2.51185248311586	0.17537433047931
H	-0.57451804666942	-3.05437349262509	0.12904959501664
O	1.12157753344400	-0.36091049348345	-1.34977695779838
C	2.34197104161996	-0.02916132582858	-1.23600358767390
C	3.02268640142412	0.14008420755650	-0.02071658203378
C	2.46463366537052	0.02393699083259	1.25673969833247
O	1.25896903712123	-0.30348347368864	1.50844506989491
C	3.31514903654306	0.29507774022825	2.46740932659808
H	2.78093901202874	0.96920475322924	3.13870765138247
H	3.47421403823188	-0.64236331363326	3.00583671101572
H	4.27972591598621	0.72615057518183	2.20695250741495
H	4.06086587064262	0.43232900543434	-0.07745269422103
C	3.06656691705444	0.24068556763572	-2.52838912299212
H	4.14178224577943	0.09792936486511	-2.42850345408779
H	2.88061422907217	1.27806880851876	-2.82401129991593
H	2.67593532801611	-0.40359864230675	-3.31406837098061
O	-1.95413248860742	-1.22689633823460	0.10209128980231

C	-2.98246508706239	-0.45814941781368	-0.00513676199043
C	-2.95067365954168	0.92559416612732	-0.06046015769848
C	-1.75901689815578	1.67258570955086	0.00034988782952
O	-0.61547947725412	1.15738420809348	0.13523623603569
C	-1.80691218816679	3.17089703579715	-0.09573397383784
H	-1.45428382334917	3.59906612656813	0.84581468646566
H	-1.11810381978474	3.49897300911393	-0.87543733311407
H	-2.80751715282241	3.54084913264493	-0.31130291665467
H	-3.88501369367941	1.45553541603995	-0.16409754512585
C	-4.28796612019745	-1.19217064964693	-0.09317036679595
H	-4.30043252473717	-1.78298515820221	-1.01260338303179
H	-5.13747662216490	-0.51251341672895	-0.08997597364749
H	-4.36911852117718	-1.89108896844435	0.74085810283763

31

Int II (SQP-Neq)

Fe	-0.111900	0.908919	0.823658
O	0.727316	0.574696	-0.951875
C	1.819815	-0.043310	-1.140141
C	2.732730	-0.375514	-0.126973
C	2.553314	-0.126288	1.235975
O	1.530249	0.434594	1.759789
C	3.622124	-0.534751	2.212310
H	3.188529	-1.202685	2.959110
H	4.457061	-1.029654	1.720630
H	3.987059	0.349523	2.740553
H	3.645318	-0.869859	-0.423071
C	2.093352	-0.451165	-2.562157
H	1.844933	0.371878	-3.232777
H	3.124137	-0.766988	-2.714735
H	1.433075	-1.285452	-2.813196
O	-1.950232	1.250315	0.215656

C	-2.728807	0.412436	-0.353368
C	-2.574433	-0.973618	-0.320611
C	-1.590773	-1.615410	0.439986
O	-0.670060	-1.008133	1.070902
C	-1.607066	-3.112387	0.566669
H	-1.817434	-3.381546	1.605050
H	-2.354573	-3.571744	-0.075946
H	-0.619043	-3.505247	0.318008
H	-3.305345	-1.581908	-0.830699
C	-3.897389	1.019392	-1.075256
H	-3.523621	1.725738	-1.818911
H	-4.515507	0.267556	-1.562135
H	-4.501954	1.586823	-0.363791
N	0.204585	2.612142	1.005121
H	1.198740	2.862847	0.944323

47

TS-3

Fe	1.26649887924058	-0.25940249835477	-0.82902535346284
O	0.84702479659424	0.29061635244749	1.04149927298008
C	0.35055015018766	1.38584536532709	1.47260943750823
C	0.20966331484751	2.54306790960479	0.71115133897284
C	0.69034969113207	2.67910883318962	-0.60465556772425
O	1.28224885008237	1.76337492865043	-1.24983300978453
C	0.50776000624313	3.99199062227217	-1.31997586285472
H	-0.15488701317384	3.83543294032957	-2.17471890170575
H	0.07979336885234	4.75750286368325	-0.67569370453506
H	1.46665390682209	4.33255592367135	-1.71469757883364
H	-0.25001799625045	3.40318831502776	1.17389687572232
C	-0.07752897459456	1.36507202344504	2.91407086704026
H	-0.56142952859600	2.29151747393612	3.21798841035369
H	-0.75321762436452	0.52497522864960	3.08261952298259

H	0.80167050890017	1.18988420060809	3.53930942563552
O	1.49140612668222	-2.12574303029259	-0.01735140827376
C	2.56922013560483	-2.67680913123205	0.35380071641037
C	3.84778250091437	-2.21146465211626	0.01065592112742
C	4.07891398926259	-1.14239318062334	-0.85653216114374
O	3.16938096310297	-0.40432044470429	-1.36930294243509
C	5.48886686854445	-0.81947089106255	-1.26603939396355
H	6.21725009778167	-1.42717674059232	-0.73343574325299
H	5.59522891584003	-0.99483657577513	-2.33994513342386
H	5.68761355830827	0.23815759930905	-1.08058326851791
H	4.70280135284673	-2.75726564709690	0.37950174840126
C	2.42295748939274	-3.92328027088838	1.18432157672702
H	1.79681137461203	-3.70267117417365	2.05081261802940
H	3.38103610160235	-4.31834000557722	1.51754850678651
H	1.90511160416147	-4.68145570538415	0.59217099970901
N	-0.06241236772564	-0.69235689201910	-2.04289565701469
H	-0.78637673435969	0.00346232792988	-2.24241702008123
C	-1.64404942166555	-2.11001985697355	-1.02855668049851
C	-2.02628316502659	-1.26338012321924	-0.04789783827946
C	-3.18285196610587	-0.37432218455269	-0.04085109565089
C	-3.30334390602351	0.56941130624524	0.99087958519882
C	-4.38372298753909	1.43843567290967	1.05200624110618
C	-5.37529749606898	1.38493616913598	0.07817720375914
C	-5.27502462842599	0.45119421573334	-0.95298182587089
C	-4.19536477042607	-0.41696499749566	-1.01362467875269
H	-4.14467221464194	-1.14448266032734	-1.81310431790357
H	-6.04943148230643	0.39600122656974	-1.70774894300694
H	-6.22309659263509	2.05620926865064	0.12594974922572
H	-4.45350816857682	2.15662232080065	1.85939140208728
H	-2.53570208839308	0.61604218953992	1.75090705224802
H	-1.38680496889006	-1.18985263117370	0.82520313478283

H	-0.80571419543801	-2.77118875077175	-0.87484485191571
H	-2.22585626033111	-2.25740723325986	-1.92775866790823

47

I-3

Fe	1.36862889686240	-0.17730712413772	-0.44357581551541
O	0.64529849267831	0.54136871181262	1.29875766180135
C	0.02686547276965	1.63247846376621	1.52454333631131
C	-0.25301745316247	2.61138667789017	0.56831702842111
C	0.19467253422144	2.58664662457146	-0.76455982905734
O	0.94836110533752	1.69671755502564	-1.25604254193291
C	-0.26643580334212	3.67761308476154	-1.69990004323408
H	-0.94541062983701	3.23788294540518	-2.43604492419853
H	-0.78911047990499	4.47727141935534	-1.17730622156896
H	0.58550337988719	4.08661478199229	-2.24558006288238
H	-0.85013482098295	3.45532144876283	0.87980146037899
C	-0.41779876614490	1.85580187985902	2.94781291816081
H	-1.16834208537240	2.64061498193706	3.02693229090090
H	-0.80942232935633	0.92686601158884	3.36332285547815
H	0.44983222268787	2.14709200587308	3.54709495716214
O	1.48010949979647	-2.16484807679909	0.23267143467759
C	2.53178080780530	-2.83791588262076	0.42600037982368
C	3.81885903637520	-2.43796186615331	0.02287693346646
C	4.10411425075083	-1.28323148658844	-0.71093589857572
O	3.24417266293402	-0.42308228492362	-1.10084700606906
C	5.53170440946449	-1.01991350642637	-1.11753984484341
H	6.23269990279293	-1.67740256581247	-0.60659325355027
H	5.63046841488050	-1.17423663216934	-2.19608172367133
H	5.78509001874812	0.02075235197928	-0.90617371104734
H	4.64764606598067	-3.08119945196398	0.27979946528586
C	2.36516607851939	-4.17118757932343	1.11662601356325
H	1.69765950046684	-4.05992961546947	1.97167727008894

H	3.31497395445046	-4.59196192212512	1.44451434699079
H	1.89357533236580	-4.87083092820106	0.42082655276171
N	-0.51205560000461	-0.71522917148553	-1.37564627007112
H	-0.87790419828991	0.05756463638448	-1.92272528665701
C	-1.29178710542018	-1.93894093116647	-1.60538575572106
C	-1.52213297880105	-1.15639741160554	-0.37076318695207
C	-2.68764180955768	-0.27671210379106	-0.12174247716822
C	-3.03400539129140	0.01449879610818	1.19742224147300
C	-4.05630466167822	0.91261139115863	1.48173185833843
C	-4.74742408267981	1.53115199764551	0.44591512666621
C	-4.41163152383531	1.24244312946215	-0.87383056503837
C	-3.39112166432247	0.34475033167829	-1.15536563780380
H	-3.13765948469999	0.14069979171053	-2.18911232500481
H	-4.94534979299655	1.71956218329712	-1.68597084806515
H	-5.54070765480079	2.23458354741877	0.66312796646853
H	-4.30654379183691	1.13966777343586	2.51075715555482
H	-2.47724838035035	-0.45013536620881	2.00183442309938
H	-1.04942047819794	-1.53816930017801	0.52438908762498
H	-0.72262722787502	-2.85634551321797	-1.53808593218747
H	-2.03476661221421	-1.89794836612738	-2.39203556947823

18

I-4

C	-1.45163259035846	-1.10517036397107	-0.36576966585700
C	-3.00965797558600	0.04162173539644	1.20546003451400
C	-3.32465143033212	0.39639898879522	-1.14715056045727
H	-0.98410447726864	-1.49345692635660	0.53276143237630
C	-4.77736611977327	1.48984116505576	0.43618396389038
C	-2.61572258103134	-0.21647263008695	-0.10923492728505
H	-2.46589315153726	-0.42165648660045	2.02072565406184
H	-5.61222529620521	2.14636720528771	0.64442832780192
H	-4.36939667769518	1.08235088122874	2.50349525449431

H	-4.93223637860658	1.69911316620527	-1.69761814662706
H	-3.04294409494580	0.21818374012757	-2.17811924462409
H	-0.85912061140333	-2.89549232963853	-1.55400908470182
H	-2.17236290423702	-1.86842334698035	-2.30548982910478
C	-4.07980203215461	0.88881393711538	1.47777076638088
C	-4.39400498491060	1.23913273132503	-0.87851146275437
C	-1.35622431046956	-1.93337826259582	-1.59522839137409
H	-0.82356571109720	0.01324910833476	-1.96823028485098
N	-0.47541867238782	-0.77339231264210	-1.42696383588312

36

I-5

C	0.13366181591666	-2.19552264719842	-0.24849969697085
C	-2.02298204392701	-1.93084265752366	0.91678522966964
C	-0.88902957408967	0.06865720605706	0.19855513544447
H	-0.00581403847600	-3.24928213883832	-0.04751349280619
C	-2.98570349069104	0.22458924486884	1.37895479471973
C	-0.94104310828558	-1.32240355806151	0.28147438504242
H	-2.06470770036312	-3.01106251559212	0.98761241460536
H	-3.77676411811028	0.82783927822928	1.80561901004931
H	-3.87219590248825	-1.64379748032371	1.96427395907013
H	-1.86336859565838	1.91364069008442	0.66564999218308
H	-0.05574409821084	0.56258833785669	-0.27719744784713
H	2.32004293432560	-2.49243735301704	-0.37563247325361
H	1.79070652179895	-0.73630096246301	-0.35025460323403
C	-3.04013568238898	-1.16109306768188	1.46834636855889
C	-1.91004436834308	0.83519398745736	0.74036505819826
C	1.52446690305008	-1.77153635551165	-0.48725975128081
H	0.35494514092534	-1.13181439929483	-2.19058423562201
N	0.63671024130736	-1.98732315766452	-1.65630869024833
H	0.78571954597807	-2.81209491499454	-2.22495477082300
O	1.79673246154550	-0.57523912129801	2.32123584160993

H	1.87872923120656	0.32460900794988	1.95084201080018
C	0.81753853982918	-0.55368959053039	3.34834890815211
H	-0.15759582429136	-0.22057467396420	2.97774221993301
H	1.11517249429532	0.09140771802981	4.18302428368393
H	0.71062903892405	-1.57155695840275	3.72550758985742
O	2.04815965342286	1.95923720627017	1.10533401523575
H	1.98106534290244	1.88054590658200	0.13666724232027
H	1.41282657761788	2.64054914954540	1.35256744687027
S	1.10726600501808	1.35317662951437	-2.95564089654455
O	1.74777044692184	1.37726284915588	-1.64382138673148
O	0.16510205650575	0.23653974549459	-3.10659534687239
O	1.96900525868702	1.58440865926874	-4.08609310340423
C	-0.01895525859233	2.83092729950608	-2.92366210092490
F	-0.86624423272252	2.76513646176407	-1.88303828078852
F	0.69330851592985	3.95853448704878	-2.80783994142676
F	-0.74246172855341	2.89903957620937	-4.04591576408708

36

TS-4

C	0.34448314728270	-1.86239779389222	0.30510150907031
C	-1.87456575840229	-1.79227810780025	1.31161379180798
C	-1.11650882302799	0.13225466722321	0.04665927857740
H	0.43494830846828	-2.84171788067116	0.75623698583284
C	-3.27643340685190	0.14612504197873	1.10821030833934
C	-0.88782597734395	-1.16428776758478	0.53224863154899
H	-1.69540181325892	-2.79191971797621	1.68754905959547
H	-4.20527802600617	0.65697410126740	1.32815493062782
H	-3.81864776500506	-1.62803805681502	2.19688692516506
H	-2.47827052043400	1.77555853542661	-0.04879168448659
H	-0.37606703233218	0.61733067359489	-0.56559460722216
H	2.35961562465070	-1.96699933313437	-0.43283205536134
H	1.50149264315429	-0.49285302009894	-0.94602861618133

C	-3.06138893068023	-1.14002408466482	1.59788235384631
C	-2.30444874870165	0.77907833058860	0.33446751024551
C	1.39402823174443	-1.53321262185948	-0.67271547078074
H	0.20665515160176	-1.70366885415298	-2.36998393743570
N	0.64410004203987	-2.28782717672021	-1.66699674674283
H	1.04913453321369	-3.14922017665225	-1.99800521738009
O	1.57056962165413	-0.93287900470941	2.07204773518945
H	1.77217276881612	-0.06848166859564	1.64205094207810
C	0.83658440783384	-0.67303491392488	3.26390364796560
H	-0.11497394174470	-0.17625923114384	3.05097686850920
H	1.41895845354208	-0.04842095203882	3.94643077585175
H	0.63419423746226	-1.62915170975350	3.74478999497667
O	2.04163393858923	1.49229477894833	0.85868635686036
H	2.11516871762019	1.54359924051003	-0.11805595770373
H	1.42990360490912	2.19564675863514	1.10441973136946
S	1.21336113796081	1.51701934901484	-2.98586283752761
O	2.21772053336437	1.50892564985967	-1.91905533448300
O	0.23277689130375	0.44341437152984	-2.87701044474737
O	1.72918779838058	1.76879286147387	-4.30909457826649
C	0.21160466714837	3.03517531253843	-2.58246361163899
F	-0.27398545723172	2.96151124725206	-1.32270819045836
F	0.95696829912344	4.14355078886570	-2.66288076997579
F	-0.83146655884326	3.16542036348142	-3.41023727706554

24

P (product)

H	0.95944364932878	-1.33584543694761	-6.76686854952767
N	1.60405364205064	-1.59444508991112	-2.45967064731969
H	2.34193919724333	-2.01895912979771	-1.91065625465681
H	1.35829762699834	0.90955861086333	-7.72124964090878
H	0.84169551090623	2.93460575431164	-6.39430231829675
H	-0.80522068625079	-1.92230993601666	-3.55769142548848

H	2.06442931512446	-2.54146476470815	-4.34249366209775
H	-0.10399249061985	2.69630080267025	-4.11371218545555
C	0.13383598545527	1.81631586195489	-4.69822113537890
C	0.95075597183998	0.81292605532661	-6.72238915714797
C	0.19310706889951	-0.59549403919692	-4.90827253576395
C	0.72742941036476	-0.45096183125686	-6.18849942868609
C	-0.10511388324324	0.55016864628356	-4.17281271370533
H	-0.52489389324459	0.44961271982566	-3.17941606594808
C	0.00019480565903	-1.97192050271911	-4.30136957101857
C	1.26720614413949	-2.45841660007738	-3.59164726646938
H	1.06458711931222	-3.45976904434090	-3.20761062356813
O	-0.31193117376931	-2.95723901817285	-5.27787345623438
C	-1.63513867369877	-2.86757652407849	-5.77775043084579
H	-2.36703932342253	-3.04023824227996	-4.97978368436576
H	1.95931372706193	-0.70263088762692	-2.78797129285331
C	0.66141980763099	1.95167233304915	-5.97717630708147
H	-1.83104735015469	-1.89201348047548	-6.23514106048897
H	-1.74494150761122	-3.64209625667896	-6.53544058669237

48

[Int I-H]+ (Fe(N+O)

Fe	0.58128770517502	0.45434582423336	-0.23487524701179
O	1.78882348034866	0.30486695619772	1.25759684889191
C	2.81385504583910	-0.45713589086006	1.40958223268727
C	3.34531575348639	-1.24937874386396	0.39434254251041
C	2.85678580437805	-1.29548543753483	-0.91812407605047
O	1.82575031695801	-0.65952856905289	-1.32506636109073
C	3.57310497116211	-2.11340524543075	-1.94864881212486

H	4.02553738474255	-1.43731287687339	-2.67930923572097
H	4.35132899675646	-2.73367529630146	-1.51050030306228
H	2.85669923882115	-2.73868770940548	-2.48435176728754
H	4.21949183034724	-1.83876457960994	0.62288520232000
C	3.44178628110571	-0.43800883774263	2.76842645471250
H	4.32041711722221	-1.07625910361062	2.82118417272285
H	3.71864969337839	0.58642715068259	3.02747522143598
H	2.70688260141150	-0.77220778881341	3.50475412958962
O	-1.03802399451559	1.29764223651658	0.59768575500531
C	-1.52379387183597	2.45631991255917	0.37074518617526
C	-0.85734231425691	3.45534863469232	-0.34847003620485
C	0.39293445857673	3.29713772448492	-0.94373516566747
O	1.03403786948542	2.18458135360212	-0.98906420433352
C	1.06324242989098	4.46342628223798	-1.59770094928909
H	0.60316717826565	5.40546319762548	-1.30838191545323
H	2.12108014235095	4.46805722145161	-1.33125411643579
H	0.99333874157802	4.35366638557826	-2.68379569125545
H	-1.32427534687700	4.42542490655863	-0.41871915391994
C	-2.89552212797965	2.70994039046800	0.91642584780334
H	-2.91594003720691	2.47878496094726	1.98334608812225
H	-3.59414749805193	2.02702779341798	0.42760647063966

H	-3.22232948918496	3.73335476113034	0.74738403062176
N	-0.86612817121447	-0.10071578272863	-1.88766846872604
H	-0.41727286521714	-0.64573259037488	-2.62327890447028
H	-1.37842575482381	0.66481568950642	-2.32029279824720
O	-1.90780680156203	-0.93957510461679	-1.36289759165652
C	-1.57908340836885	-1.50322507363601	-0.18492254369356
O	-0.48250399213155	-1.33365220221124	0.31916951314593
C	-2.70777333294374	-2.28150480667919	0.44593940453170
C	-3.53283370483709	-3.03579928319584	-0.60299065641597
H	-2.91116989233996	-3.73503055859018	-1.16484451489070
H	-4.30678935721205	-3.60467548810673	-0.08737009309764
H	-4.01635948252903	-2.35427419274113	-1.30170809978525
C	-3.59089617062344	-1.22680115091905	1.15272156589440
H	-3.01113759476711	-0.64437175277369	1.86938251107185
H	-4.38243624867516	-1.75192955226047	1.68825732968874
H	-4.04779682560813	-0.54830639642908	0.43210481150937
C	-2.12204439045096	-3.25221043739113	1.47585180517069
H	-1.47482198424885	-3.98761168827403	0.99569000843554
H	-1.54378774487043	-2.72465590921823	2.23314066154700
H	-2.93981378394757	-3.78038581134507	1.96674342635784
